# Profound gene expression changes in the epithelial monolayer of active ulcerative colitis and Crohn’s disease

**DOI:** 10.1371/journal.pone.0265189

**Published:** 2022-03-11

**Authors:** Siri Sæterstad, Ann Elisabet Østvik, Elin Synnøve Røyset, Ingunn Bakke, Arne Kristian Sandvik, Atle van Beelen Granlund

**Affiliations:** 1 Department of Clinical and Molecular Medicine, Norwegian University of Science and Technology (NTNU), Trondheim, Norway; 2 Department of Gastroenterology and Hepatology, Clinic of Medicine, St. Olav’s University Hospital, Trondheim, Norway; 3 Department of Pathology, St. Olav’s University Hospital, Trondheim, Norway; 4 Clinic of Medicine, St Olav’s University Hospital, Trondheim, Norway; 5 Centre of Molecular Inflammation Research, Norwegian University of Science and Technology (NTNU), Trondheim, Norway; University of New Mexico, UNITED STATES

## Abstract

In recent years it has become apparent that the epithelium is highly involved in inflammatory bowel disease (IBD) pathophysiology. The majority of gene expression studies of IBD are generated from heterogeneous biopsies, providing no distinction between immune cells, the epithelium and other mucosal cells. By using laser capture microdissection (LCM) coupled with RNA sequencing, we aimed to characterize the expressional changes of the isolated colonic epithelial monolayer from ulcerative colitis (UC) and Crohn’s disease (CD) patients compared to healthy controls (HC). The analysis identified 3706 genes as differentially expressed between active IBD epithelium and HC. Weighted gene co-expression network analysis was used to stratify genes into modules, which were subsequently characterized using enrichment analysis. Our data show a distinct upregulation of the antigen presentation machinery during inflammation, including major histocompatibility complex class II molecules (e.g. *HLA-DPA1*, *HLA-DPB1*, *HLA-DRA*) and key transcription factors/activators (*STAT1*, *IRF1*, *CIITA*). We also see an epithelial downregulation of retinoic acid-responsive nuclear receptors (*RARA*, *RARB*, *RXRA)*, but upregulation of retinoid-metabolizing enzymes (*RDH11*, *ALDH1A2*, *ALDH1A3*), which together suggest a perturbation of epithelial vitamin A signaling during active IBD. Lastly, we identified a cluster of stress-related genes, including activator protein 1 components *JUNB* and *ATF3*, as significantly upregulated in active UC but not in CD, revealing an interesting aspect of IBD heterogeneity. The results represent a unique resource for enhanced understanding of epithelial involvement in IBD inflammation and is a valuable tool for further studies on these processes.

## Introduction

Characterization of whole genome gene expression in inflamed intestinal biopsies compared to healthy controls (HCs) has contributed greatly to increased understanding of the complexity and heterogeneity of inflammatory bowel disease (IBD). However, our knowledge of intestinal gene expression in IBD is largely based on sequencing of whole biopsy homogenates, providing no distinction between expressional signals among epithelial cells, immune cells and other mucosal cells [1]. As the composition of mucosal cell populations differs between healthy individuals and IBD patients, important gene regulation might be overlooked or wrongly attributed when analyzing heterogenous tissue samples. Our group and others have found the epithelial monolayer to be involved in inflammatory activity and regulation far beyond its previously perceived role as a passive barrier towards the luminal milieu [2, 3]. As patient-derived organoids have improved our ability to study epithelial molecular mechanisms, we have a newfound opportunity to study inflammatory responses in an *in vitro* model faithfully reproducing *in vivo* mechanisms [4]. With this, our need to better understand epithelial responses to inflammation has become clear.

The chemical and physical barrier properties of the intestinal epithelium provide spatial separation of luminal contents and microbiota from the underlying immune system, which is a crucial feature in maintaining homeostasis. In IBD patients this homeostatic relationship is disturbed, characterized by a change in the microbiome, a breakdown of the epithelial barrier, and excessive inflammatory responses [2]. The epithelial monolayer is composed of functionally different intestinal epithelial cell (IEC) linages including enterocytes, goblet cells (GCs), enteroendocrine cells, Tuft cells, Paneth cells and M cells, all of which originate from epithelial stem cells located at the base of intestinal crypts [5]. In IBD, the abundance, activity and location of these cell types is altered [6, 7]. A wide range of antimicrobial peptides is secreted by GC and Paneth cells, helping to combat microbial colonization, and the expression of many of these peptides is regulated during inflammation [8]. In addition, studies in mice have shown that GCs are capable of sensing and delivering luminal content to underlying immune cells through goblet cell-associated antigen passages, a central process in the induction and maintenance of tolerance to harmless dietary antigens and commensal microbiota [9, 10]. In colonic IBD, both enteroendocrine cell numbers and activity are altered, with more serotonin^+^-cells in the mucosa [11, 12]. We and others have recently shown that epithelial serotonin reuptake is regulated in IBD, with reduced expression of the serotonin reuptake transporter (SERT), possibly resulting in increased levels of extracellular serotonin [13, 14]. The chronic inflammation in IBD is associated with an increased risk of colorectal cancer, particularly in UC, and successful treatment of IBD appears to reduce the colitis-associated colorectal cancer risk [15]. Key factors in the formation of colitis-associated colorectal cancer are the important immune regulators NFκB and Activator protein 1 (AP-1) components [16, 17]. As most new and emerging IBD treatments target pro-inflammatory regulators, it is a crucial balancing act to inhibit pathological inflammatory signaling, while retaining the antitumor immunity. Increased understanding of IEC signaling during inflammation can aid the selection of suitable targets for treatment.

In recent years, single-cell RNA sequencing (scRNA-Seq) has provided valuable knowledge about cell characteristics in both health and disease, previously unattainable by traditional methods. Single cell analysis of the intestinal epithelium has enabled determination of cell compositions and identification of rare cell types, and insight into cell-type specific expression programs as well as revealing linage relationships of different cell types [7, 18, 19]. However, scRNA-Seq analysis is expensive, results in comparatively noisy and sparse data, and requires the researcher to prioritize either cell number or sequencing depth when planning experiments [20]. Most scRNA-Seq methods require fresh or cryopreserved cells as starting material, and depend on handling known to have an effect on sequencing results, leading to an underestimation of cell types and activation of cellular stress responses [21]. The method’s cost and complexity make it inaccessible to most researchers and the results often lend themselves poorly to global characterization of gene expression changes.

In this study, the aim was to characterize the epithelial inflammatory responses during colonic IBD. Through a combination of RNA-Seq on microdissected colon epithelium, weighted gene co-expression network analysis (WGCNA), and enrichment analysis, we characterized the expressional changes in IECs during inflammation. IHC was performed to verify epithelial protein expression of a selected set of differentially expressed genes (DEGs). Finally, we discuss some of the implications of the observed changes, with an emphasis on novel observations of DEGs involved in immune regulation, cellular stress and Vitamin A signaling. Together, this analysis provides a solid basis for further studies on epithelial involvement in IBD pathophysiology.

## Results

### Analysis pipeline

**Fig 1:**
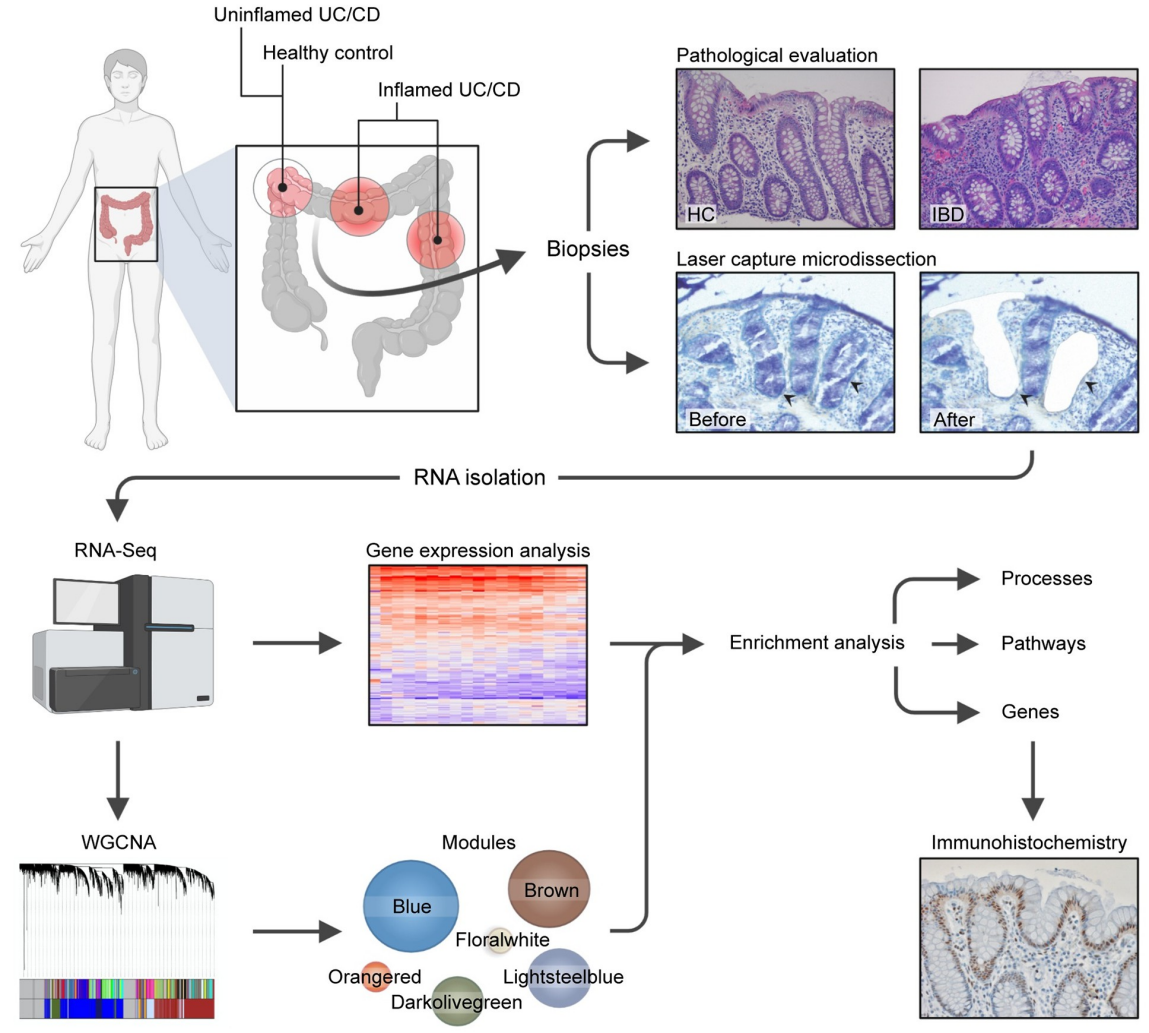
Schematic project workflow. Uninflamed Inflammatory bowel disease (IBD; Ulcerative colitis (UC) and Crohn’s disease (CD)) and healthy control (HC) samples were all taken from the hepatic flexure, while inflamed samples were taken from maximally inflamed, intact colon mucosa. All samples were evaluated by an experienced gastrointestinal pathologist to verify status as inflamed/uninflamed. An epithelial monolayer area corresponding to approx. 10 000 cells was isolated from each sample using laser capture microdissection. Total RNA was isolated, and mRNA sequenced. Results were then used to perform differential gene expression analysis and weighted gene co-expression network analysis (WGCNA) in parallel. Using significant modules identified by WGCNA in combination with differential expression identified by LIMMA voom, MetaCore was used to characterize each module with regards to process and pathway enrichment. For a selection of genes, epithelial protein expression was verified by immunohistochemistry. Figure created in and republished from BioRender under a CC BY license, with permission from Biorender, 2021.

A pipeline for analysis was established, coupling LCM with RNA-Seq, with subsequent WGCNA, differential expression and enrichment analysis, as outlined in Fig 1.

### Laser capture microdissection yielded good quality RNA

Samples were drawn from St. Olav’s University Hospital IBD biobank. While six samples from each group was initially selected, reclassification of two samples after finalized isolation and analysis resulted in the following sample distribution: six healthy control (HC), six uninflamed UC (UCu), five uninflamed CD (CDu), seven inflamed UC (UCa) and five inflamed CD (CDa). All samples in uninflamed groups (UCu/CDu) were taken from patients with no history of inflammatory activity at the sampling site. A summary of patient characteristics is given in Table 1. From each biopsy, an area of epithelial monolayer (S1 Fig) corresponding to approx. 10 000 epithelial cells was isolated, and RNA was extracted. DV200 measures were used to assess RNA integrity and ensuring RNA fragment length compatible with downstream RNA-Seq. Mean DV200 was 89 (75–95), well above suggested threshold of DV200 70%. Average RNA concentration of isolate was 145 ng/μL (107–146 ng/μL), and all samples were diluted to 18 ng/mL prior to library prep.

**Table 1:** Characteristics of IBD patients and controls included in the study.

				IBDa		IBDu	
	HC	IBDa	IBDu	UCa	CDa	UCu	CDu
***n***	6	12	11	7	5	6	5
**Median age (range)**	50 (35–68)	34 (20–66)	42 (20–66)	38 (21–66)	33 (20–46)	47 (33–60)	42 (20–55)
**Female sex**	1	7	5	5	2	4	1
**5-ASA/S-ASA**	0	4	6	2	2	3	3
**Steroids**	0	4	3	1	3	0	3

HC, healthy control; IBDa, active inflammatory bowel disease; IBDu, uninflamed IBD; UCa, ulcerative colitis with active inflammation; CDa, Crohn’s disease with active inflammation; UCu, ulcerative colitis from uninflamed area; CDu, Crohn’s disease (CD) from uninflamed area; 5-ASA, 5-aminosalicylic acid; S-ASA, sulphasalazine. Age is given as median, sex and medication as numbers.

### RNA-Seq captures biological difference in inflamed vs. uninflamed epithelium

**Fig 2:**
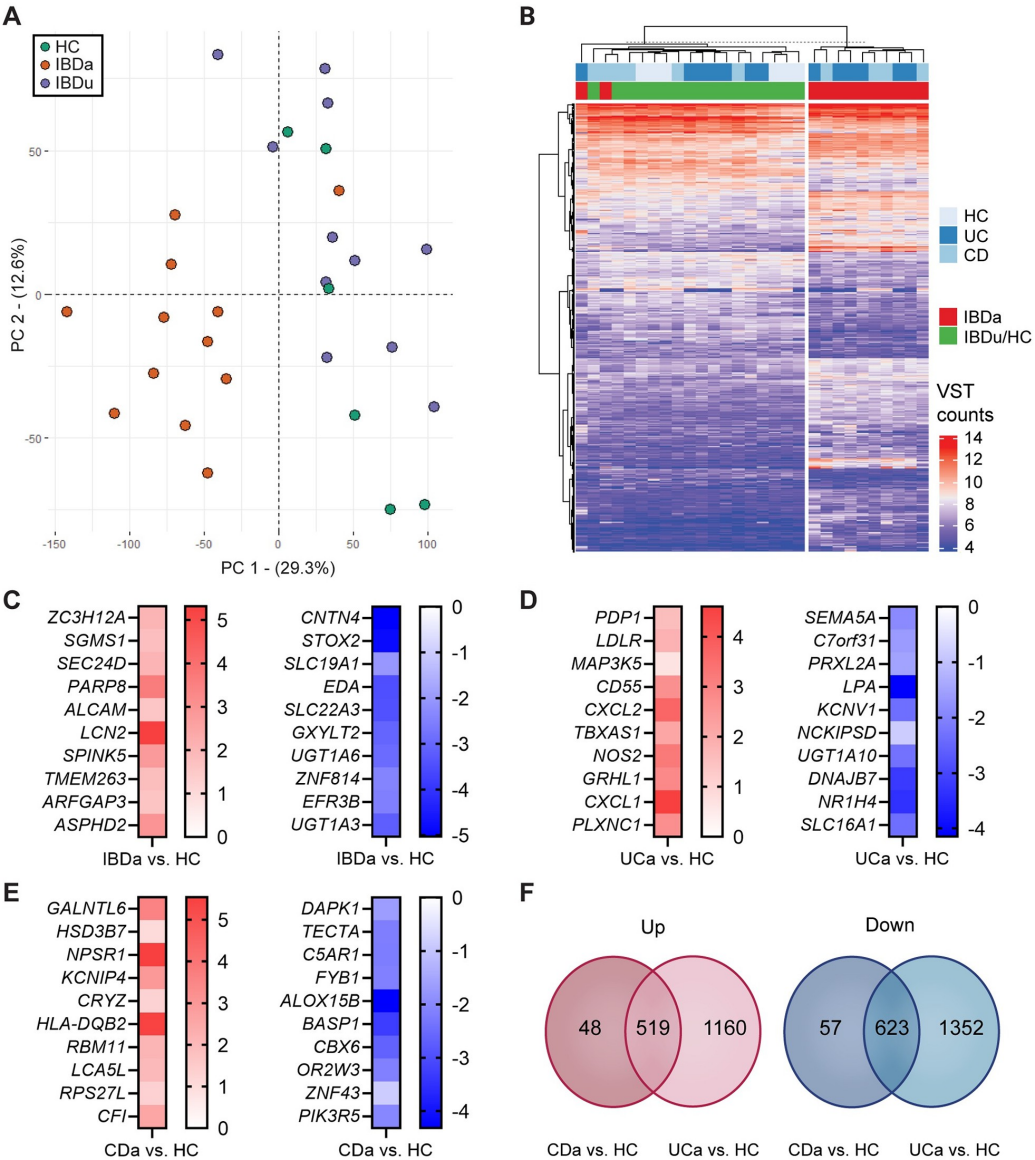
Epithelial gene expression in IBD—Quality control and initial analyses. **(A)** Principal component analysis reveals a clear distinction in gene expression signatures between inflamed and uninflamed samples. There was no separation between UCa and CDa (not shown), and no visual distinction between uninflamed IBD (IBDu) and healthy controls. **(B)** Unsupervised cluster analysis of top 1000 genes selected on variance as captured by RNA-Seq analysis. The heatmap shows a separation between inflamed and uninflamed samples, but no clustering of UCa or CDa. Counts were transformed by variance stabilizing transformation (VST) as described in the Methods section. **(C-E)** Top 10 up- and downregulated genes (by adj. p) in **(C)** IBDa vs. HC, **(D)** UCa vs. HC and **(E)** CDa vs. HC. **(F)** Venn diagram of upregulated (left) and downregulated (right) genes in CDa vs. HC and UCa vs. HC.

A principal component analysis plot of normalized RNA-Seq counts revealed a clear separation between inflamed and uninflamed samples along PC1, explaining 29.3% of the total variance in the data set (Fig 2A). An unsupervised cluster analysis of the 1000 genes with highest variance across all samples showed clear separation in inflamed IBD on one side, and uninflamed IBD and HC groups on the other (Fig 2B). There was no visual separation between UC and CD, either in the inflamed or uninflamed cohort. Differential expression analysis identified 3706 DEGs in the IBDa vs. HC contrast, including 1661 upregulated and 2045 downregulated genes. No genes were differentially expressed when contrasting uninflamed samples from IBD patients (UCu/CDu) with HC. Top 10 up- and downregulated genes (by adj. p) in the IBDa vs. HC contrast are presented in Fig 2C. The most significant GO processes (< 1500 genes, p < 0.05) included *Antigen processing and presentation* (GO:0019882), *Regulation of protein localization* (GO:0032880) and *Secretion* (GO:0046903). Top 10 up- and downregulated pathways (p < 0.05) are given in S2 Fig. In the contrasts UCa vs. HC and CDa vs. HC, 3654 and 1247 genes were differentially expressed, respectively. A total of 2512 genes (1160 up, 1352 down) were significant in UCa vs. HC, but non-significant when contrasting CDa vs. HC. In comparison, only 105 genes (48 up, 57 down) were significantly regulated in CDa vs. HC, but non-significant in UCa vs. HC. Top 10 up- and downregulated genes specific for UCa vs. HC and CDa vs. HC (by adj. p) are presented in Fig 2D and 2E. See Fig 2F for Venn-diagram of shared and contrast-specific genes. Complete results from differential expression analysis are given in S1 File.

Gene Set Enrichment Analysis (GSEA ver 4.1.0, build 27) was used in order to identify changes in epithelial differentiation based on gene sets derived from single cells sequencing of colonic epithelium [7, 19, 22].

### WGCNA identifies clusters of co-expressed genes

**Fig 3:**
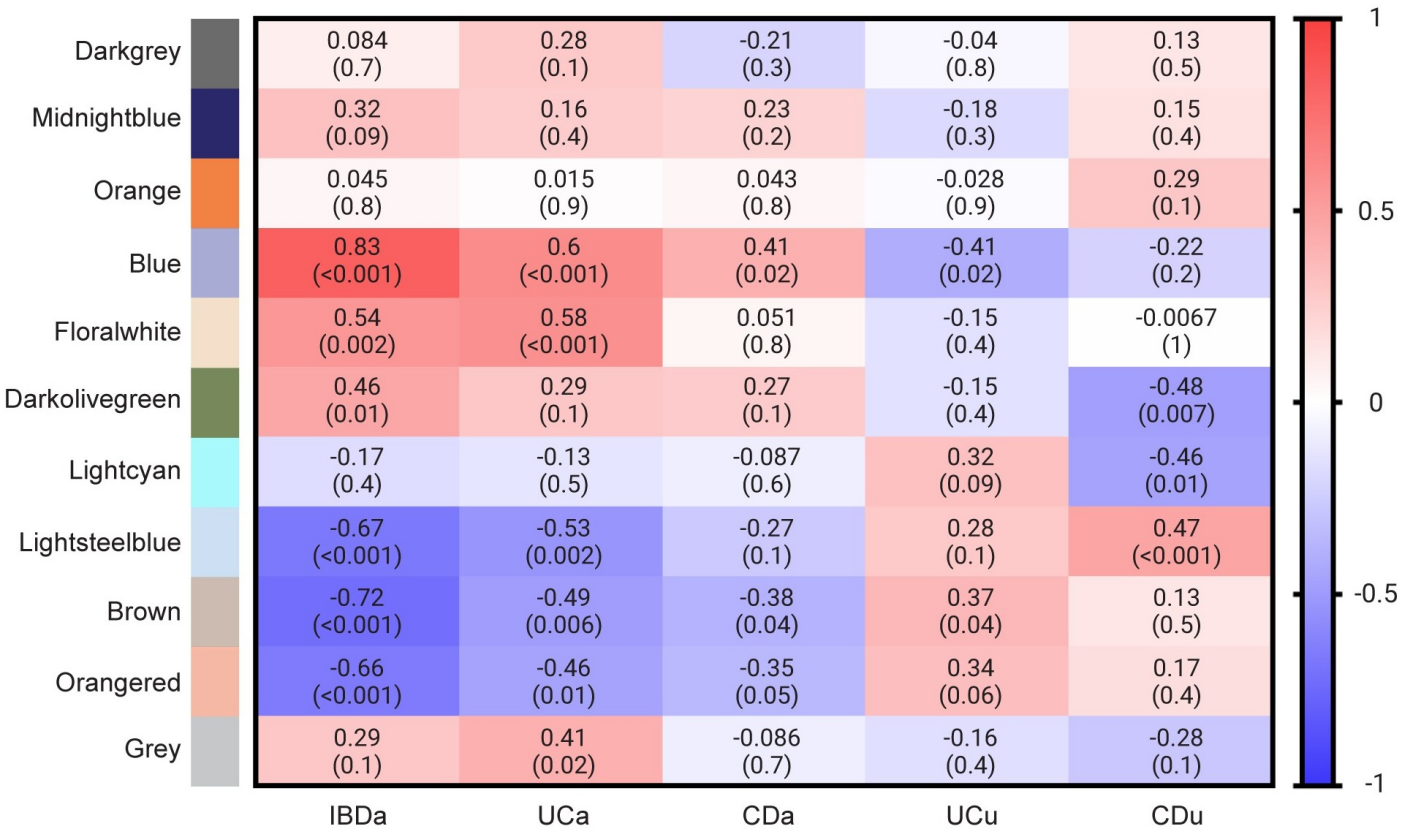
Module-trait relationships. Matrix showing the correlation between WGCNA modules (*n* = 10), and the sample categories IBDa, UCa, CDa, UCu and CDu. Undesignated genes were collected in the Grey module. Significant correlation between module and trait suggests that genes within the module display expression levels that correlate with sample trait. For each contrast, correlation coefficient is shown, with p-values in parentheses.

**Fig 4:**
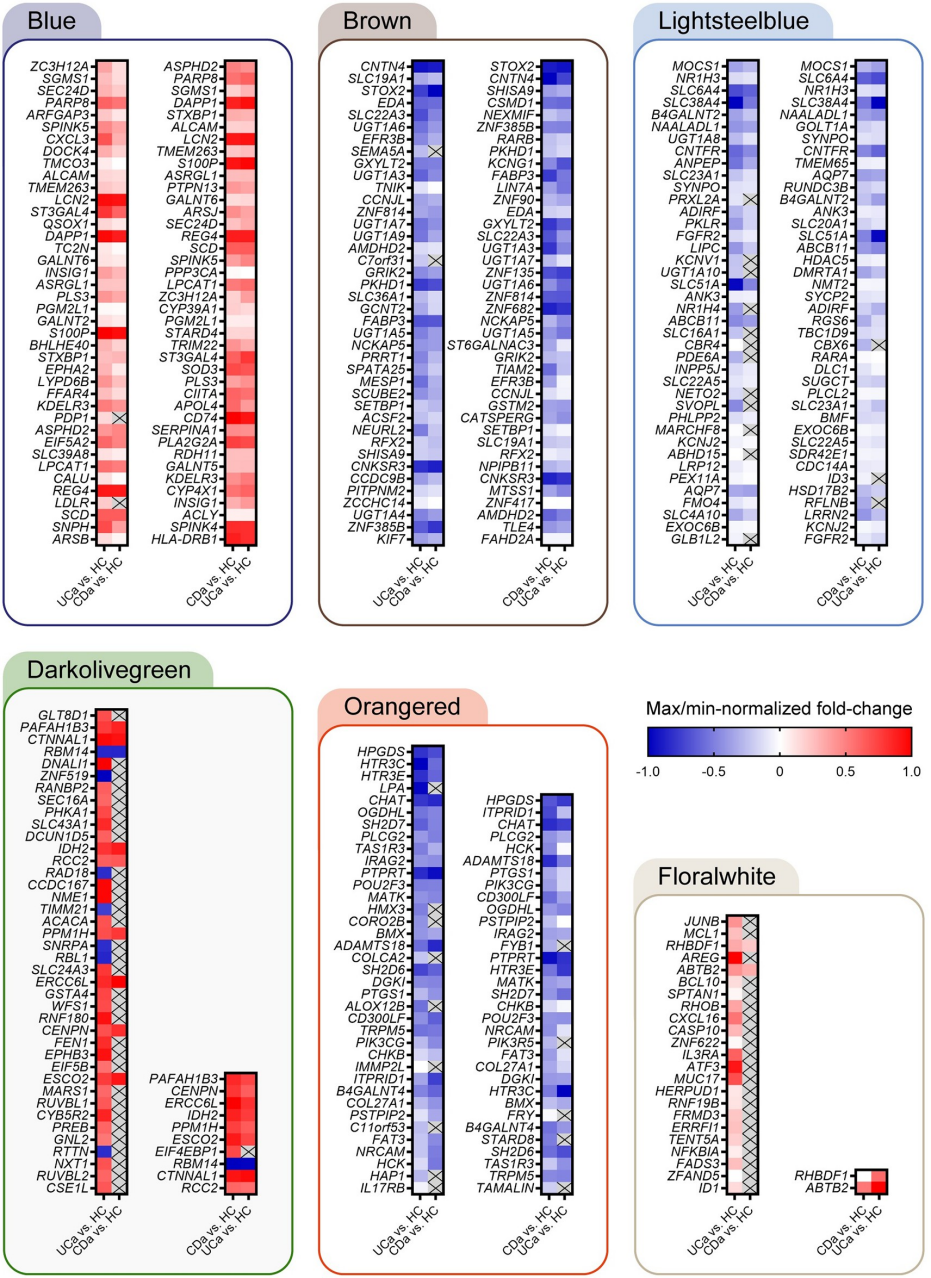
Top differentially expressed genes (by adj. p) for WGCNA modules where expression pattern was significantly correlated with disease status. Using data from the differential expression analysis coupled with module memberships identified through WGCNA, the most significant differentially expressed genes for the contrasts UCa vs. HC (left) and CDa vs. HC (right) were selected. For contrasts with > 40 DEGs, top 40 is presented. For each module, results for both contrasts are shown. The fold-change was max/min-normalized within each module for visualization purposes. No fold-change is visualized (marked with X) for genes where the adjusted p-value was > 0.05. Fold-change and adj. p was found using LIMMA linear models with least squares regression and corrected for multiple testing using Benjamini–Hochberg FDR-adjustment.

WGCNA was used to identify clusters of genes (called modules) that co-expressed within the expression data, and whether expression levels within these clusters were correlated with inflammatory status. Correlation of module membership score vs. gene significance in the contrast IBDa vs. HC is presented in in S3 Fig. The analysis resulted in a final set of 10 modules ranging in size between 48 to 3966 genes, in addition to 2522 genes not designated to any module (collected in the Grey module). These modules were subsequently used in a supervised analysis, identifying how gene expression within each module correlates with the sample groups IBDa, UCa, CDa, UCu and CDu. Module-trait relationships are presented in Fig 3. The gene expression pattern within six modules were found to significantly correlate with inflammatory status and were explored further using pathway analyses in MetaCore. In this pathway analysis, all genes within the six modules significantly correlated to IBDa were linked with differential expression analysis results. Fig 4 shows top DEGs (by adj. p) for each significant module, for the contrasts UCa vs. HC and CDa vs. HC.

### Enrichment analysis identifies dominating processes within modules significantly correlated with disease status

Complete results from MetaCore analysis for each module is found in S2 File with a pathway analysis report available in S3 File. With 3966 genes, Blue became the largest module significantly correlated with active IBD (IBDa vs. HC, p < 0.001). A total of 1467 genes grouped into this module were differentially expressed in the contrast IBDa vs. HC, and they were solely upregulated. The genes within Blue were primarily enriched in immune system processes, including top Gene Ontology (GO) processes (< 1500 genes); *Viral process* (GO:0016032), *Cell activation involved in immune response* (GO:0002263) and *Protein localization to endoplasmic reticulum* (GO:0070972). Top three enriched pathways were *Immune response_Antigen presentation by MHC class I*, *classical pathway* (FDR < 0.001), *COVID-19*: *immune dysregulation* (FDR < 0.001) and *Immune response_Induction of the antigen presentation machinery by IFN-gamma* (FDR < 0.001). In addition to major histocompatibility complex (MHC) class I-related genes, key players in MHC class II (MHCII) presentation were upregulated in IBDa, including *STAT1*, *IRF1*, *CIITA*, *CD74* and *CD40* (see Table 2 for log_2_ fold-change (LFC) and adjusted p-values for these and all subsequently mentioned differential expressions). Upregulated MHCII molecules include classical *HLA-DPA1*, *HLA-DPB1* and *HLA-DRA*, and non-classical *HLA-DMA* and *HLA-DMB*. Genes encoding retinoic acid (RA) metabolizing enzymes were also upregulated in active IBD; *RDH11*, *ALDH1A2* and *ALDH1A3*. Of note, three AP-1 transcription factors within Blue were differentially expressed in UCa but non-significant in CDa; *MAFF*, *FOS* and *MAFB*.

**Table 2:** Log_2_ fold-change (LFC) and adjusted p-values (adj. p) for differentially expressed genes within each WGCNA module for the contrasts IBDa vs. HC, UCa vs. HC and CDa vs. HC.

		IBDa vs. HC		UCa vs. HC		CDa vs. HC	
Module	Gene	LFC	Adj. p	LFC	Adj. p	LFC	Adj. p
Blue	*STAT1*	1.25	0.002	1.21	0.005	1.30	0.022
	*IRF1*	1.48	0.001	1.51	0.001	1.45	0.016
	*CIITA*	3.58	0.000	3.44	0.000	3.72	0.002
	*CD74*	5.52	0.000	5.31	0.000	5.73	0.002
	*CD40*	3.37	0.002	3.35	0.003	3.40	0.011
	*HLA-DPA1*	6.33	0.002	6.22	0.002	6.44	0.007
	*HLA-DPB1*	5.55	0.001	5.40	0.002	5.71	0.006
	*HLA-DRA*	6.78	0.001	6.59	0.002	6.97	0.006
	*HLA-DMA*	3.15	0.000	3.04	0.001	3.27	0.003
	*HLA-DMB*	4.41	0.001	4.30	0.001	4.52	0.005
	*RDH11*	1.88	0.000	1.89	0.000	1.88	0.002
	*ALDH1A2*	5.38	0.007	5.46	0.006	5.30	0.024
	*ALDH1A3*	2.49	0.022	2.55	0.025	2.43	0.081
	*MAFF*	2.12	0.005	2.66	0.001	1.58	0.116
	*FOS*	3.13	0.003	4.14	0.000	2.13	0.130
	*MAFB*	1.18	0.152	2.09	0.013	0.27	0.845
Floralwhite	*RHBDF1*	1.28	0.001	1.48	0.000	1.08	0.037
	*ABTB2*	1.61	0.004	1.77	0.002	1.45	0.041
	*MCL1*	0.91	0.003	1.23	0.000	0.59	0.157
	*NFKBIA*	0.20	0.501	0.63	0.035	-0.23	0.595
	*JUNB*	1.21	0.005	1.78	0.000	0.64	0.287
	*AREG*	2.70	0.012	3.69	0.001	1.71	0.211
	*ATF3*	2.49	0.062	3.42	0.011	1.57	0.394
Darkolivegreen	*PAFAH1B3*	0.97	0.001	0.86	0.004	1.09	0.005
	*CTNNAL1*	1.27	0.004	1.31	0.004	1.23	0.037
	*PPM1H*	0.80	0.008	0.70	0.026	0.90	0.025
	*IDH2*	1.04	0.004	0.93	0.015	1.14	0.025
	*ERCC6L*	1.21	0.009	1.02	0.031	1.39	0.021
Lightsteelblue	*RARA*	-0.75	0.001	-0.68	0.006	-0.82	0.011
	*NR1H3*	-1.65	0.000	-1.75	0.000	-1.54	0.000
	*NR1H4*	-2.60	0.000	-3.37	0.000	-1.82	0.050
	*RXRA*	-0.61	0.016	-0.65	0.016	-0.56	0.110
Brown	*PITX2*	-8.08	0.000	-8.83	0.000	-7.33	0.005
	*RARB*	-3.82	0.000	-3.27	0.000	-4.37	0.000
	*NR1I2*	-0.97	0.043	-1.23	0.017	-0.71	0.307
	*EDA*	-3.47	0.000	-3.54	0.000	-3.40	0.000
Orangered	*PIK3CG*	-2.01	0.000	-1.68	0.005	-2.34	0.005
	*PLCG2*	-2.31	0.000	-2.15	0.000	-2.47	0.002

The table includes analysis results for all genes mentioned in the results section. Non-significant genes for specific contrasts are marked. Fold-change and adj. p was found using LIMMA linear models with least squares regression and corrected for multiple testing using Benjamini–Hochberg FDR-adjustment. IBDa, active inflammatory bowel disease; UCa, active ulcerative colitis; CDa, active Crohn’s disease.

Floralwhite (IBDa vs. HC, p = 0.002) came out as the smallest module with only 48 genes assigned, of which 22 and two genes were significantly upregulated in UCa and CDa, respectively. The genes significantly regulated in CDa, were also significant in UCa; *RHBDF1* and *ABTB2*. Thus, Floralwhite had a total of 21 genes that were differentially expressed in UCa, but non-significant in CDa. Top GO processes (< 1500 objects) included *Regulation of cellular response to oxidative stress* (GO:1900407) and sub-categories of *Response to stimulus* (progesterone (GO:0032570), hydrogen peroxide (GO:0042542), peptide (GO:1901652) and mechanical stimulus (GO:0009612)), with genes such as *MCL1* related to the former process, and *JUNB*, *AREG* (Amphiregulin), *NFKBIA* (IκBα) and *BCL10* to the latter. All these genes were only differentially expressed in the UCa vs. HC contrast, together with *ATF3*, which is associated with both processes. Enrichment analysis identified *Immune response_IL-5 signaling via JAK/STAT* (FDR = 0.008) as the most enriched pathway, a pathway involving both MCL1, IκBα, JunB and Amphiregulin.

With its 836 assigned genes, Darkolivegreen was a medium sized module identified as significantly correlated with active IBD (IBDa vs. HC, p = 0.01). As isolated groups however, neither UCa nor CDa showed a significant correlation with this module (p = 0.1 for both UCa vs. HC and CDa vs. HC). Only 36 genes were differentially expressed in the contrast IBDa vs. HC (31 upregulated, five downregulated), revealing a marginal significant regulation when considering the size of the module. This suggests that Darkolivegreen plays a minor role in explaining the epithelial response to inflammation, compared to the other modules. Enriched GO terms were related to *Cell Cycle* (GO:0007049), including *Mitotic cell cycle process* (GO:1903047). A total of nine genes were differentially expressed in both UCa and CDa, including *PAFAH1B3*, *CTNNAL1*, *PPM1H*, *IDH2* and *ERCC6L*.

Out of the 1121 genes grouped into Lightsteelblue (IBDa vs. HC, p < 0.001), 242 were significantly downregulated in IBD. As in Floralwhite, the correlation between Lightsteelblue and CDa alone was not significant (p = 0.1). In contrast to Floralwhite however, the correlation was strengthened for Lightsteelblue when pooling UCa and CDa (Fig 3), suggesting similar processes within the UCa and CDa group of samples. Enriched GO terms (< 1500 objects) were mainly related to *Organic acid metabolic process* (GO:0006082) and *Cellular lipid metabolic process* (GO:0044255). Enrichment analysis identified *Regulation of lipid metabolism_RXR-dependent regulation of lipid metabolism via PPAR*, *RAR and VDR* as being the most enriched pathway within this module (FDR < 0.001). This pathway describes the interaction of nuclear receptors involved in transcriptional regulation, e.g. retinoic acid receptor alpha (RARα, encoded by *RARA*), in response to ligand binding, e.g. retinoic acid. Other genes worth mentioning are nuclear receptor subfamily 1 group H member 3 (*NR1H3*) encoding liver X receptor alpha (LXRα), and *NR1H4* encoding farnesoid X receptor (FXR). Common for RARα, LXRα and FXR is their ability to form heterodimers with retinoid X receptor alpha (RXRα, encoded by *RXRA*) to regulate metabolic processes, including lipid metabolism.

The Brown module showed the most significant negative correlation to inflammatory status (IBDa vs. HC, p < 0.001, S3 Fig). Out of the 3793 assigned genes, 1603 were differentially expressed in IBDa, of which all were downregulated. Top GO processes (< 1500 genes) were related *Chromatin organization* (GO:0006325), *RNA biosynthetic process* (GO:0032774) and *Phosphorylation* (GO:001631). Top three enriched pathways were *Apoptosis and survival_IL-17-induced CIKS-dependent NF-kB signaling and mRNA stabilization* (FDR < 0.001), *Chemotaxis_Lysophosphatidic acid signaling via GPCRs* (FDR < 0.001) and *Development_Negative regulation of WNT/Beta-catenin signaling in the nucleus* (FDR < 0.001). *PITX2* was the most downregulated gene among all modules (by LFC). Other differentially expressed transcription factors in IBDa were nuclear receptors retinoic acid receptor beta (*RARB*) and the pregnane X receptor (PXR)-encoding gene *NR1I2*. Like nuclear receptors RARα, LXRα and FXR (Lightsteelblue), RARβ and PXR are also able to form heterodimers with RXRα. Another gene of interest was *EDA* encoding the TNF family member ectodysplasin A. EDA is a cleavable membrane protein thought to be important in processes like wound healing [23]. Of note, a total of 181 genes designated as zinc finger motif (*ZNF*)-genes were downregulated in IBDa, constituting 11% of all differentially expressed genes within Brown.

Orangered (IBDa vs. HC, p < 0.001) was a small module containing only 61 genes, where all significant genes in IBDa (45) were downregulated. Seventeen genes encoding enzymes, including lipid kinase *PIK3CG* (PI3Kγ) and phospholipase *PLCG2* (PLCγ2) were differentially expressed. Top GO terms were related to *Glycerophospholipid biosynthetic process* (GO:0046474) and *Platelet activation* (GO:0030168), in which PI3Kγ and PLCγ2 play a role in both. Top three enriched pathways were *Cell adhesion_Integrin inside-out signaling* (FDR < 0.001), *Protein folding_Membrane trafficking and signal transduction of G-alpha (i) heterotrimeric G-protein* (FDR = 0.001) and *Chemotaxis_SDF-1/CXCR4-induced chemotaxis of immune cells* (FDR = 0.003).

### IHC confirms epithelial protein expression

**Fig 5:**
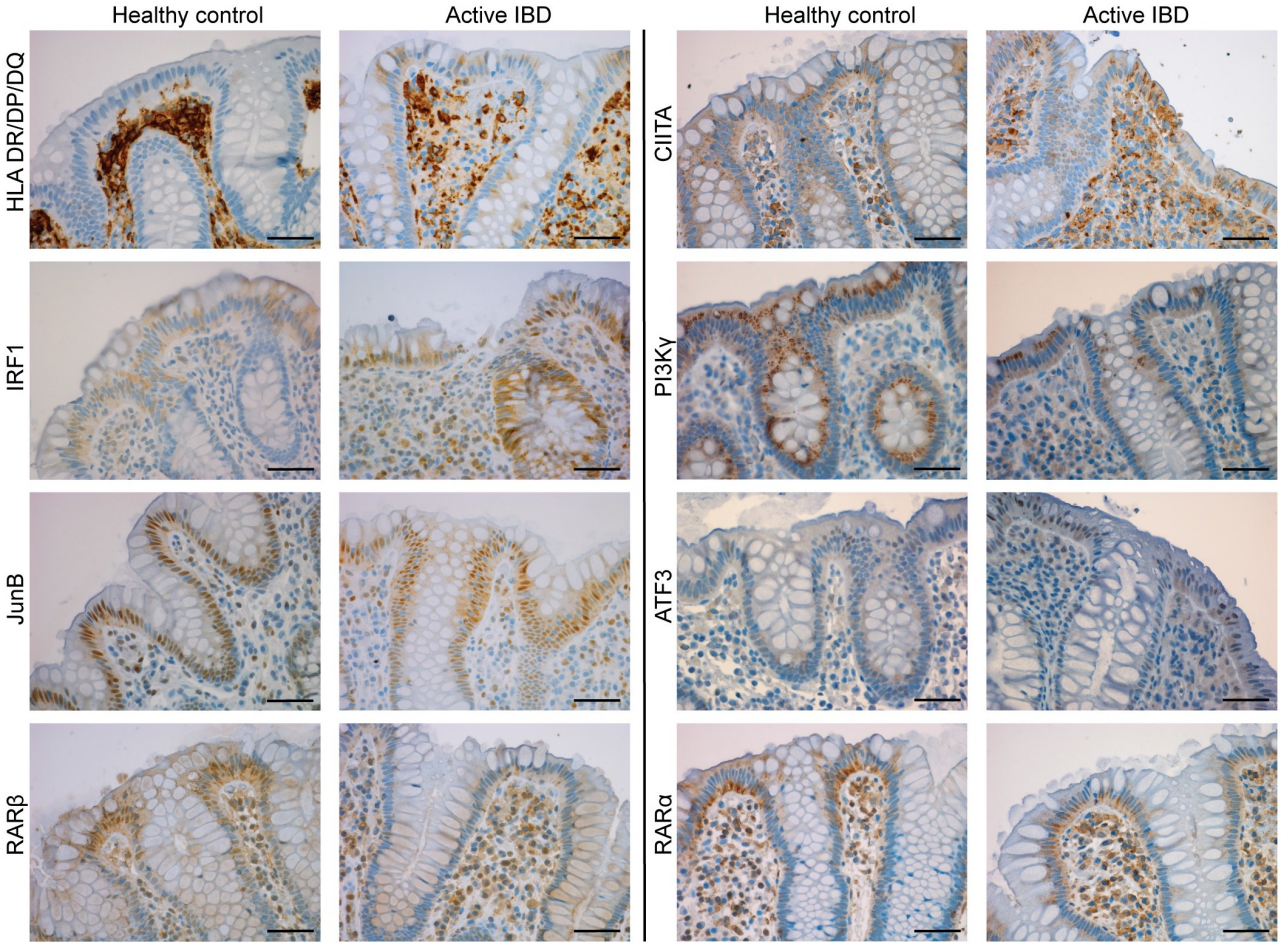
Immunohistochemistry confirms epithelial expression. IHC was used to evaluate the protein expression pattern of a small subset of differentially expressed genes. The proteins evaluated were HLA-DR/DP/DQ, IRF1, JunB, RARβ, CIITA, PI3Kγ, ATF3 and RARα. All staining’s showed positivity in epithelial cells. Scale bars (50μm) indicated.

The main goal of the project was to isolate the epithelial contribution to IBD inflammatory response, and LCM was used to extract the epithelial monolayer from the whole tissue biopsy specimen. IHC of key targets within modules were performed to verify epithelial expression of the encoded proteins. Proteins were selected based on their roles in key pathways identified as differentially regulated in inflamed epithelium. Each antibody was used to stain sections from a minimum of five individuals within each of the patient groups CDa and UCa, as well as healthy controls. Fig 5 shows representative staining of the proteins encoded by the genes *JUNB*, *RARB*, *RARA*, *IRF1*, *HLA-DR/DP/DQ*, *CIITA*, *PIK3CG* and *ATF3*, showing positive staining of the epithelial monolayer for all antibodies. S4 Fig shows the staining at a lower magnification.

### Nuclear ATF3 expression in surface epithelium was significantly increased in UCa vs. HC, but not in CDa vs. HC

**Fig 6:**
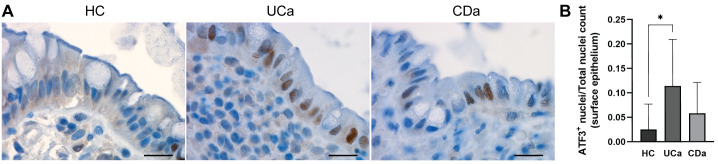
Immunohistochemical ATF3-quantitation confirmed increased nuclear ATF3 positivity in UCa surface epithelium compared to HC. (**A**) Representative ATF3 staining from HC (left), UCa (middle) and CDa (right). Scale bars (20μm) indicated. (**B**) A series of sections from HC (*n* = 12), UCa (*n* = 11) and CDa (*n* = 8) were quantified using QuPath. For each section, surface epithelial ATF3-positivity is presented as the ratio of ATF3^+^ nuclei and total nuclei count. One-way ANOVA followed by Dunnett’s multiple comparisons test showed that nuclear ATF3 positivity was significantly higher in UCa than HC (adj. p = 0.013), but not in CDa vs. HC (adj. p = 0.521).

The genes captured by the module Floralwhite showed a clear distinction in regulation between UC and CD and were thus of interest with regards to characterizing differences in epithelial inflammatory response between the two diseases. A central gene within this module is ATF3, encoding a transcription factor involved in the AP-1 transcriptional complex’s regulation of cellular stress and regeneration. *ATF3* was differentially expressed in the contrast UCa vs. HC (LFC_UCa_ = 3.42, adj. p_UCa_ = 0.011) but not for the contrast CDa vs. HC (LFC_CDa_ = 1.57, adj. p_CDa_ = 0.394). To assess whether this difference in gene expression levels was reflected in protein expression, a series of sections from different individuals within the sample groups HC (*n* = 12), UCa (*n* = 11) and CDa (*n* = 8) were stained for ATF3 (Fig 6A) and quantified using the open source software QuPath. Sections from UCa displayed significantly higher nuclear ATF3 staining in surface epithelium compared to HC (adj. p = 0.013), while CDa sections did not reach significance (adj. p = 0.521, Fig 6B). There was a tendency of sections from both UCa and CDa sections to show more nuclear staining for ATF3 than HC, suggesting nuclear localization of ATF3 in inflamed tissue. To account for the imbalance of steroid-usage between UC and CD patients (see Table 1), only samples from patient not currently undergoing steroid treatment were used in this evaluation. Characteristics of patients and controls included for ATF3-quantitation are given in Table 3.

**Table 3:** Characteristics of IBD patients and controls included for immunohistochemical ATF3-quantitation.

	HC	UCa	CDa
***n***	12	11	8
**Median age (range)**	38.5 (19–70)	33 (17–65)	43.5 (17–71)
**Female sex**	7	6	6
**5-ASA/S-ASA**	0	7	1
**Steroids**	0	0	0

HC, healthy control; UCa, ulcerative colitis with active inflammation; CDa, Crohn’s disease with active inflammation; 5-ASA, 5-aminosalicylic acid; S-ASA, sulphasalazine. Age is given as median, sex and medication as numbers.

## Discussion

Our results present a characterization of the gene expressional changes seen specifically in the colonic epithelium of IBD patients, including both UC and CD. Differential expression analysis contrasting inflamed IBD epithelium with healthy controls identified 3706 differentially expressed genes, of which 1661 were upregulated and 2045 downregulated. In our work, WGCNA was used to stratify genes that correlated across all samples into modules, and further identify the modules where the expression pattern differed between inflamed and healthy control samples. Using the modules in downstream pathway analysis enabled us to identify key genes and processes modulated in the epithelium during active inflammation.

A study from 2018 by Howell et al. has previously presented a whole-genome gene expression analysis of isolated intestinal epithelial cells from IBD patients. However, there are several differences distinguishing this study from the present. Most significant is the difference in study subjects, which were all pediatric patients. While Howell et al. used bead-based live cell sorting to isolate epithelial cells, our gene expression analyses was performed on samples where gene expression was inhibited from the moment of sample collection to RNA isolation. Furthermore, while the focus of Howell et al. was to utilize a multi-omics approach to identify IBD-specific epithelial cell function in pediatric IBD, our focus is purely on characterizing the epithelial inflammatory response in adult IBD on a gene expression level [24].

Limitations in the study are mainly due to inherent imbalances between clinical groups when comparing IBD with HC, which is also apparent in our cohort. The median age is higher in HC than in UC/CD, reflecting the age distribution of healthy individuals undergoing colonoscopy, while 5-ASA/S-ASA usage is higher in UC compared to CD/HC. There is also an imbalance in steroid usage between patient groups, with 3 CD patients receiving steroid treatment at time of sampling, compared to only one within the UC group. In the gene expression analysis, steroid usage was included as a covariate in the regression analysis in order to correct for the possible effect, particularly on the contrast of UCa vs. CDa. For IHC contrasting ATF3 expression in UCa vs. CDa, no patients using steroids were included.

Beyond ATF3, no gene expression differences were verified on a protein level, and further verification would be required in follow-up studies of our initial observations. Indeed, we have previously published several studies where the initial hypotheses were formed on basis of the data presented in full for the first time here, thus representing a thorough validation of parts of the data [14, 25, 26].

There is a distinct over-expression of genes within the MHC Class I/II antigen presentation machinery in the inflamed epithelial monolayer, with the MetaCore pathway “*Immune response_Induction of the antigen presentation machinery by IFN-gamma”* being the single most highly enriched pathway within the data set. MHCII expression is generally restricted to professional antigen presenting cells (e.g. macrophages, dendritic cells and B cells), but can be induced in non-professional antigen presenting cells during inflammation [27]. Our data shows an upregulation of MHC class I and II molecules, key transcription factors/activators (*STAT1*, *IRF1*, *CIITA*), as well as *CD40* in the epithelium of IBD patients with active inflammation. Although enhanced epithelial MHCII expression in IBD has been recognized for decades, the results are fragmented and somewhat contradictory [28]. Most studies have focused on a limited set of genes and/or proteins, and the functional importance is still not fully understood [27]. In the small intestine, epithelial MHCII signalling has been associated with both protective and harmful outcomes, including intestinal stem cell renewal and differentiation [29], and T cell-mediated inflammation during graft-versus-host-disease [30]. It has also been proposed that epithelial cells modulate the immune system indirectly through the release of MHCII-containing exosomes [31], a process shown to be enhanced by IFNγ stimulation [32]. Recently, Bilate et al. described a functional role of epithelial MHCII presentation in relation to intraepithelial lymphocyte differentiation, demonstrating that conditional deletion of either the T cell receptor in CD4^+^ T cells or MHCII in epithelial cells resulted in impaired CD4CD8αα differentiation [33]. Of note, decreased numbers of CD4CD8αα^+^ intraepithelial lymphocytes have been reported in IBD [34]. Though our results reinforce the notion of wide epithelial MHCII expression during active IBD, the exact functional role of this expression is still lacking.

We found a broad epithelial regulation of genes related to Vitamin A-signaling within inflamed samples. The active metabolite of vitamin A, retinoic acid, is an established modulator of the immune system, exerting its main genomic effects by binding heterodimers of retinoic acid receptors (RARs; α/β/γ) and retinoid X receptors (RXRs; α/β/γ) [35]. RA-signaling shapes several aspects of intestinal immunity, including gut-homing of B and T cells [36, 37], IgA class-switch recombination [36], and T cell differentiation [38], and is generally regarded as anti-inflammatory. However, RA-signaling has also been implicated in IBD pathogenesis, where increased capacity of RA-synthesis in macrophages of CD patients correlated with an inflammatory phenotype [39]. Moreover, elevated mucosal RA levels were recently reported in UC patients with active inflammation, where high RA levels correlated with an increase in proinflammatory cytokines [40].

In our analysis of epithelial expression, we found retinoic acid receptors *RARA* (Lightsteelblue), *RARB* (Brown) and *RXRA* (Lightsteelblue) to be downregulated in active IBD. The epithelial response to RA involves both antiproliferative [41–43] and differentiational effects, but also influence the integrity of the epithelial barrier [44]. RA-signaling has been shown to enhance the barrier properties of canine kidney cells (MDCK) through the regulation of tight junction-associated genes [45], and it has been demonstrated that RA affects mucin expression in rats [46]. In addition, RA increased both transepithelial resistance and expression of *TLR4* in Caco-2 cells [47]. Regulation of *RARB* in UC epithelium has previously been observed by Barnicle et al., who found an inverse correlation between methylation status and *RARB* expression in UC epithelium [48]. *RARB* is also found to be downregulated in several types of cancer, including colon cancer [49], and it has been demonstrated that silencing of *RARB* correlates with impaired RARα-signaling [50]. Epithelial downregulation of *RARA* in IBD has not, to our knowledge, previously been reported. Li et al. recently found *Rxra* to be downregulated during DSS-induced colitis [51], and it has been demonstrated that TNFα and IL-1 suppress *RXRA* expression in liver cells [52]. In addition to RARs, RXRs are capable of dimerizing with numerous non-RAR nuclear receptors, including LXRα (*NR1H3*), FXR (*NR1H4*) and PXR (*NR1I2*) [53], who were all found to be downregulated in the epithelial compartment during active IBD.

Interestingly, our data also showed an increased expression of genes encoding retinoid metabolizing enzymes; RDH11, RALDH2/ALDH1A2 and RALDH3/ALDH1A3 (Blue). Retinal dehydrogenases (RALDHs) are considered rate limiting enzymes of RA-synthesis [35]. Smillie et al. also reported an increased expression of ALDH1A2 in UC patients with active inflammation, a regulation unique to enterocytes [19]. This might suggest that IECs contribute to the increased mucosal RA levels observed in IBD patients. This apparent discrepancy between epithelial cells producing more retinoic acid, while at the same time downregulating the expression of RA-responsive receptors is puzzling. Considering previous studies, it might be speculated that epithelial cells contribute to inflammatory cell modulation through increased RA synthesis and secretion, while downregulating the receptor expression to prevent the growth-inhibitory effect on the epithelium during mucosal healing in an RA-saturated milieu. The importance of RA in regulating both the immune system and epithelial barrier integrity makes our findings interesting, in need of further attention.

WGCNA identified the module Floralwhite as UC-specific, which suggested a more prominent AP-1-driven stress response in UC compared to CD. We found the expression of *JUNB* and *ATF3* to be significantly increased in the inflamed epithelium of UC patients, but not in CD. For ATF3, differential expression at the protein level was verified by IHC quantification. JunB and ATF3 are members of the Jun and ATF gene families respectively, which are components of AP-1 transcription factor complex. Dimers of AP-1 proteins regulate a variety of cellular processes (e.g. proliferation, differentiation, and apoptosis), where the effects depend on both the encountered stimuli, cellular state and the AP-1 dimer composition [54]. Both JunB and ATF3 serve important roles within the immune system, including regulation of Th17 effector responses during inflammation [55] and development of follicular helper T cells [56], respectively. Glal et al. have demonstrated a protective role of intestinal Atf3, where *Atf3*^*-/-*^ mice presented with decreased crypt numbers and colon length, and an increased susceptibility to DSS-induced colitis [57]. However, epithelial expression of these transcription factors in IBD patients is less characterized.

Epithelial induction of AP-1 transcription factors has been implicated in various contexts of cellular stress, including oxidative stress, infection and cancer [17]. Human retinal pigment epithelial cells (ARPE-19) were found to upregulate AP-1 transcription factors (including JunB and ATF3) in response to H_2_O_2_ [58], and Sepulveda et al. demonstrated enhanced *JUNB* and *ATF3* expression following *Helicobacter pylori* infection of human gastric epithelial cells (AGS) [59]. *H*. *pylori* infection of gastric mucosal cells (MKN28) have also been shown to enhance the expression of *AREG* [60], another gene clustered into Floralwhite. In addition to *JUNB*, *ATF3 and AREG*, *Salmonella Typhimurium* infection of human epithelial cells (Henle-407) promoted expression of *MCL1*, *RHOB* and *NFKBIA* [61], which are additional members of Floralwhite. Of note, other AP-1 members significantly regulated in UCa but not CDa include *MAFF*, *MAFB and FOS*, all of which clustered into the Blue module. In summary, our results indicate the initiation of a more prominent epithelial stress response involving AP-1 components in UC compared to CD, representing an interesting aspect of IBD heterogeneity that has not previously been acknowledged.

In conclusion, we here present a thorough evaluation of epithelial whole genome gene expression in both colonic UC and CD. Using a combination of unsupervised clustering and enrichment analysis, we identify several novel processes and genes regulated within the epithelial monolayer of IBD patients, contributing to increasing our understanding of epithelial involvement in inflammatory responses. Our data emphasize the role of epithelial cells in expression of immune-modulating genes and proteins and suggests a novel distinction between UC and CD epithelium with regards to AP-1-dependent transcription of stress response genes. We further identify several retinoic acid-associated genes as regulated in inflamed epithelium both UC and CD. Parts of the data have previously been used in organoid-studies of epithelial *ISG15* [26] and *SLC6A4* [14] regulation and signaling in IBD, demonstrating that the data presented in full here can act as a valuable tool for further studies on epithelial involvement in IBD pathophysiology and the contrast of epithelial UC and CD expression, while also confirming *in vivo* relevance of observations from organoid studies.

## Materials and methods

### Clinical material

All subjects were recruited when admitted to St. Olav’s University Hospital for colonoscopy. Healthy control subjects were recruited amongst individuals admitted due to gastroenterological symptoms, but where no significant pathology was found. Inflamed samples were taken from maximally inflamed colonic area with intact epithelial monolayer. Uninflamed samples from IBD patients and healthy controls were all taken from the hepatic flexure. Four closely adjacent biopsies were taken from each area. Two were immediately snap frozen and stored in liquid N_2_ for subsequent laser capture microdissection (LCM) and RNA isolation, while two were formalin fixed (24-72h) and paraffin-embedded for subsequent histology and IHC. Inflammatory status was verified by inspection of hematoxylin and eosin-stained sections and classified into healthy, uninflamed, or active inflammation by an experienced pathologist.

### Laser capture microdissection

For LCM analysis, each sample was handled individually. All room temperature equipment was cleaned using RNAse Zap (Life Technologies, CA, USA), and cryotome with 70% EtOH prior to procedures. Samples were removed from liquid N_2_ while kept inside cooled cryotome chamber, and directly mounted in Tissue-Tek O.C.T. compound embedding medium (Sakura Finetek, CA, USA). The mounted sample was left to equilibrate inside the cryotome for 30 min. Cryosections (10 μm) were made using a Leica CM3050 S (Leica, Wetzlar, Germany) and directly placed on a pre-chilled LCM membrane slide. When the membrane slide was filled with sections, it was briefly exposed to room temperature in order to let sections adhere to the membrane surface, and subsequently placed in 100% EtOH (30 sec). The following staining protocol was modified from the Histogene LCM staining kit (Cat. No. KIT0401, Life Technologies, CA, USA). Sections were washed twice (5 sec) with dH2O with added ProtectRNA RNase inhibitor (Cat. No. R7397, Sigma-Alrich, MO, USA) followed by staining with Histogene staining solution. After subsequent washing steps (3 x 30 sec 100% EtOH, 2 x 2 min Xylene, 1 x 5 min Xylene) sections were air dried (30 sec) and placed in desiccator (30 min). After drying, the membrane slide was processed in an LCM microscope (MMI CellCut on Olympus IX71, Eching, Germany).

### RNA extraction and gene expression analysis

After microdissecting an area of 1 mm^2^ corresponding to approx. 10 000 cells isolated from the whole length of the crypts (see S1 Fig), the sample was lysed by adding 300μl lysis buffer, followed by vortexing (30 sec) and centrifugation (5 sec). The lysed sample was immediately frozen and stored (-80°C) for later RNA isolation. Total RNA was isolated using RNeasy plus micro (cat. No 74034, Qiagen, Venlo, Netherlands) following the manufacturer’s protocol. The quality of isolated RNA was assessed using Bioanalyzer 2100 (Agilent, CA, USA), evaluating the fraction of RNA fragments longer than 200 nt (DV_200_ analysis).

### TruSeq RNA Access sequencing

RNA sequencing libraries were prepared using TruSeq RNA Access library kit (Illumina, Inc., San Diego, CA, USA) according to the manufacturer’s protocol. Initially, total RNA concentration was measured using Qubit^®^ RNA HS Assay Kit on a Qubit^®^ 2.0 Fluorometer (Thermo Fisher Scientific Inc., Waltham, MA, USA). In brief, RNA samples (100 ng total RNA) were fragmented at 94°C for 8 min on a thermal cycler. First strand cDNA syntheses were performed at 25°C for 10 min, 42°C for 15 min and 70°C for 15 min, using random hexamers and SuperScript II Reverse Transcriptase (Thermo Fisher Scientific Inc., Waltham, MA, USA). In a second strand cDNA synthesis the RNA templates were removed, and a second replacement strand was generated by incorporation dUTP (in place of dTTP, to keep strand information) to generate double-stranded cDNA. AMPure XP beads (Beckman Coulter, Inc., Indianapolis, IN, USA) were used to clean up the blunt-ended cDNA from the second strand reaction mix. The 3’ends of the cDNA were then adenylated to facilitate adaptor ligation in the next step. After ligation of indexing adaptors, AMPure XP beads were used to clean up the libraries. In a first PCR amplification step, PCR (15 cycles of 98°C for 10 sec, 60°C for 30 sec and 72°C for 30 sec) was used to selectively enrich those DNA fragments that had adapter molecules on both ends and to amplify the amount of DNA in the library. The libraries were then validated using Agilent DNA 1000 kit on a 2100 Bioanalyzer instrument. The final products were approximately 260 bp. After validation of the libraries the first hybridization step was performed using exome capture probes. Prior to hybridization, a 4-plex pool of libraries was made by combining 200 ng of each DNA library. The hybridization was performed by 18 cycles of one minute incubation, starting at 94°C, and then decreasing 2°C per cycle. Streptavidin-coated magnetic beads were used to capture probes hybridized to the target regions. The enriched libraries were then eluted from the beads and prepared for a second round of hybridization. This second hybridization (18 cycles of one minute incubation, starting at 94°C, and then decreasing 2°C per cycle) was required to ensure high specificity of the capture regions. A second capture with streptavidin-coated beads was performed, followed by two heated wash procedures to remove non-specific binding from the beads. The enriched libraries where then eluted from the beads and cleaned up by AMPure XP beads prior to a second PCR amplification. The amplification step was performed by 10 cycles (98°C for 10 sec, 60°C for 30 sec and 72°C for 30 sec) followed by a second PCR clean up using AMPure XP beads. Finally, the libraries were quantitated by qPCR using KAPA Library Quantification Kit—Illumina/ABI Prism^®^ (Kapa Biosystems, Inc., Wilmington, MA, USA) and validated using Agilent High Sensitivity DNA Kit on a Bioanalyzer. The size range of the DNA fragments was measured to be in the range of 200–650 bp and peaked around 270 bp. Libraries were normalized to 22 pM and subjected to cluster and single read sequencing was performed for 50 cycles on a HiSeq2500 instrument (Illumina, Inc. San Diego, CA, USA), according to the manufacturer’s instructions. Base calling was done on the HiSeq instrument by RTA 1.17.21.3. FASTQ files were generated using CASAVA 1.8.2 (Illumina, Inc. San Diego, CA, USA).

### Enrichment analysis

Genes that were differentially expressed between IBDa and HC were explored using Gene Set Enrichment Analysis (GSEA v4.1.0 build 27) on transformed gene expression counts. Gene sets were derived from single-cell sequencing studies of colonic epithelium, focusing on identifying changes in differentiation as a response to inflammation [7, 19]. Of 30 gene sets, only the set derived from Parhik et al. defining enteroendocrine cells was differentially enriched in IBDa. Genes differentially expressed in the contrast IBDa vs. HC, and genes within each WGCNA module significantly correlated with active IBD were uploaded to MetaCore to determine top enriched pathways and GO processes. To avoid broad, less informative GO terms (e.g. *Cellular process*) a cut-off at < 1500 genes per process was set. For exploration of the global regulation in active IBD, the total list of differentially expressed genes (IBDa vs. HC) was analyzed in MetaCore using the same approach as described above.

### Immunohistochemistry

Formalin-fixed paraffin-embedded tissue sections (4μm) were de-paraffinized in Neo-Clear^®^ and rehydrated through an ethanol gradient (100 through 70%), followed by rinsing in dH_2_O. Blocking of endogenous peroxidases was done in 3% H_2_O_2_ (10 min) prior to heat-induced epitope-retrieval. Depending on the antibody, heat-induced epitope-retrieval was performed using either citrate buffer (pH 6, 15 min) or Tris-EDTA buffer (pH 9, 15 min). Primary antibody incubations were done overnight at 4°C, using TBS with 0.05% Tween20 and 1% BSA as antibody-diluent, in exception for anti-JunB which was incubated for 1h at room temperature. Further details about antibodies are provided in Table 4. Visualization was performed using the Rabbit/Mouse Dako REAL EnVision Detection System (K500711-2, Dako, Glostrup, Denmark). Hematoxylin was used as counterstain.

**Table 4:** Description of primary antibodies and immunohistochemistry parameters.

Target	Cat #	Commercial supplier	Species/clonality	Dilution	Buffer pH
CIITA	ab117598	Abcam, Cambridge, UK	Rabbit polyclonal	1:1500	9
IRF1	ab186384	Abcam, Cambridge, UK	Rabbit monoclonal	1:1000	9
HLA-DR/DP/DQ	ab17101	Abcam, Cambridge, UK	Mouse monoclonal	1:200	9
RARβ	HPA004174	Prestige antibodies^®^ Sigma-Aldrich, St. Louis, USA	Rabbit polyclonal	1:500	6
RARα	H00005914-M01	Abnova, Taipei, Taiwan	Mouse monoclonal	1:1600	6
ATF3	MA5-31360	Invitrogen, Carlsbad, USA	Mouse monoclonal	1:100	9
PI3Kγ	TA505221	Origene, Rockville, USA	Mouse monoclonal	1:200	9
JunB	HPA019149	Prestige antibodies^®^ Sigma-Aldrich, St. Louis, USA	Rabbit polyclonal	1:400	6

### Quantitation of nuclear ATF3 expression in surface epithelium

Sections were scanned in an Olympus VS120-S5 slide scanner using a 20x objective. The total area of surface epithelium within each section was manually annotated, and subsequently counted using the built-in *Positive cell detection*-analysis in QuPath v0.3.0 [62]. For each section, nuclear ATF3-positivity is presented as the ratio of positive nuclei and total nuclei count.

### Statistical analysis

Gene expression analysis and WGCNA was done in the R software for statistical computing [63]. Sets of DEGs were identified using LIMMA linear models with least squares regression and empirical bayes moderated t-statistics [64]. Steroid treatment status was included as a covariate. Significance measures were corrected for multiple testing using Benjamini–Hochberg FDR-adjusted p-values. WGCNA was used for unsupervised clustering of genes with highly correlated expression patterns across samples, independent of disease status [65]. Prior to analysis, counts were transformed using variance stabilizing transformation in DeSEQ2 [66]. WGCNA was performed according to recommendations given by Langfelder and Horvath in the WGCNA package documentation. Specifically, soft threshold of 9 was used when calculating the signed hybrid network. Minimal module size was initially set to 30 genes, with modules subsequently merged using a distance threshold of 0.25, to a final set of 10 modules. Six modules showed a significant module-trait relationship with IBDa, and a list of genes for each module was exported for subsequent gene set enrichment analysis. Results (LFC and adj. p) from LIMMA analysis were divided into modules as identified by WGCNA and used in enrichment analyses in MetaCore version 20.4 build 70300. For evaluation of nuclear ATF3 positivity, one-way ANOVA followed by Dunnett’s multiple comparisons test was performed in GraphPad Prism 8.0.

### Study approval

The study was approved by the Regional Committee for Medical and Health Research Ethics (reference numbers 5.2007.910 and 2013/212/REKmidt) and was registered in the Clinical Trials Protocol Registration System (identifier NCT00516776). All subjects gave informed, written consent prior to inclusion.

## Supporting information

S1 Fig Isolation of the epithelial monolayer using laser capture microdissection. Laser capture microdissection was used to isolate the epithelial monolayer from inflammatory bowel disease patients and healthy controls. An area corresponding to approximately 10 000 cells (1 mill μm^2^) was isolated from each individual (healthy control = 6, active ulcerative colitis = 7, active Crohn’s disease = 5, uninflamed ulcerative colitis = 6, uninflamed Crohn’s disease = 5). Isolated areas were collected and used in subsequent RNA isolation.
(TIF)

S2 Fig Top 10 up- and downregulated pathways in IBDa vs. HC. Top 10 upregulated (left) and downregulated (right) pathways in IBDa vs. HC. Figures exported from MetaCore.
(TIF)

S3 Fig Correlation of module membership score vs. gene significance in IBDa vs. HC. A correlation plot for the degree of significance for gene expression difference in the IBDa vs HC contrast and module membership. High correlation suggests that the module captures and emphasizes genes whose gene expression is differentially expressed in the contrast.
(TIF)

S4 Fig Immunohistochemistry confirms epithelial expression of key proteins. Immunohistochemistry showing larger parts of sections from healthy controls and active inflammatory bowel disease samples, confirming the epithelial expression of key proteins. Immunohistochemistry was used to evaluate whether epithelial cells express the proteins encoded by a small subset of differentially expressed genes. The proteins evaluated were HLA-DR/DP/DQ, IRF1, JunB, RARβ, CIITA, PI3Kγ, ATF3 and RARα. All staining’s showed positivity in epithelial cells. Scale bars (100μm) indicated.
(TIF)

S1 File (XLSX)

S2 File (XLSX)

S3 File (DOCX)
