# Correction: Profound gene expression changes in the epithelial monolayer of active ulcerative colitis and Crohn’s disease

**DOI:** 10.1371/journal.pone.0325481

**Published:** 2025-05-27

**Authors:** Siri Sæterstad, Ann Elisabet Østvik, Elin Synnøve Røyset, Ingunn Bakke, Arne Kristian Sandvik, Atle van Beelen Granlund

In the Author Contributions, Siri Sæterstad should be credited for Visualization.

The Data Availability statement for this article is incorrect. The correct statement is: All analysis results are included as Supporting Information, RNA‐Seq counts not included within the paper are made available through NCBI’s Gene Expression Omnibus (GEO) DataSet GSE179128.
