# Supplementary figures and images for: Profound gene expression changes in the epithelial monolayer of active ulcerative colitis and Crohn’s disease
Source: PLoS One. 2022 Mar 11;17(3):e0265189. doi: 10.1371/journal.pone.0265189 (PMC8916644; doi:10.1371/journal.pone.0265189)

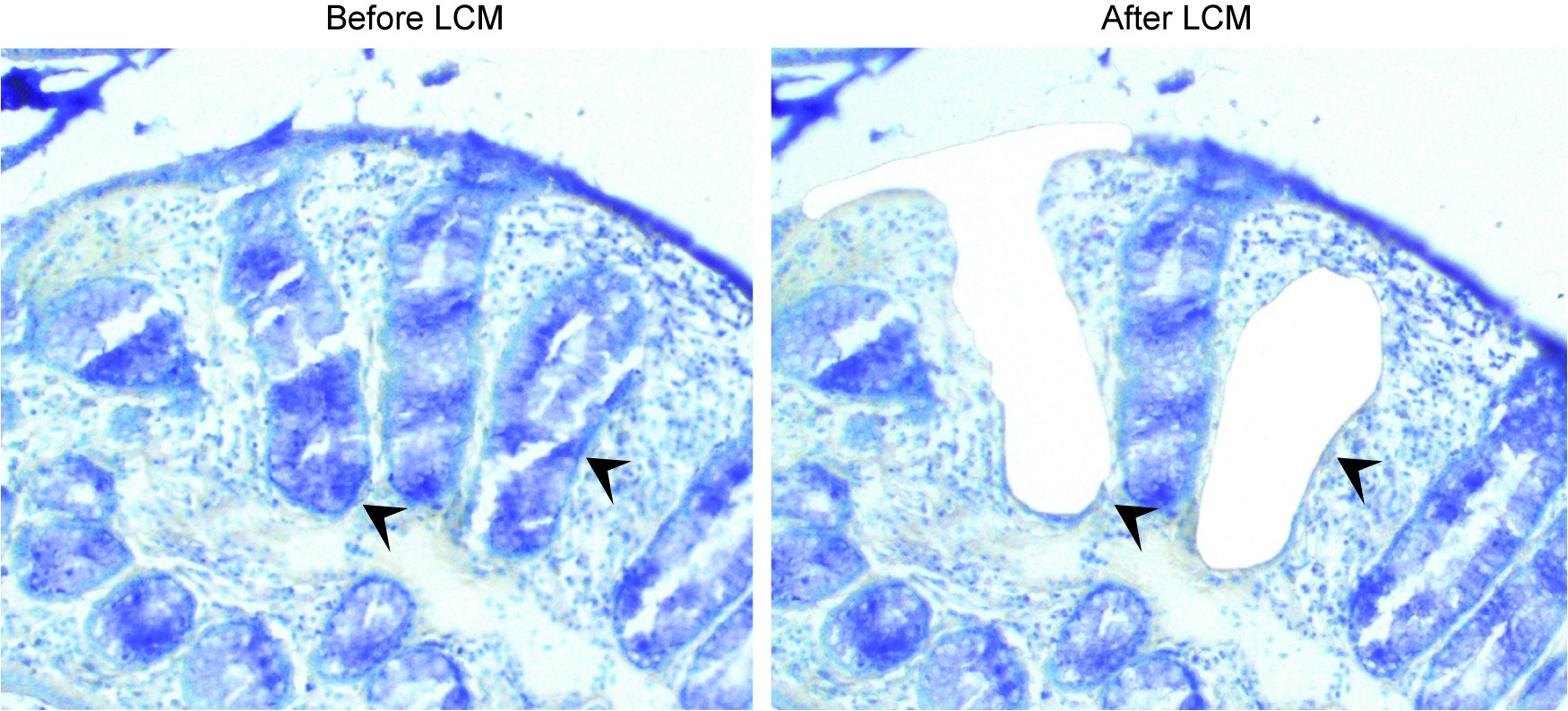

Supplement: S1 Fig — Laser capture microdissection was used to isolate the epithelial monolayer from inflammatory bowel disease patients and healthy controls. An area corresponding to approximately 10 000 cells (1 mill μm2) was isolated from each individual (healthy control = 6, active ulcerative colitis = 7, active Crohn’s disease = 5, uninflamed ulcerative colitis = 6, uninflamed Crohn’s disease = 5). Isolated areas were collected and used in subsequent RNA isolation. (TIF) [file pone.0265189.s001.tif]

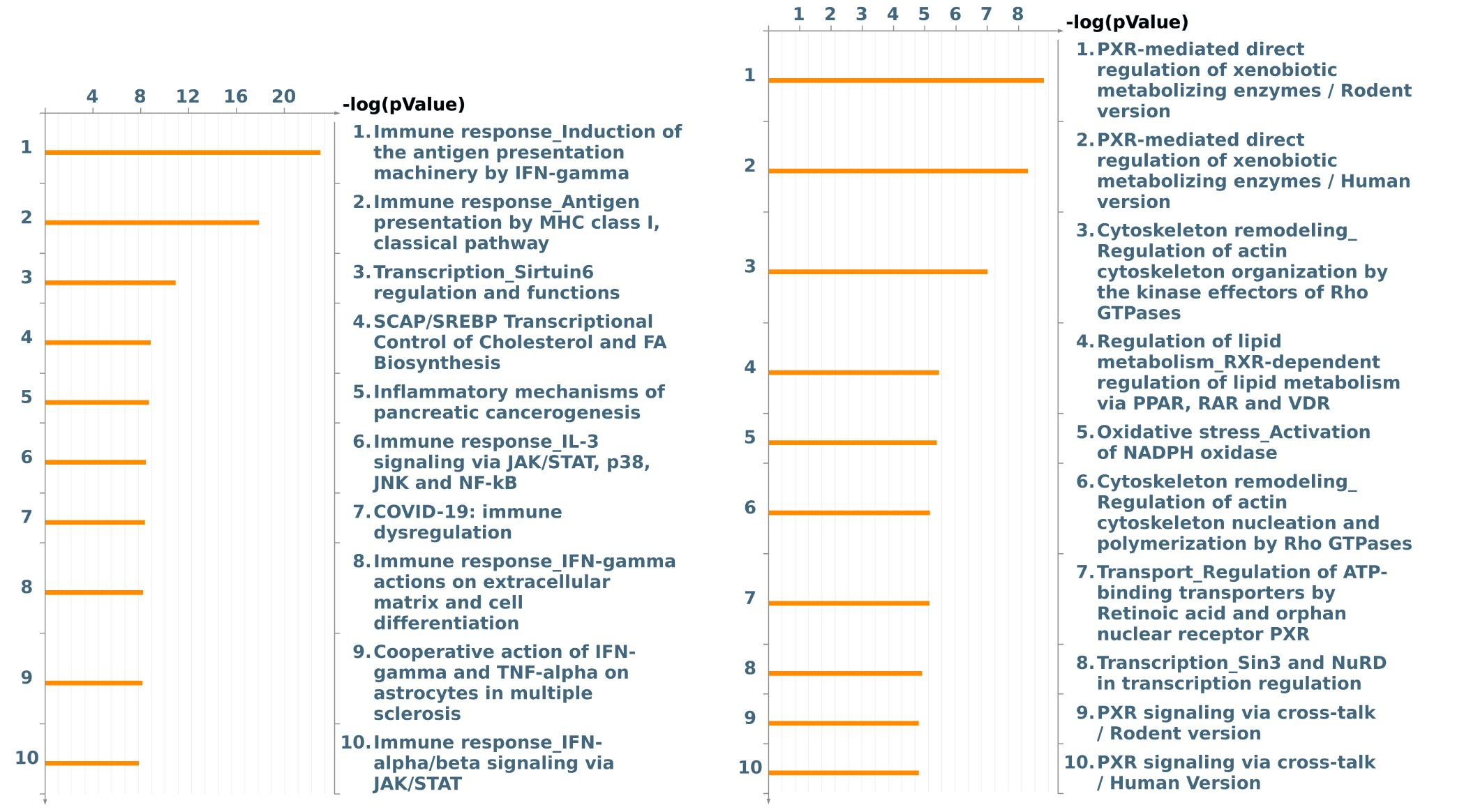

Supplement: S2 Fig — Top 10 upregulated (left) and downregulated (right) pathways in IBDa vs. HC. Figures exported from MetaCore. (TIF) [file pone.0265189.s002.tif]

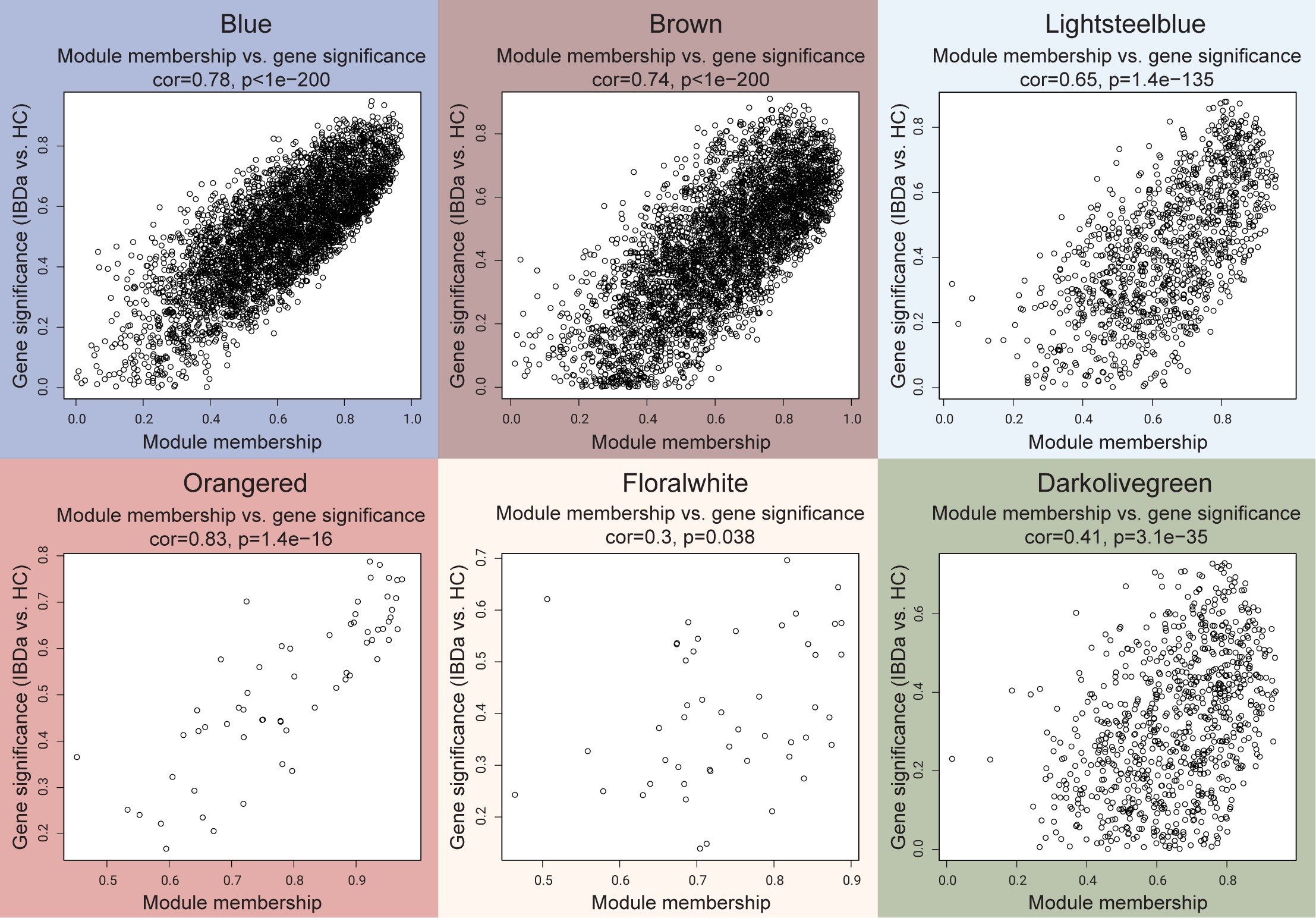

Supplement: S3 Fig — A correlation plot for the degree of significance for gene expression difference in the IBDa vs HC contrast and module membership. High correlation suggests that the module captures and emphasizes genes whose gene expression is differentially expressed in the contrast. (TIF) [file pone.0265189.s003.tif]

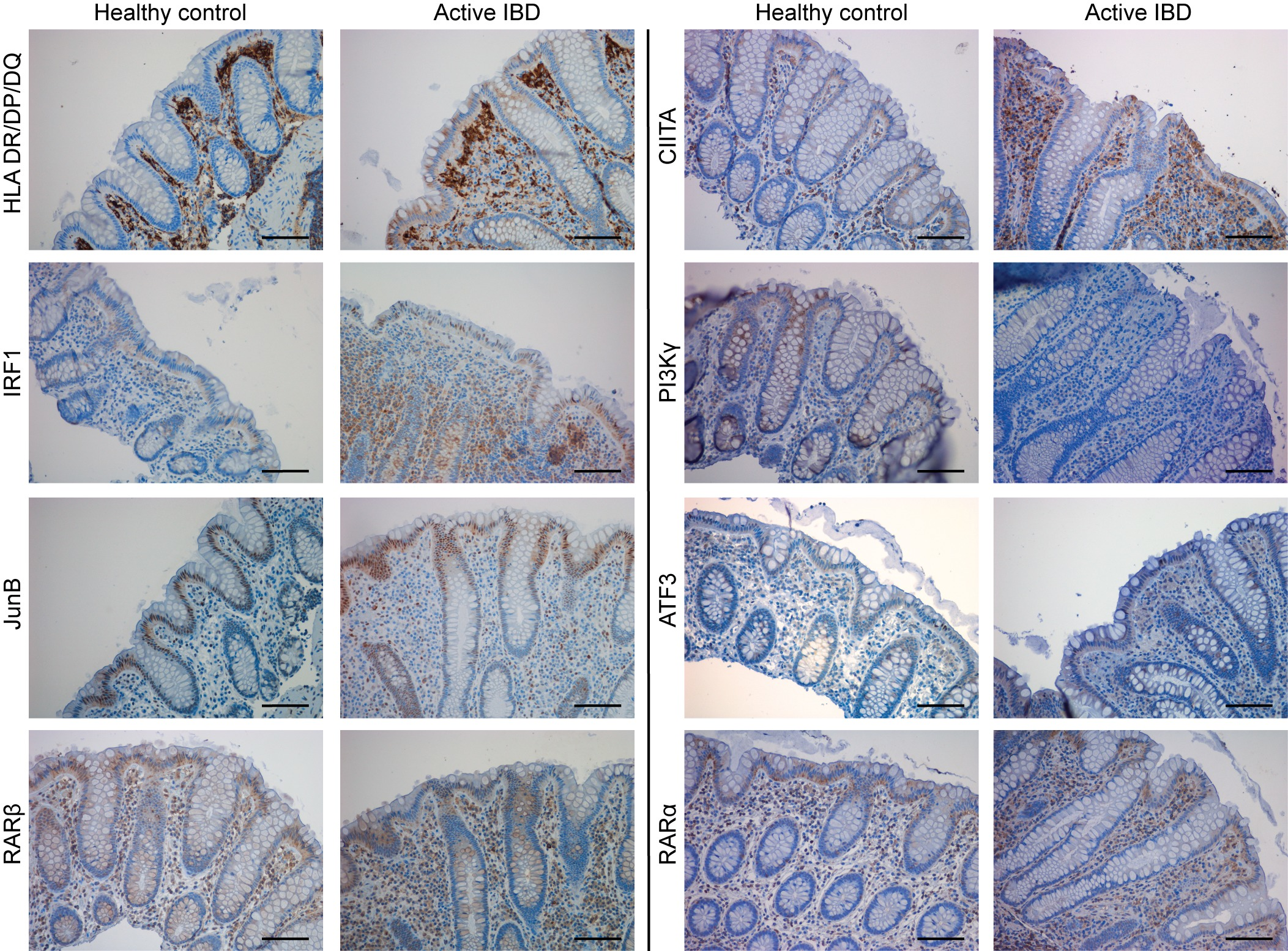

Supplement: S4 Fig — Immunohistochemistry showing larger parts of sections from healthy controls and active inflammatory bowel disease samples, confirming the epithelial expression of key proteins. Immunohistochemistry was used to evaluate whether epithelial cells express the proteins encoded by a small subset of differentially expressed genes. The proteins evaluated were HLA-DR/DP/DQ, IRF1, JunB, RARβ, CIITA, PI3Kγ, ATF3 and RARα. All staining’s showed positivity in epithelial cells. Scale bars (100μm) indicated. (TIF) [file pone.0265189.s004.tif]
