# Supplementary material for: Profound gene expression changes in the epithelial monolayer of active ulcerative colitis and Crohn’s disease
Source: PLoS One. 2022 Mar 11;17(3):e0265189. doi: 10.1371/journal.pone.0265189 (PMC8916644; doi:10.1371/journal.pone.0265189)
Supplement: S3 File — (DOCX) [file pone.0265189.s007.docx]

Contents

[IBDa vs. HC 3](#_Toc89285726)

[Table of content: 5](#_Toc89285727)

[Enrichment analysis (TOC) 6](#_Toc89285728)

[Pathway Maps (TOC) 7](#_Toc89285729)

[Top maps (sorted by Statistically significant Maps) 8](#_Toc89285730)

[Process Networks (TOC) 13](#_Toc89285731)

[Diseases (by Biomarkers) (TOC) 14](#_Toc89285732)

[GO Processes (TOC) 15](#_Toc89285733)

[Blue 16](#_Toc89285734)

[Table of content: 17](#_Toc89285735)

[Enrichment analysis (TOC) 18](#_Toc89285736)

[Pathway Maps (TOC) 19](#_Toc89285737)

[Top maps (sorted by Statistically significant Maps) 20](#_Toc89285738)

[Process Networks (TOC) 25](#_Toc89285739)

[Diseases (by Biomarkers) (TOC) 26](#_Toc89285740)

[GO Processes (TOC) 27](#_Toc89285741)

[Brown 28](#_Toc89285742)

[Table of content: 29](#_Toc89285743)

[Enrichment analysis (TOC) 30](#_Toc89285744)

[Pathway Maps (TOC) 31](#_Toc89285745)

[Top maps (sorted by Statistically significant Maps) 32](#_Toc89285746)

[Process Networks (TOC) 37](#_Toc89285747)

[Diseases (by Biomarkers) (TOC) 38](#_Toc89285748)

[GO Processes (TOC) 39](#_Toc89285749)

[Darkolivegreen 40](#_Toc89285750)

[Table of content: 41](#_Toc89285751)

[Enrichment analysis (TOC) 42](#_Toc89285752)

[Pathway Maps (TOC) 43](#_Toc89285753)

[Top maps (sorted by Statistically significant Maps) 44](#_Toc89285754)

[Process Networks (TOC) 49](#_Toc89285755)

[Diseases (by Biomarkers) (TOC) 50](#_Toc89285756)

[GO Processes (TOC) 51](#_Toc89285757)

[Floralwhite 52](#_Toc89285758)

[Table of content: 53](#_Toc89285759)

[Enrichment analysis (TOC) 54](#_Toc89285760)

[Pathway Maps (TOC) 55](#_Toc89285761)

[Top maps (sorted by Statistically significant Maps) 56](#_Toc89285762)

[Process Networks (TOC) 61](#_Toc89285763)

[Diseases (by Biomarkers) (TOC) 62](#_Toc89285764)

[GO Processes (TOC) 63](#_Toc89285765)

[Lightsteelblue 64](#_Toc89285766)

[Table of content: 65](#_Toc89285767)

[Enrichment analysis (TOC) 66](#_Toc89285768)

[Pathway Maps (TOC) 67](#_Toc89285769)

[Top maps (sorted by Statistically significant Maps) 68](#_Toc89285770)

[Process Networks (TOC) 73](#_Toc89285771)

[Diseases (by Biomarkers) (TOC) 74](#_Toc89285772)

[GO Processes (TOC) 75](#_Toc89285773)

[Orangered 76](#_Toc89285774)

[Table of content: 77](#_Toc89285775)

[Enrichment analysis (TOC) 78](#_Toc89285776)

[Pathway Maps (TOC) 79](#_Toc89285777)

[Top maps (sorted by Statistically significant Maps) 80](#_Toc89285778)

[Process Networks (TOC) 85](#_Toc89285779)

[Diseases (by Biomarkers) (TOC) 86](#_Toc89285780)

[GO Processes (TOC) 87](#_Toc89285781)

IBDa vs. HC

**Enrichment Analysis Workflow 1.0 Data Analysis Report**

*Server: portal.genego.com*

*Date: 2021‑12‑01*

*Name: NORWEGIAN UNIV OF SCI and TECH | Arnar Flatberg | arnar.flatberg@ntnu.no*

*Login: ntnu3*

Experiments

| 1. |  | IBDa vs HC |
| --- | --- | --- |

The experiments uploaded for comparative analysis

## Table of content:

**[•](#Bookmark_1)** [Enrichment analysis](#Bookmark_1)

**[•](#Bookmark_2)** [Pathway Maps](#Bookmark_2)

**[•](#Bookmark_3)** [Top maps (sorted by Statistically significant Maps)](#Bookmark_3)

**[•](#Bookmark_4)** [1. Map : Immune response_Induction of the antigen presentation machinery by IFN‑gamma](#Bookmark_4)

**[•](#Bookmark_5)** [2. Map : Immune response_Antigen presentation by MHC class I, classical pathway](#Bookmark_5)

**[•](#Bookmark_6)** [3. Map : Transcription_Sirtuin6 regulation and functions](#Bookmark_6)

**[•](#Bookmark_7)** [4. Map : Immune response_IL‑3 signaling via JAK/STAT, p38, JNK and NF‑kB](#Bookmark_7)

**[•](#Bookmark_8)** [5. Map : Cooperative action of IFN‑gamma and TNF‑alpha on astrocytes in multiple sclerosis](#Bookmark_8)

**[•](#Bookmark_9)** [Process Networks](#Bookmark_9)

**[•](#Bookmark_10)** [Diseases (by Biomarkers)](#Bookmark_10)

**[•](#Bookmark_11)** [GO Processes](#Bookmark_11)

## Enrichment analysis ([TOC](#TOC_table))

Enrichment analysis consists of matching gene IDs of possible targets for the "common", "similar" and "unique" sets with gene IDs in functional ontologies in MetaCore. The probability of a random intersection between a set of IDs the size of target list with ontology entities is estimated in p‑value of hypergeometric intersection. The lower p‑value means higher relevance of the entity to the dataset, which shows in higher rating for the entity.

Ontologies available for EA in Enrichment Analysis Workflow:

### Pathway Maps ([TOC](#TOC_table))

Canonical pathway maps represent a set of signaling and metabolic maps covering human in a comprehensive way. All maps are created by Clarivate scientists by a high‑quality manual curation process based on published peer‑reviewed literature. Experimental data is visualized on the maps as blue (for downregulation) and red (upregulation) histograms. The height of the histogram corresponds to the relative expression value for a particular gene/protein.


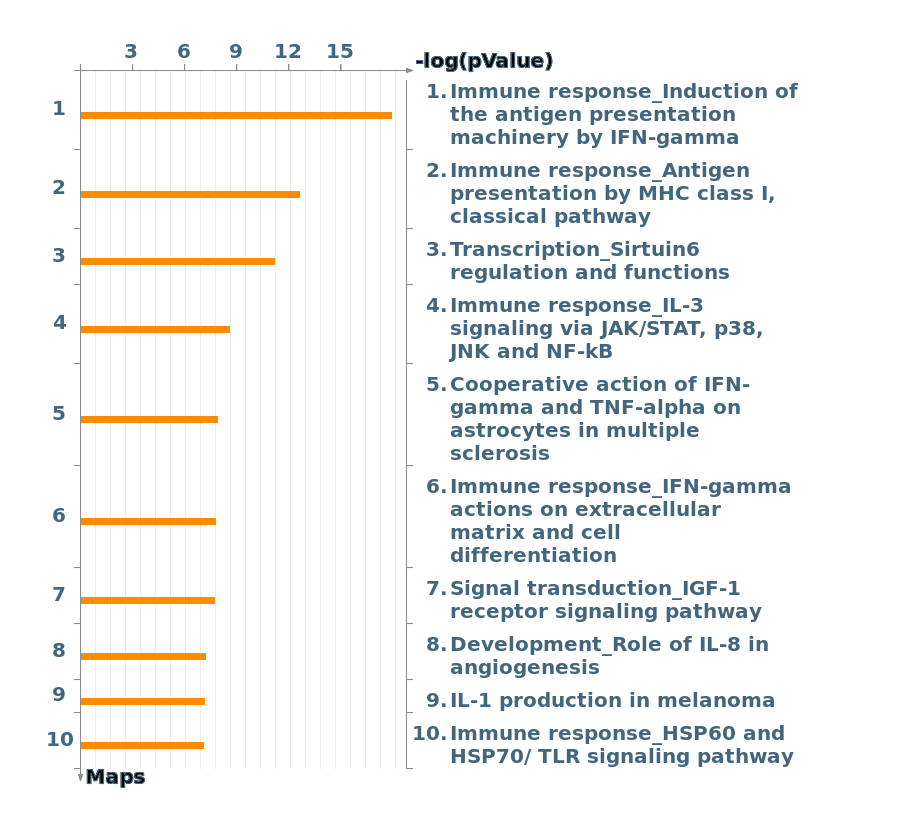


**Figure 2.** Pathway Maps. Sorting is done for the 'Statistically significant Maps'.

### Top maps (sorted by Statistically significant Maps)

**1. Map :** [Immune response_Induction of the antigen presentation machinery by IFN‑gamma](https://portal.genego.com/cgi/imagemap.cgi?id=2647)
([TOC](#TOC_table))


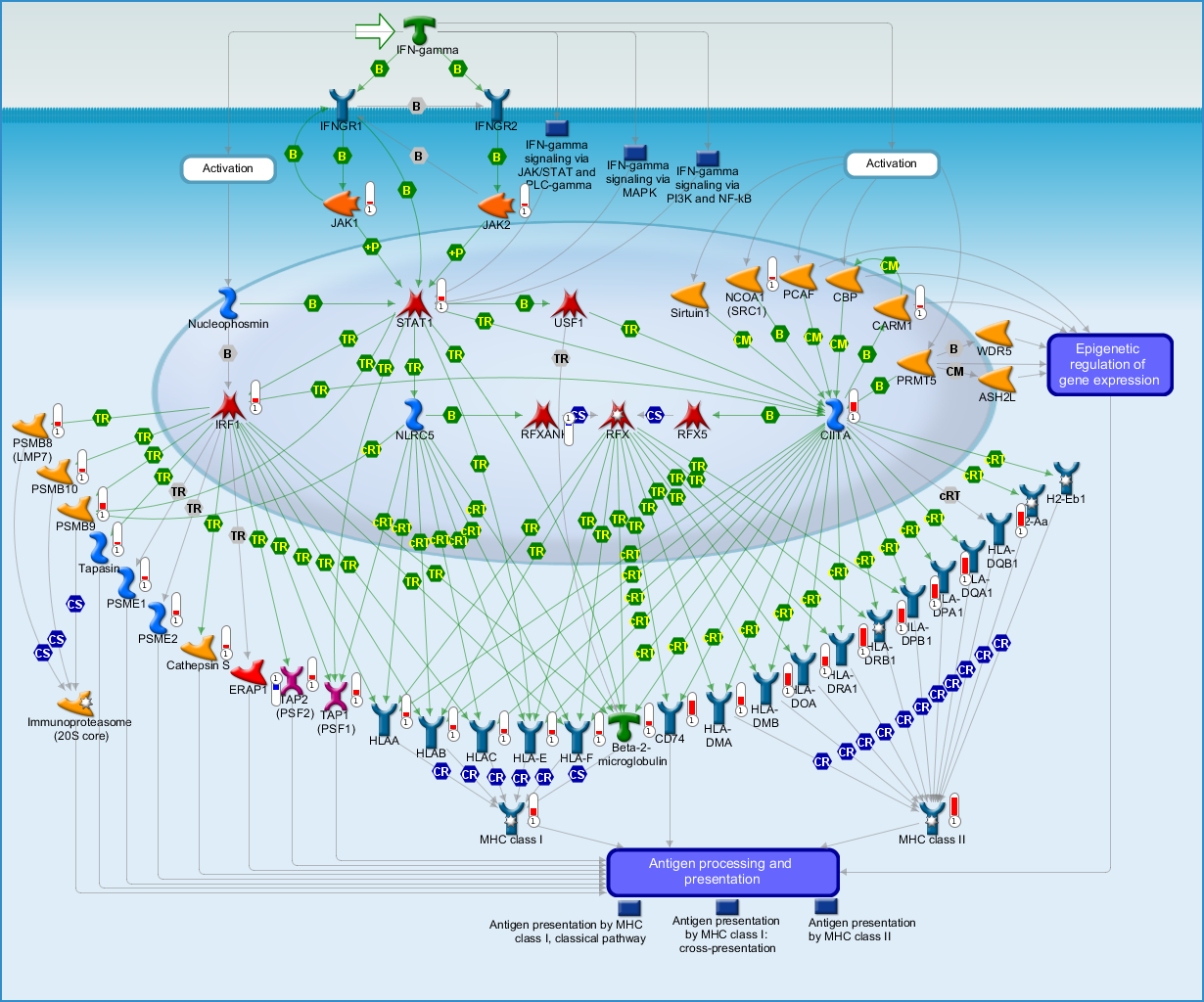


**Figure 3.** The top scored map (map with the the lowest p‑value) based on the enrichment distribution sorted by 'Statistically significant Maps' set. Experimental data from all files is linked to and visualized on the maps as thermometer‑like figures. Up‑ward thermometers have red color and indicate up‑regulated signals and down‑ward (blue) ones indicate down‑regulated expression levels of the genes.

**2. Map :** [Immune response_Antigen presentation by MHC class I, classical pathway](https://portal.genego.com/cgi/imagemap.cgi?id=2100)
([TOC](#TOC_table))


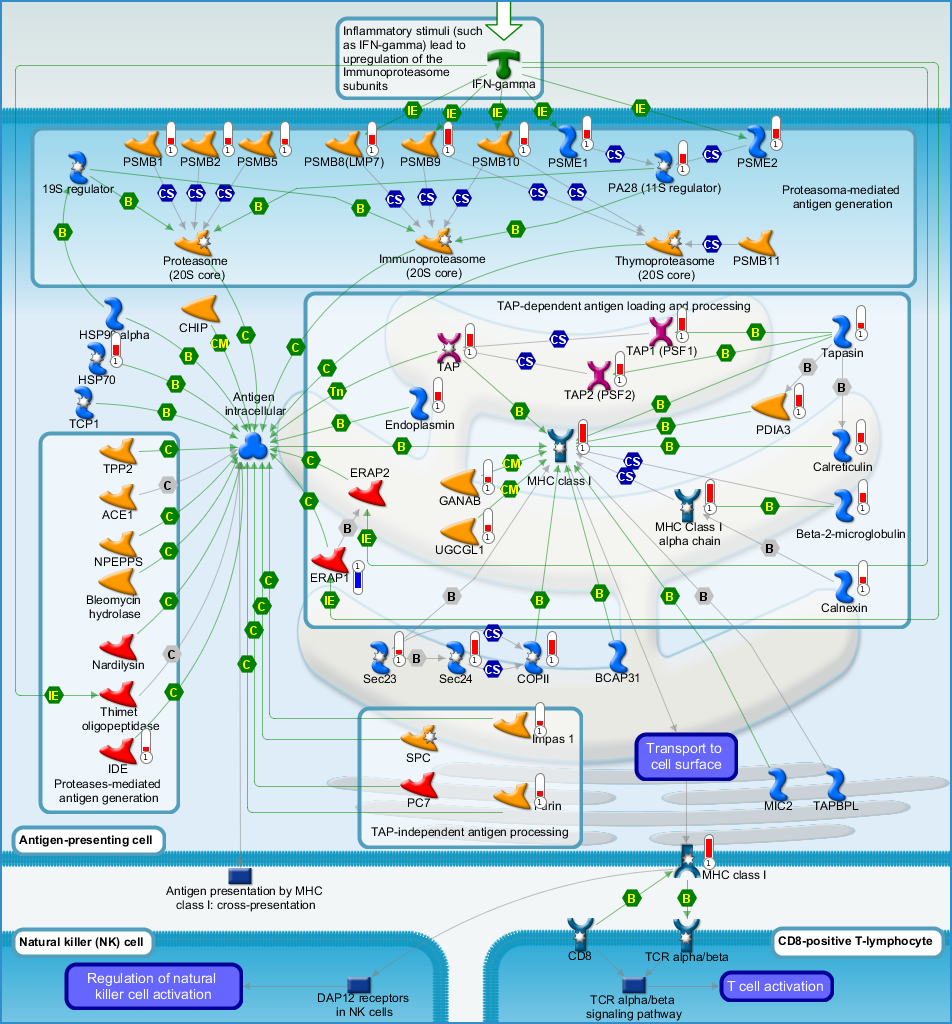


**Figure 4.** The second scored map (map with the second lowest p‑value) based on the enrichment distribution sorted by 'Statistically significant Maps' set. Experimental data from all files is linked to and visualized on the maps as thermometer‑like figures. Up‑ward thermometers have red color and indicate up‑regulated signals and down‑ward (blue) ones indicate down‑regulated expression levels of the genes.

**3. Map :** [Transcription_Sirtuin6 regulation and functions](https://portal.genego.com/cgi/imagemap.cgi?id=6935)
([TOC](#TOC_table))


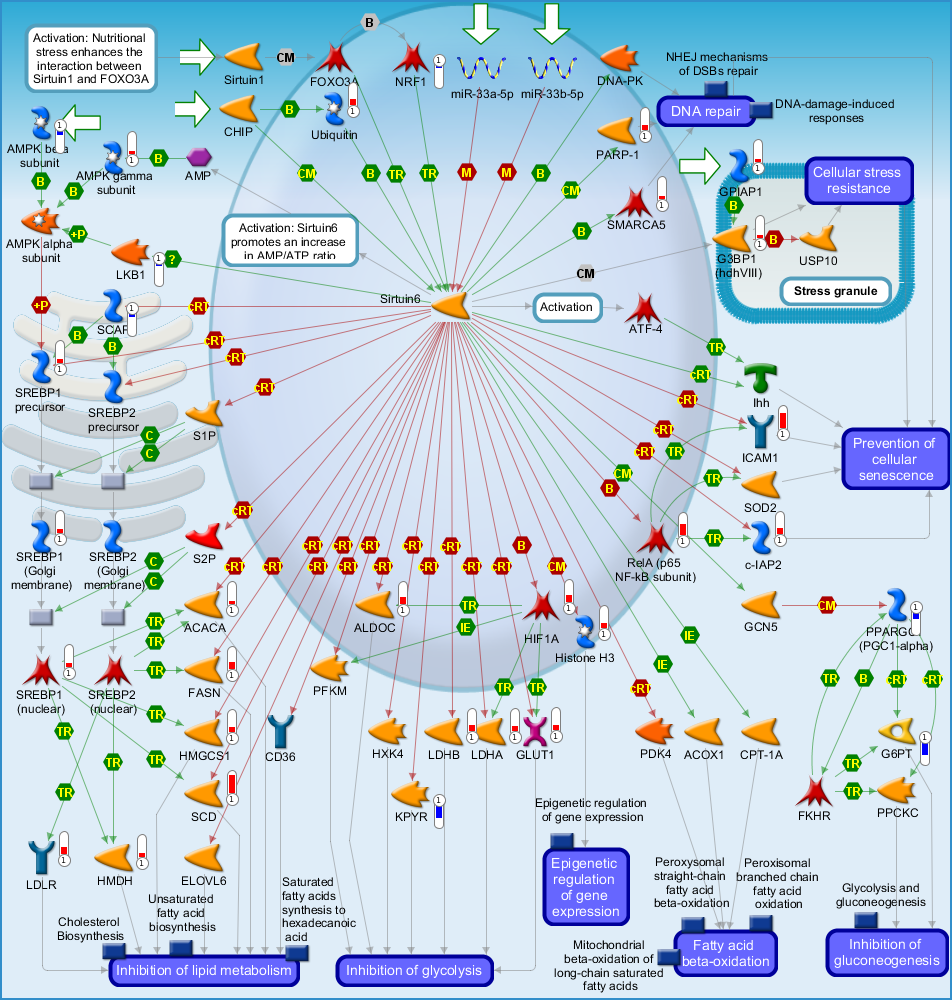


**Figure 5.** The third scored map (map with the third lowest p‑value) based on the enrichment distribution sorted by 'Statistically significant Maps' set. Experimental data from all files is linked to and visualized on the maps as thermometer‑like figures. Up‑ward thermometers have red color and indicate up‑regulated signals and down‑ward (blue) ones indicate down‑regulated expression levels of the genes.

**4. Map :** [Immune response_IL‑3 signaling via JAK/STAT, p38, JNK and NF‑kB](https://portal.genego.com/cgi/imagemap.cgi?id=657)
([TOC](#TOC_table))


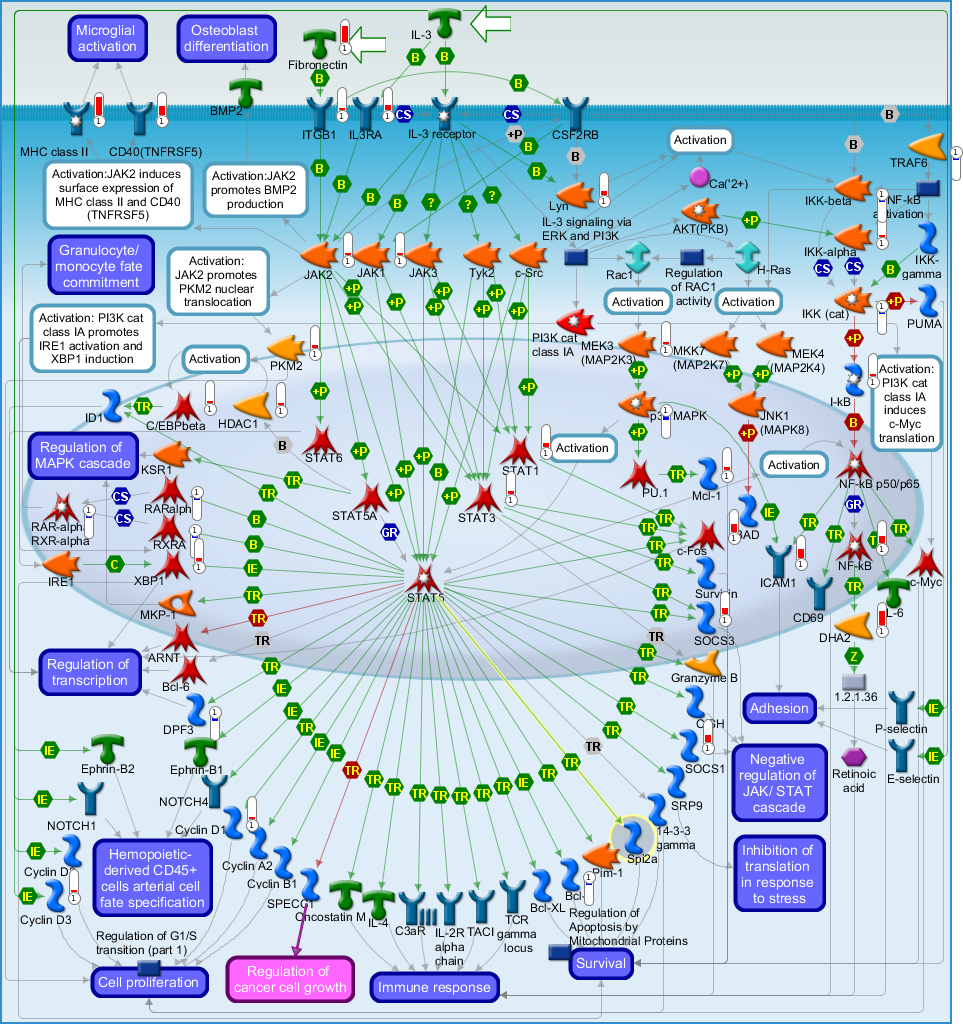


**Figure 6.** The fourth scored map (map with the fourth lowest p‑value) based on the enrichment distribution sorted by 'Statistically significant Maps' set. Experimental data from all files is linked to and visualized on the maps as thermometer‑like figures. Up‑ward thermometers have red color and indicate up‑regulated signals and down‑ward (blue) ones indicate down‑regulated expression levels of the genes.

**5. Map :** [Cooperative action of IFN‑gamma and TNF‑alpha on astrocytes in multiple sclerosis](https://portal.genego.com/cgi/imagemap.cgi?id=5199)
([TOC](#TOC_table))


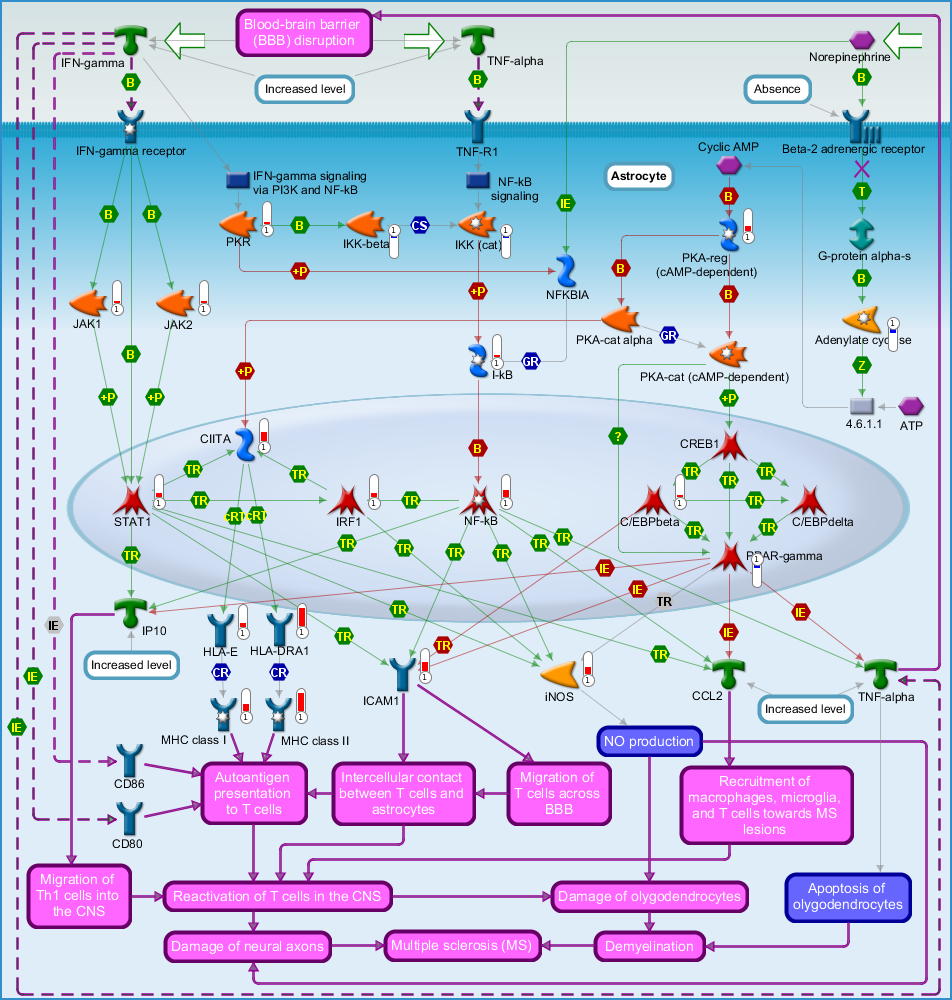


**Figure 7.** The fifth scored map (map with the fifth lowest p‑value) based on the enrichment distribution sorted by 'Statistically significant Maps' set. Experimental data from all files is linked to and visualized on the maps as thermometer‑like figures. Up‑ward thermometers have red color and indicate up‑regulated signals and down‑ward (blue) ones indicate down‑regulated expression levels of the genes.

### Process Networks ([TOC](#TOC_table))

The content of these cellular and molecular processes is defined and annotated by Clarivate scientists. Each process represents a pre‑set network of protein interactions characteristic for the process.


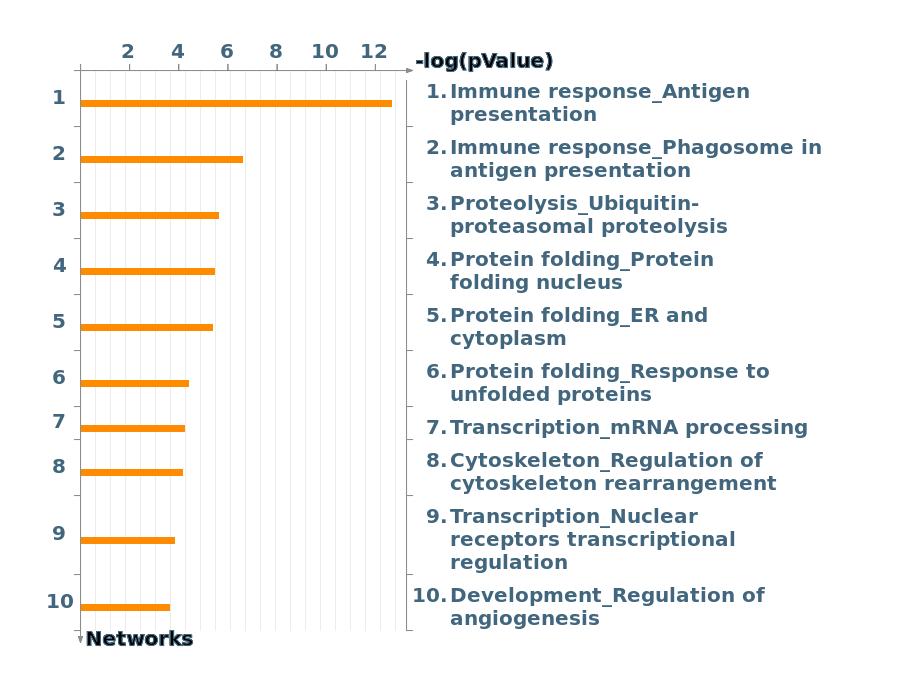


**Figure 8.** Process Networks. Sorting is done for the 'Statistically significant Networks'.

### Diseases (by Biomarkers) ([TOC](#TOC_table))

Disease folders are organized into a hierarchical tree. Gene content may very greatly between such complex diseases as cancers and some Mendelian diseases. Also, coverage of different diseases in literature is skewed. These two factors may affect p‑value prioritization for diseases.


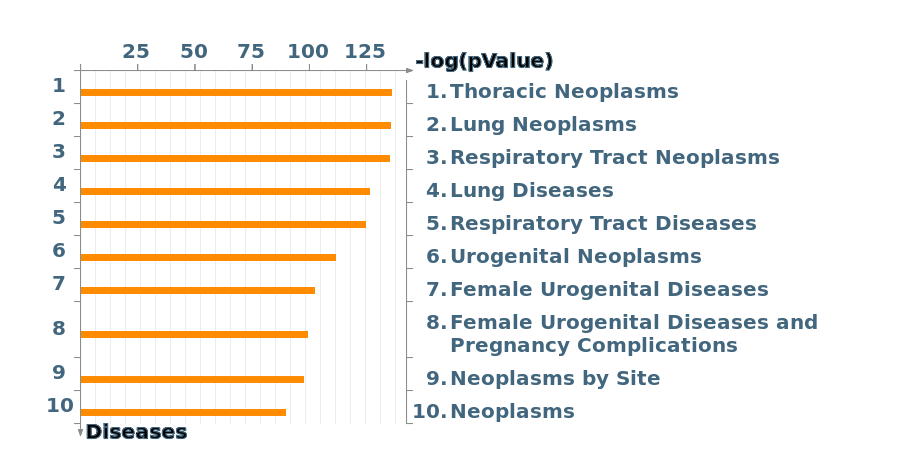


**Figure 9.** Diseases (by Biomarkers). Sorting is done for the 'Statistically significant Diseases'.

### GO Processes ([TOC](#TOC_table))

These are Gene Ontology (GO) cellular processes. As most GO processes have no gene/protein content, the "empty terms" are excluded from p‑value calculations.


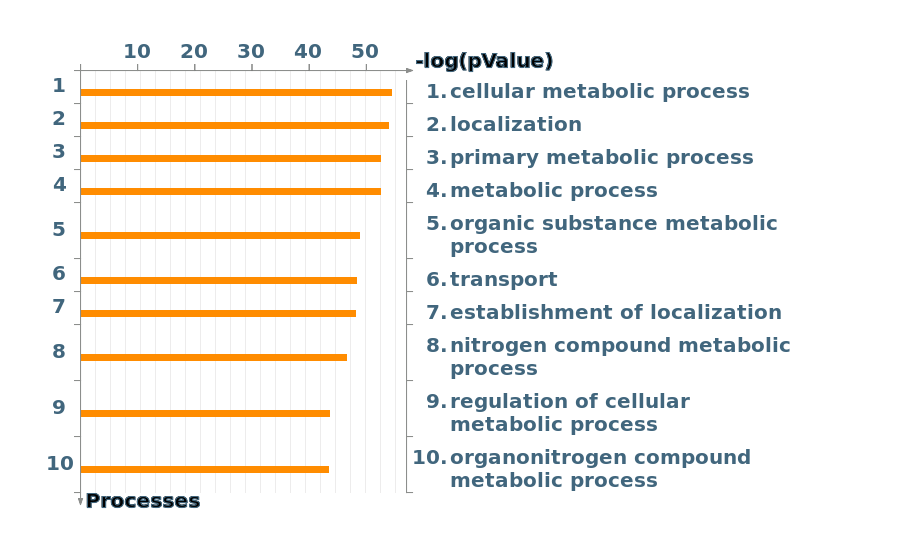


**Figure 10.** GO Processes. Sorting is done for the 'Statistically significant Processes'.

Blue

**Enrichment Analysis Workflow 1.0 Data Analysis Report**

*Server: portal.genego.com*

*Date: 2021‑12‑01*

*Name: NORWEGIAN UNIV OF SCI and TECH | Arnar Flatberg | arnar.flatberg@ntnu.no*

*Login: ntnu3*

Experiments

| 1. |  | Blue |
| --- | --- | --- |

The experiments uploaded for comparative analysis

## Table of content:

**[•](#Bookmark_1)** [Enrichment analysis](#Bookmark_1)

**[•](#Bookmark_2)** [Pathway Maps](#Bookmark_2)

**[•](#Bookmark_3)** [Top maps (sorted by Statistically significant Maps)](#Bookmark_3)

**[•](#Bookmark_4)** [1. Map : Immune response_Antigen presentation by MHC class I, classical pathway](#Bookmark_4)

**[•](#Bookmark_5)** [2. Map : COVID‑19: immune dysregulation](#Bookmark_5)

**[•](#Bookmark_6)** [3. Map : Immune response_Induction of the antigen presentation machinery by IFN‑gamma](#Bookmark_6)

**[•](#Bookmark_7)** [4. Map : Macrophage and dendritic cell phenotype shift in cancer](#Bookmark_7)

**[•](#Bookmark_8)** [5. Map : Oxidative stress_ROS‑induced cellular signaling](#Bookmark_8)

**[•](#Bookmark_9)** [Process Networks](#Bookmark_9)

**[•](#Bookmark_10)** [Diseases (by Biomarkers)](#Bookmark_10)

**[•](#Bookmark_11)** [GO Processes](#Bookmark_11)

## Enrichment analysis ([TOC](#TOC_table))

Enrichment analysis consists of matching gene IDs of possible targets for the "common", "similar" and "unique" sets with gene IDs in functional ontologies in MetaCore. The probability of a random intersection between a set of IDs the size of target list with ontology entities is estimated in p‑value of hypergeometric intersection. The lower p‑value means higher relevance of the entity to the dataset, which shows in higher rating for the entity.

Ontologies available for EA in Enrichment Analysis Workflow:

### Pathway Maps ([TOC](#TOC_table))

Canonical pathway maps represent a set of signaling and metabolic maps covering human in a comprehensive way. All maps are created by Clarivate scientists by a high‑quality manual curation process based on published peer‑reviewed literature. Experimental data is visualized on the maps as blue (for downregulation) and red (upregulation) histograms. The height of the histogram corresponds to the relative expression value for a particular gene/protein.


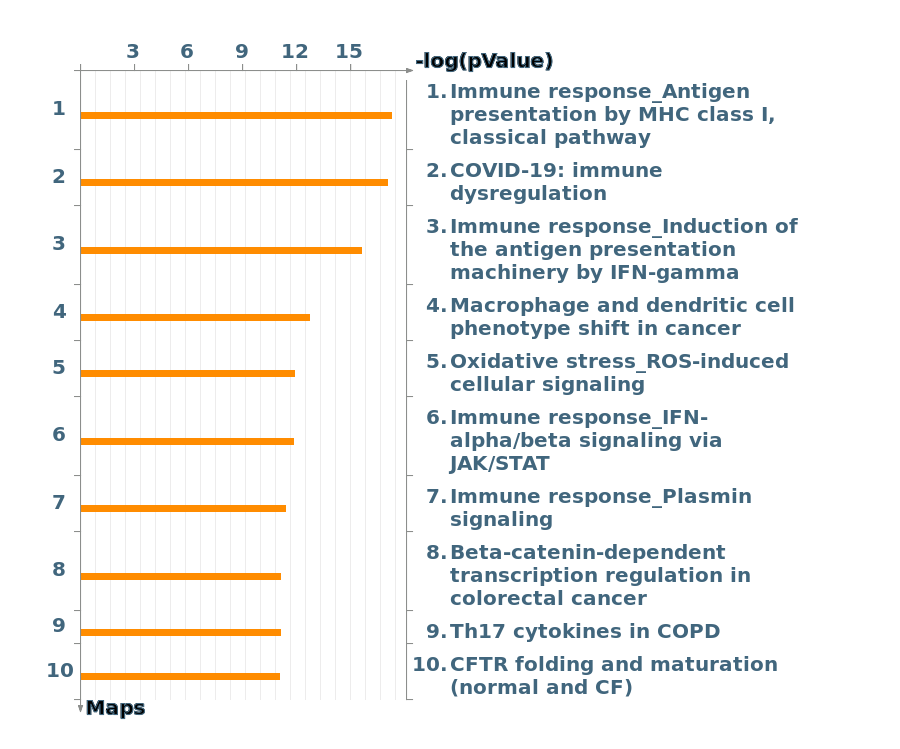


**Figure 2.** Pathway Maps. Sorting is done for the 'Statistically significant Maps'.

### Top maps (sorted by Statistically significant Maps)

**1. Map :** [Immune response_Antigen presentation by MHC class I, classical pathway](https://portal.genego.com/cgi/imagemap.cgi?id=2100)
([TOC](#TOC_table))


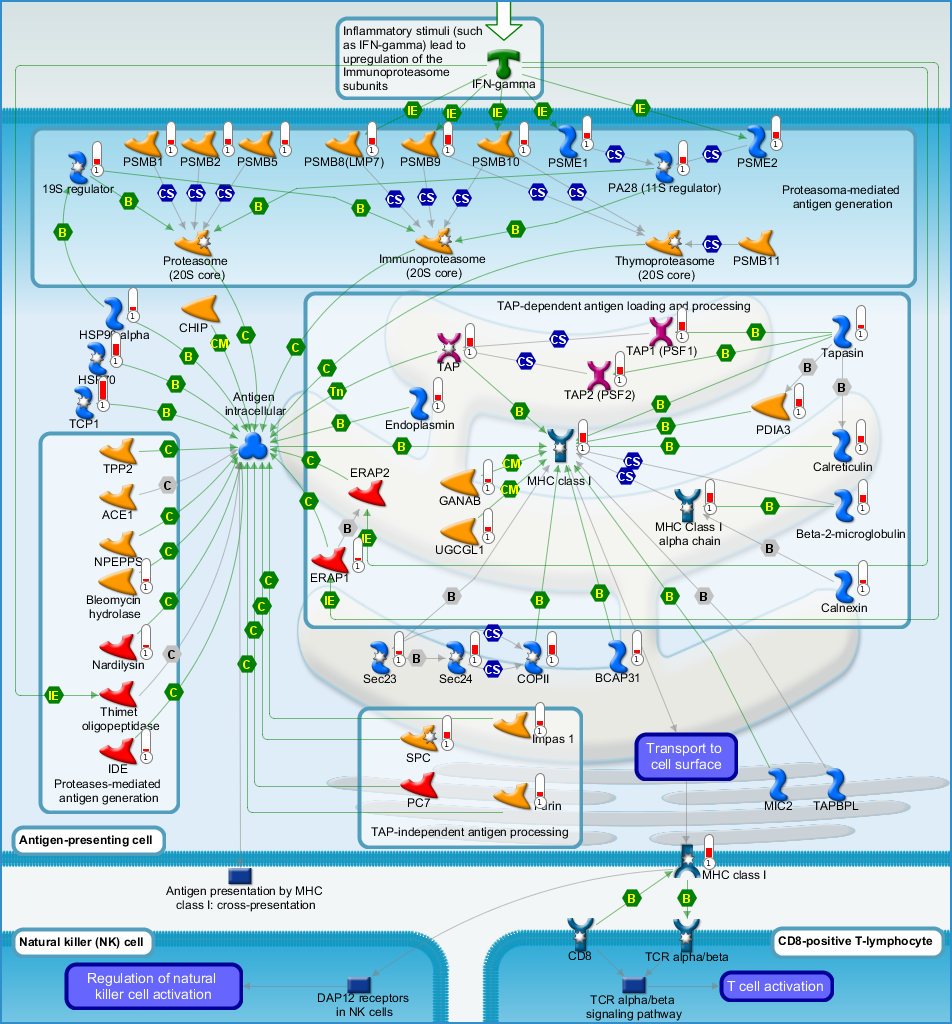


**Figure 3.** The top scored map (map with the the lowest p‑value) based on the enrichment distribution sorted by 'Statistically significant Maps' set. Experimental data from all files is linked to and visualized on the maps as thermometer‑like figures. Up‑ward thermometers have red color and indicate up‑regulated signals and down‑ward (blue) ones indicate down‑regulated expression levels of the genes.

**2. Map :** [COVID‑19: immune dysregulation](https://portal.genego.com/cgi/imagemap.cgi?id=1646)
([TOC](#TOC_table))


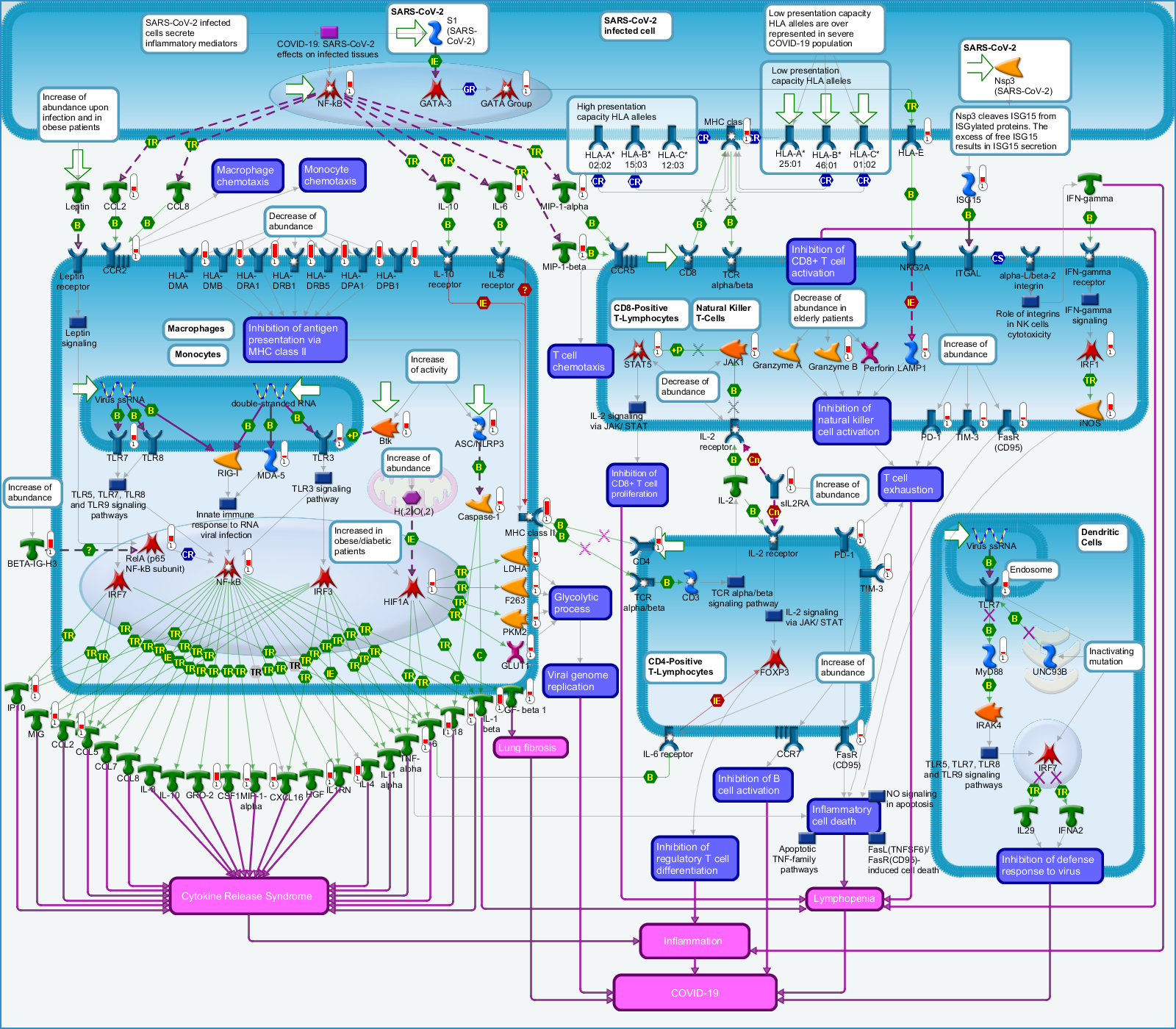


**Figure 4.** The second scored map (map with the second lowest p‑value) based on the enrichment distribution sorted by 'Statistically significant Maps' set. Experimental data from all files is linked to and visualized on the maps as thermometer‑like figures. Up‑ward thermometers have red color and indicate up‑regulated signals and down‑ward (blue) ones indicate down‑regulated expression levels of the genes.

**3. Map :** [Immune response_Induction of the antigen presentation machinery by IFN‑gamma](https://portal.genego.com/cgi/imagemap.cgi?id=2647)
([TOC](#TOC_table))


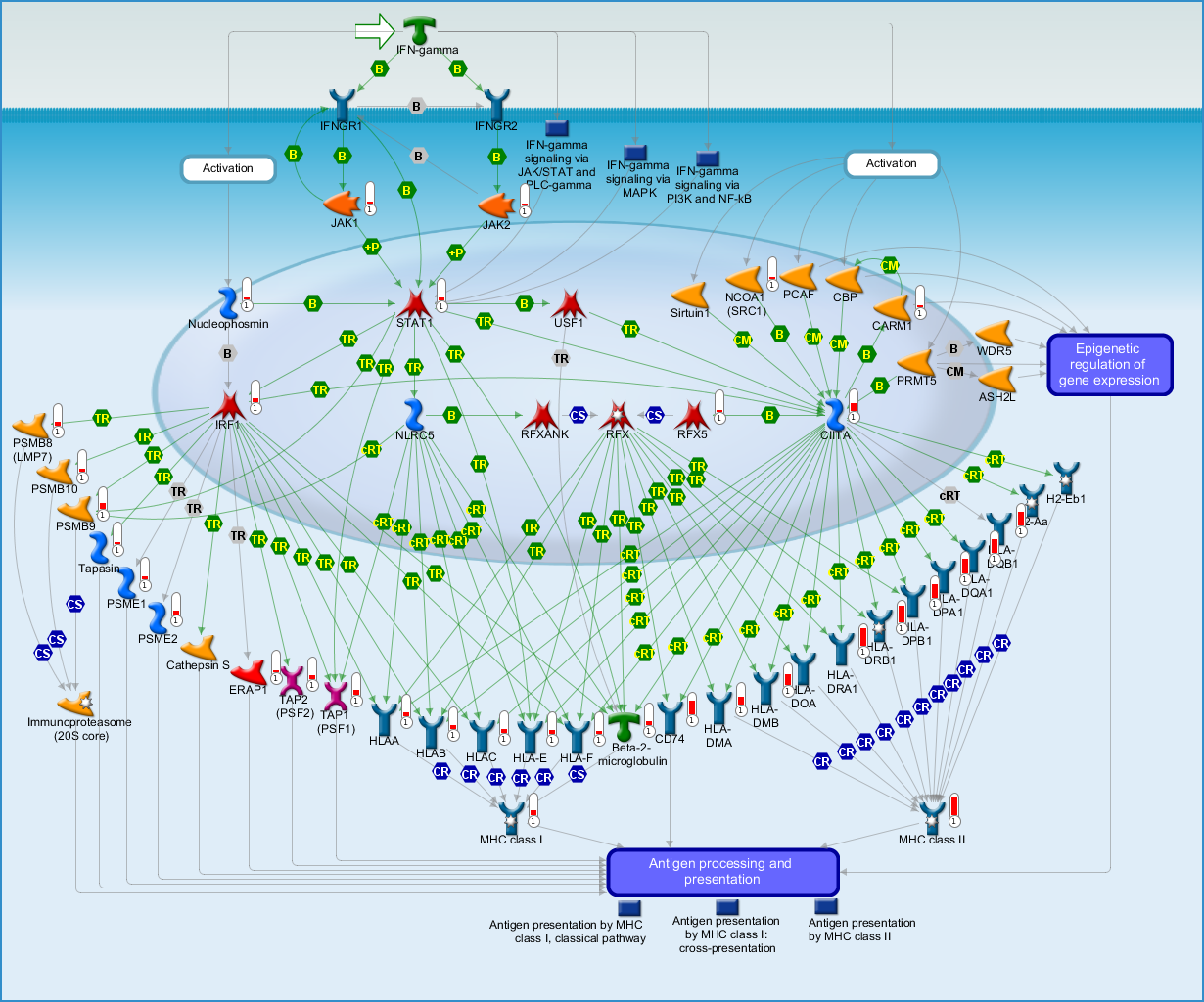


**Figure 5.** The third scored map (map with the third lowest p‑value) based on the enrichment distribution sorted by 'Statistically significant Maps' set. Experimental data from all files is linked to and visualized on the maps as thermometer‑like figures. Up‑ward thermometers have red color and indicate up‑regulated signals and down‑ward (blue) ones indicate down‑regulated expression levels of the genes.

**4. Map :** [Macrophage and dendritic cell phenotype shift in cancer](https://portal.genego.com/cgi/imagemap.cgi?id=7524)
([TOC](#TOC_table))


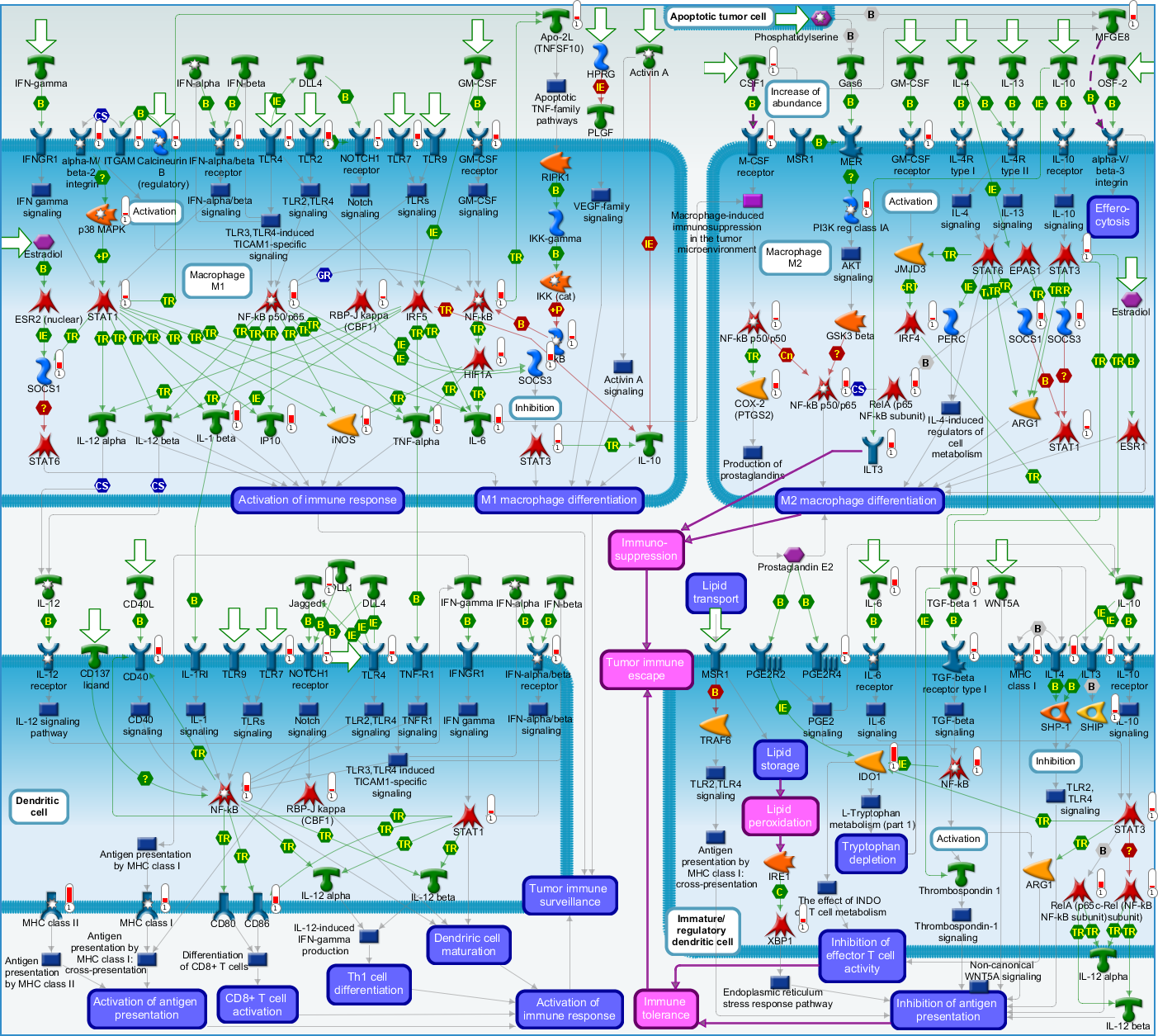


**Figure 6.** The fourth scored map (map with the fourth lowest p‑value) based on the enrichment distribution sorted by 'Statistically significant Maps' set. Experimental data from all files is linked to and visualized on the maps as thermometer‑like figures. Up‑ward thermometers have red color and indicate up‑regulated signals and down‑ward (blue) ones indicate down‑regulated expression levels of the genes.

**5. Map :** [Oxidative stress_ROS‑induced cellular signaling](https://portal.genego.com/cgi/imagemap.cgi?id=7586)
([TOC](#TOC_table))


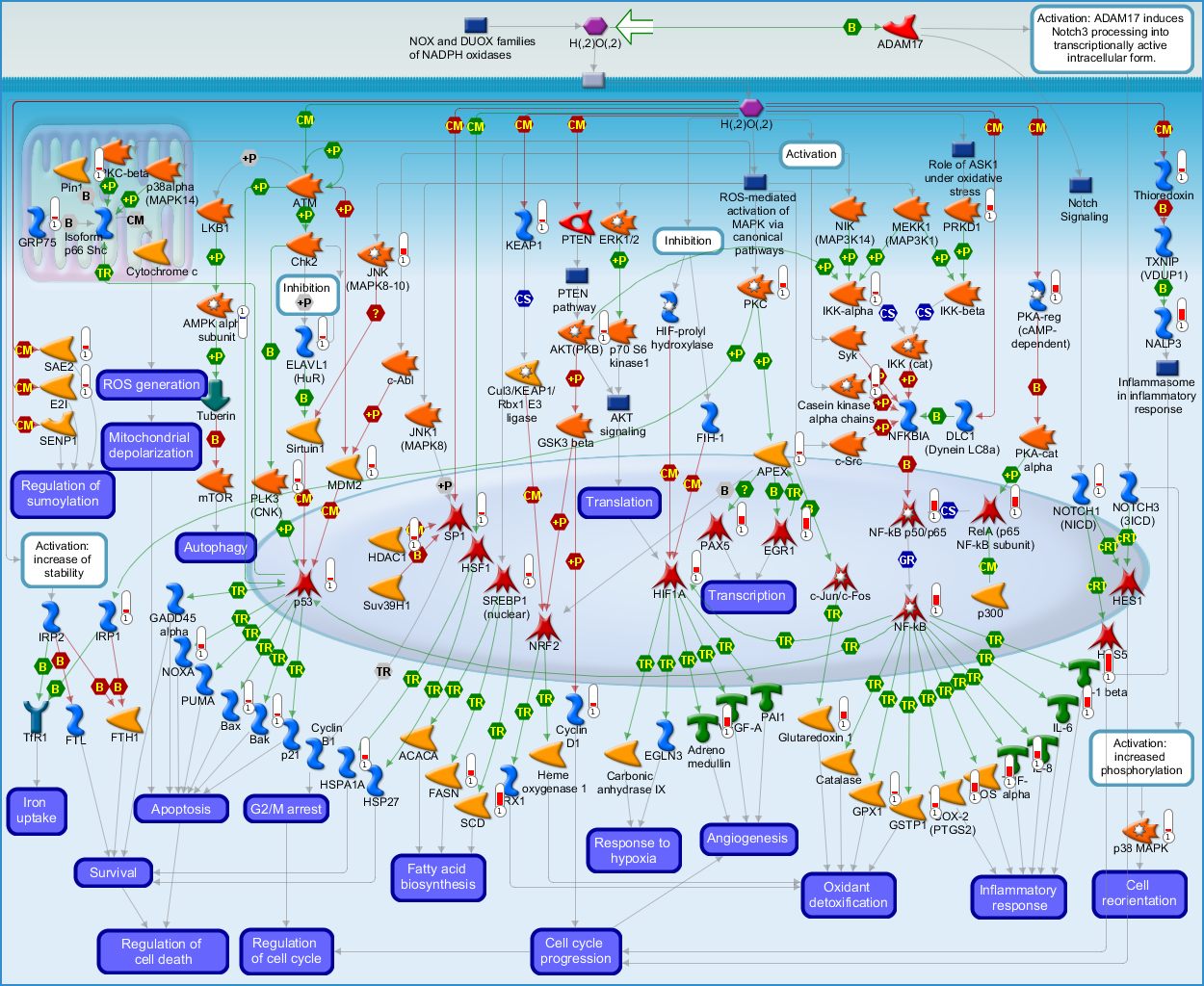


**Figure 7.** The fifth scored map (map with the fifth lowest p‑value) based on the enrichment distribution sorted by 'Statistically significant Maps' set. Experimental data from all files is linked to and visualized on the maps as thermometer‑like figures. Up‑ward thermometers have red color and indicate up‑regulated signals and down‑ward (blue) ones indicate down‑regulated expression levels of the genes.

### Process Networks ([TOC](#TOC_table))

The content of these cellular and molecular processes is defined and annotated by Clarivate scientists. Each process represents a pre‑set network of protein interactions characteristic for the process.


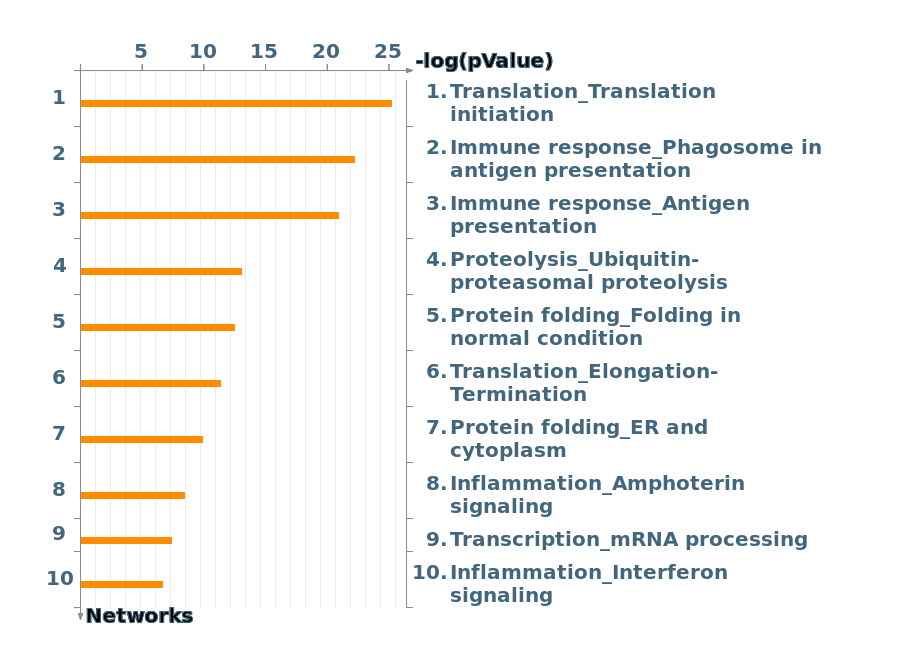


**Figure 8.** Process Networks. Sorting is done for the 'Statistically significant Networks'.

### Diseases (by Biomarkers) ([TOC](#TOC_table))

Disease folders are organized into a hierarchical tree. Gene content may very greatly between such complex diseases as cancers and some Mendelian diseases. Also, coverage of different diseases in literature is skewed. These two factors may affect p‑value prioritization for diseases.


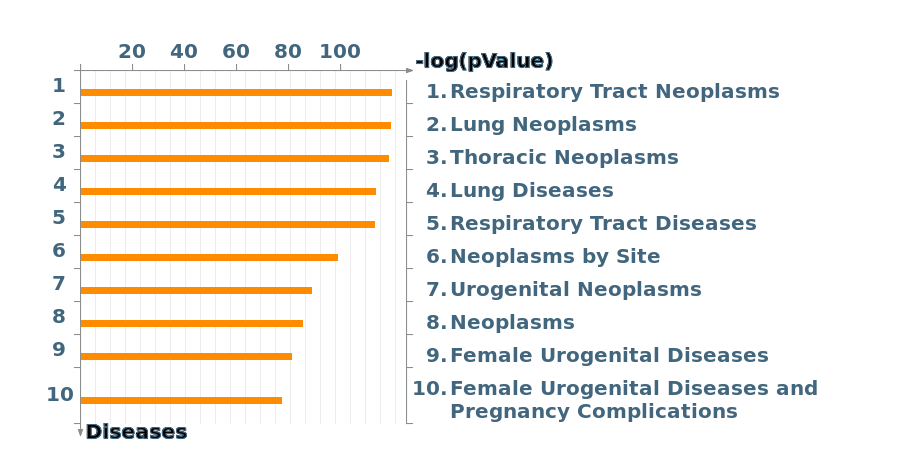


**Figure 9.** Diseases (by Biomarkers). Sorting is done for the 'Statistically significant Diseases'.

### GO Processes ([TOC](#TOC_table))

These are Gene Ontology (GO) cellular processes. As most GO processes have no gene/protein content, the "empty terms" are excluded from p‑value calculations.


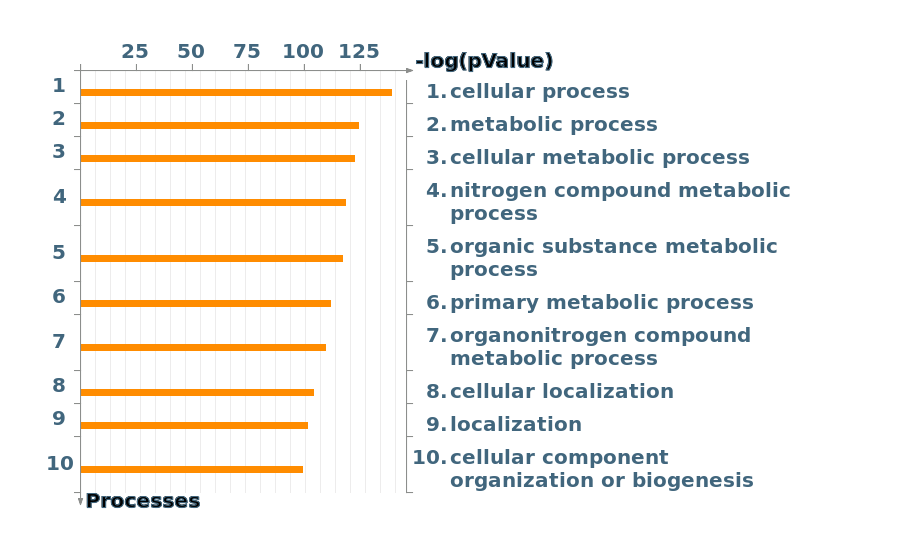


**Figure 10.** GO Processes. Sorting is done for the 'Statistically significant Processes'.

Brown

**Enrichment Analysis Workflow 1.0 Data Analysis Report**

*Server: portal.genego.com*

*Date: 2021‑12‑01*

*Name: NORWEGIAN UNIV OF SCI and TECH | Arnar Flatberg | arnar.flatberg@ntnu.no*

*Login: ntnu3*

Experiments

| 1. |  | Brown |
| --- | --- | --- |

The experiments uploaded for comparative analysis

## Table of content:

**[•](#Bookmark_1)** [Enrichment analysis](#Bookmark_1)

**[•](#Bookmark_2)** [Pathway Maps](#Bookmark_2)

**[•](#Bookmark_3)** [Top maps (sorted by Statistically significant Maps)](#Bookmark_3)

**[•](#Bookmark_4)** [1. Map : Apoptosis and survival_IL‑17‑induced CIKS‑dependent NF‑kB signaling and mRNA stabilization](#Bookmark_4)

**[•](#Bookmark_5)** [2. Map : Chemotaxis_Lysophosphatidic acid signaling via GPCRs](#Bookmark_5)

**[•](#Bookmark_6)** [3. Map : Development_Negative regulation of WNT/Beta‑catenin signaling in the nucleus](#Bookmark_6)

**[•](#Bookmark_7)** [4. Map : Development_Role of HDAC and calcium/calmodulin‑dependent kinase (CaMK) in control of skeletal myogenesis](#Bookmark_7)

**[•](#Bookmark_8)** [5. Map : NF‑AT signaling in cardiac hypertrophy](#Bookmark_8)

**[•](#Bookmark_9)** [Process Networks](#Bookmark_9)

**[•](#Bookmark_10)** [Diseases (by Biomarkers)](#Bookmark_10)

**[•](#Bookmark_11)** [GO Processes](#Bookmark_11)

## Enrichment analysis ([TOC](#TOC_table))

Enrichment analysis consists of matching gene IDs of possible targets for the "common", "similar" and "unique" sets with gene IDs in functional ontologies in MetaCore. The probability of a random intersection between a set of IDs the size of target list with ontology entities is estimated in p‑value of hypergeometric intersection. The lower p‑value means higher relevance of the entity to the dataset, which shows in higher rating for the entity.

Ontologies available for EA in Enrichment Analysis Workflow:

### Pathway Maps ([TOC](#TOC_table))

Canonical pathway maps represent a set of signaling and metabolic maps covering human in a comprehensive way. All maps are created by Clarivate scientists by a high‑quality manual curation process based on published peer‑reviewed literature. Experimental data is visualized on the maps as blue (for downregulation) and red (upregulation) histograms. The height of the histogram corresponds to the relative expression value for a particular gene/protein.


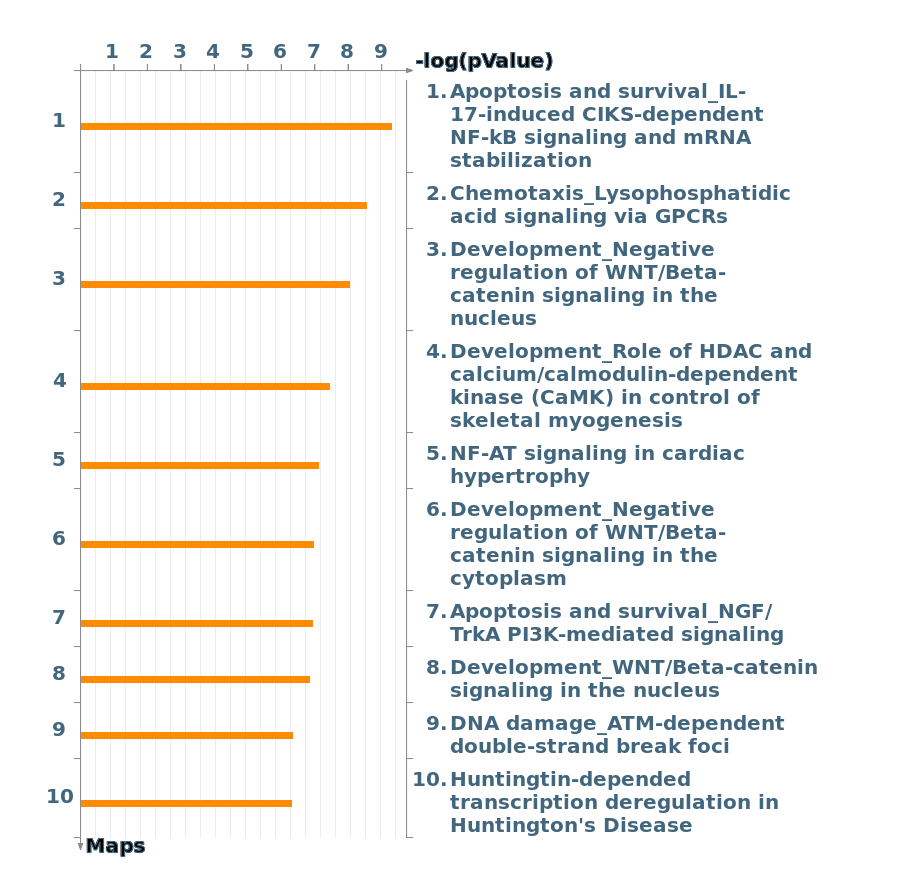


**Figure 2.** Pathway Maps. Sorting is done for the 'Statistically significant Maps'.

### Top maps (sorted by Statistically significant Maps)

**1. Map :** [Apoptosis and survival_IL‑17‑induced CIKS‑dependent NF‑kB signaling and mRNA stabilization](https://portal.genego.com/cgi/imagemap.cgi?id=6601)
([TOC](#TOC_table))


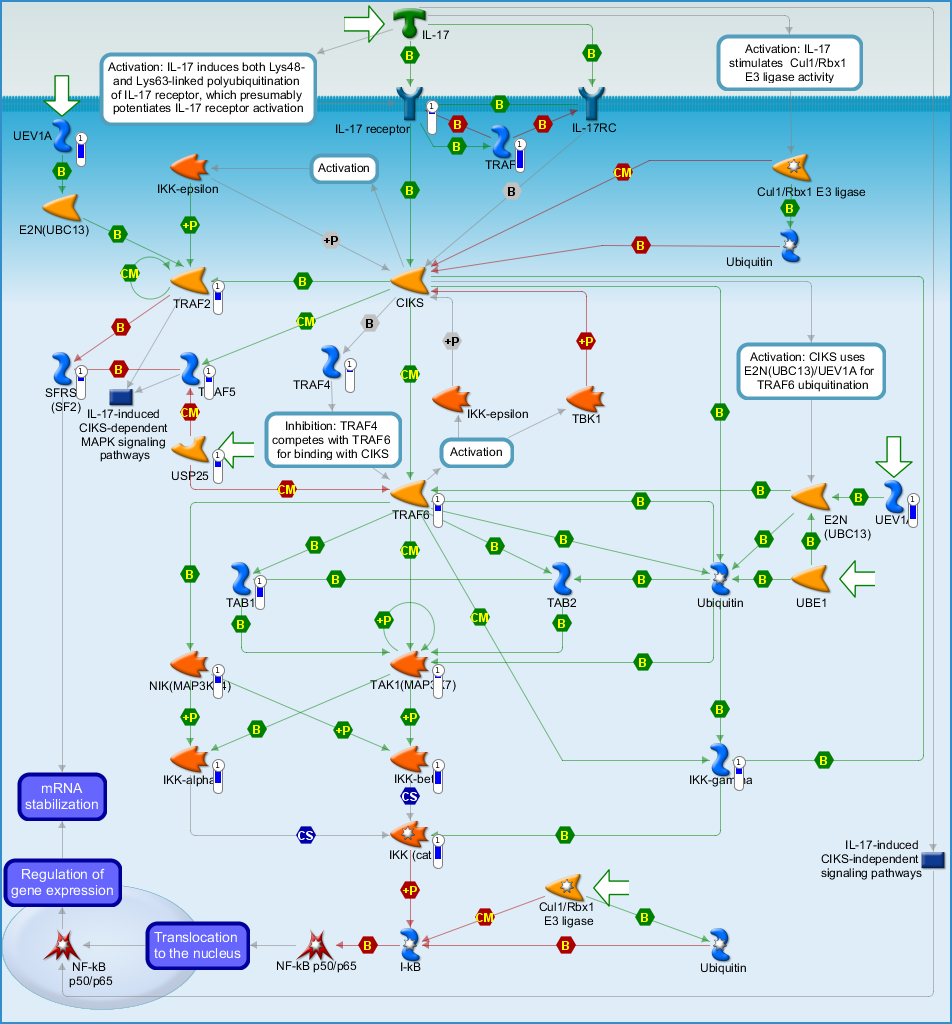


**Figure 3.** The top scored map (map with the the lowest p‑value) based on the enrichment distribution sorted by 'Statistically significant Maps' set. Experimental data from all files is linked to and visualized on the maps as thermometer‑like figures. Up‑ward thermometers have red color and indicate up‑regulated signals and down‑ward (blue) ones indicate down‑regulated expression levels of the genes.

**2. Map :** [Chemotaxis_Lysophosphatidic acid signaling via GPCRs](https://portal.genego.com/cgi/imagemap.cgi?id=452)
([TOC](#TOC_table))


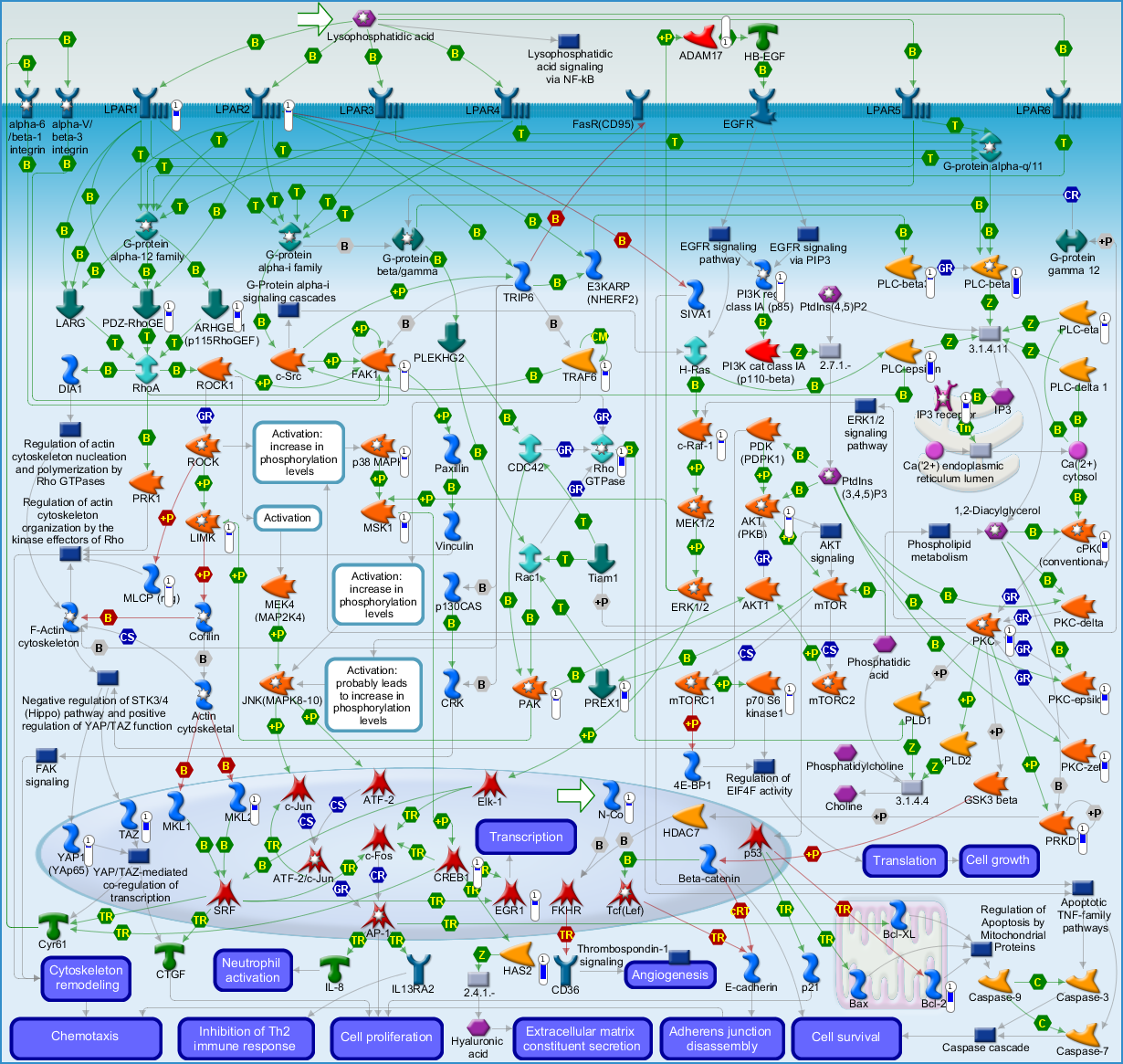


**Figure 4.** The second scored map (map with the second lowest p‑value) based on the enrichment distribution sorted by 'Statistically significant Maps' set. Experimental data from all files is linked to and visualized on the maps as thermometer‑like figures. Up‑ward thermometers have red color and indicate up‑regulated signals and down‑ward (blue) ones indicate down‑regulated expression levels of the genes.

**3. Map :** [Development_Negative regulation of WNT/Beta‑catenin signaling in the nucleus](https://portal.genego.com/cgi/imagemap.cgi?id=2332)
([TOC](#TOC_table))


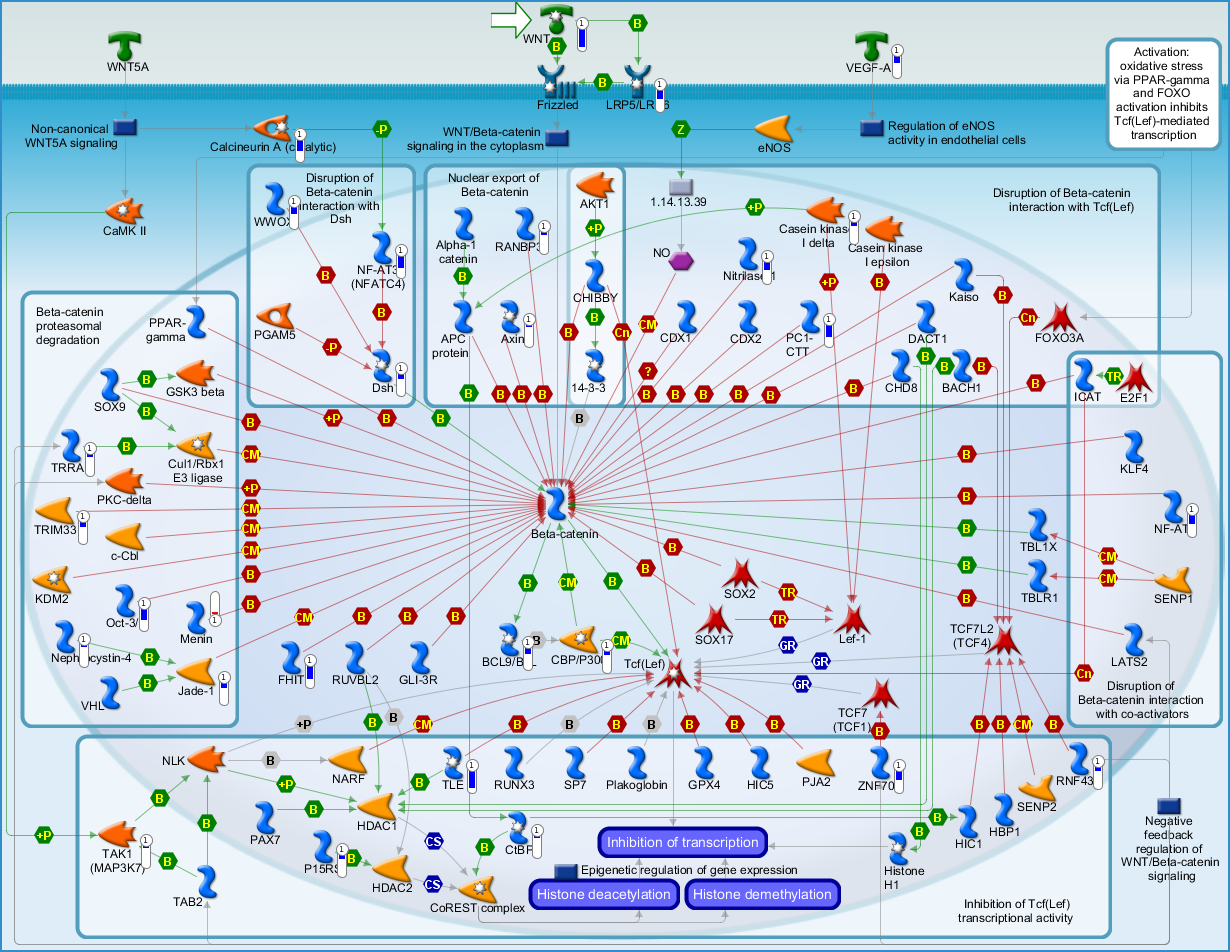


**Figure 5.** The third scored map (map with the third lowest p‑value) based on the enrichment distribution sorted by 'Statistically significant Maps' set. Experimental data from all files is linked to and visualized on the maps as thermometer‑like figures. Up‑ward thermometers have red color and indicate up‑regulated signals and down‑ward (blue) ones indicate down‑regulated expression levels of the genes.

**4. Map :** [Development_Role of HDAC and calcium/calmodulin‑dependent kinase (CaMK) in control of skeletal myogenesis](https://portal.genego.com/cgi/imagemap.cgi?id=440)
([TOC](#TOC_table))


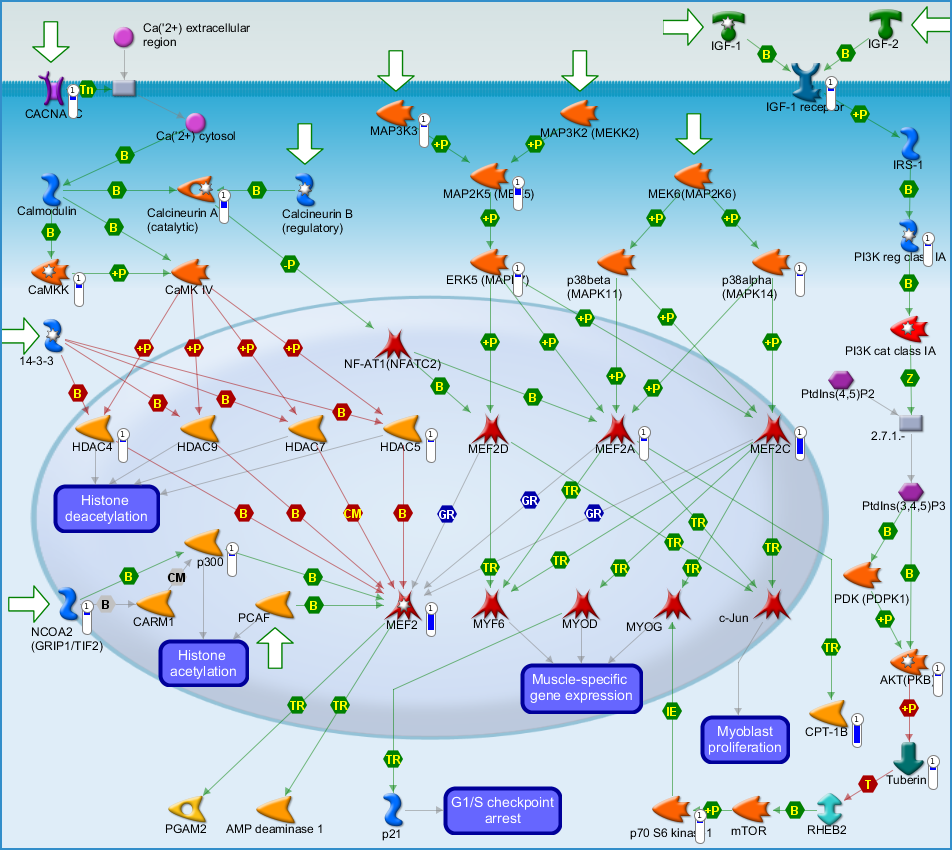


**Figure 6.** The fourth scored map (map with the fourth lowest p‑value) based on the enrichment distribution sorted by 'Statistically significant Maps' set. Experimental data from all files is linked to and visualized on the maps as thermometer‑like figures. Up‑ward thermometers have red color and indicate up‑regulated signals and down‑ward (blue) ones indicate down‑regulated expression levels of the genes.

**5. Map :** [NF‑AT signaling in cardiac hypertrophy](https://portal.genego.com/cgi/imagemap.cgi?id=2235)
([TOC](#TOC_table))


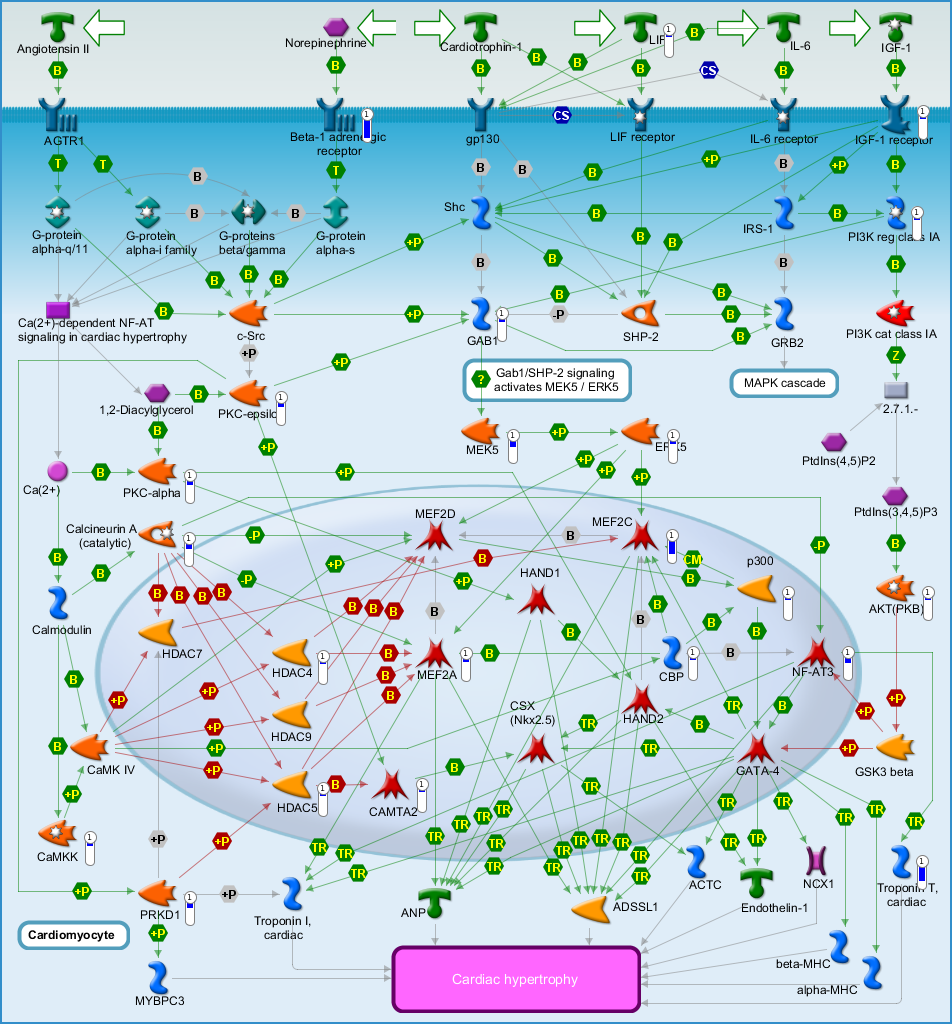


**Figure 7.** The fifth scored map (map with the fifth lowest p‑value) based on the enrichment distribution sorted by 'Statistically significant Maps' set. Experimental data from all files is linked to and visualized on the maps as thermometer‑like figures. Up‑ward thermometers have red color and indicate up‑regulated signals and down‑ward (blue) ones indicate down‑regulated expression levels of the genes.

### Process Networks ([TOC](#TOC_table))

The content of these cellular and molecular processes is defined and annotated by Clarivate scientists. Each process represents a pre‑set network of protein interactions characteristic for the process.


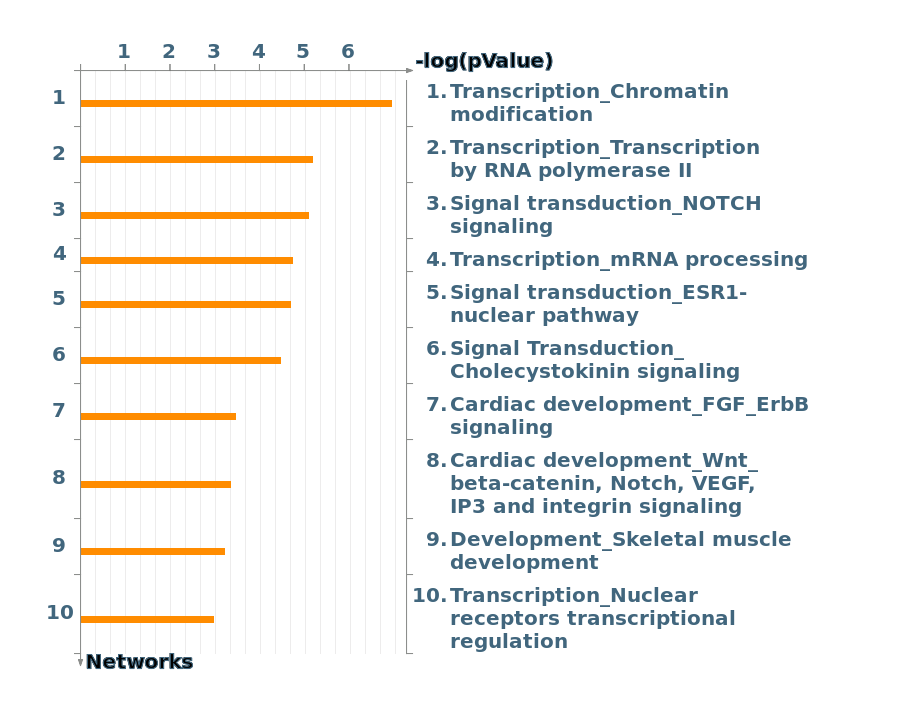


**Figure 8.** Process Networks. Sorting is done for the 'Statistically significant Networks'.

### Diseases (by Biomarkers) ([TOC](#TOC_table))

Disease folders are organized into a hierarchical tree. Gene content may very greatly between such complex diseases as cancers and some Mendelian diseases. Also, coverage of different diseases in literature is skewed. These two factors may affect p‑value prioritization for diseases.


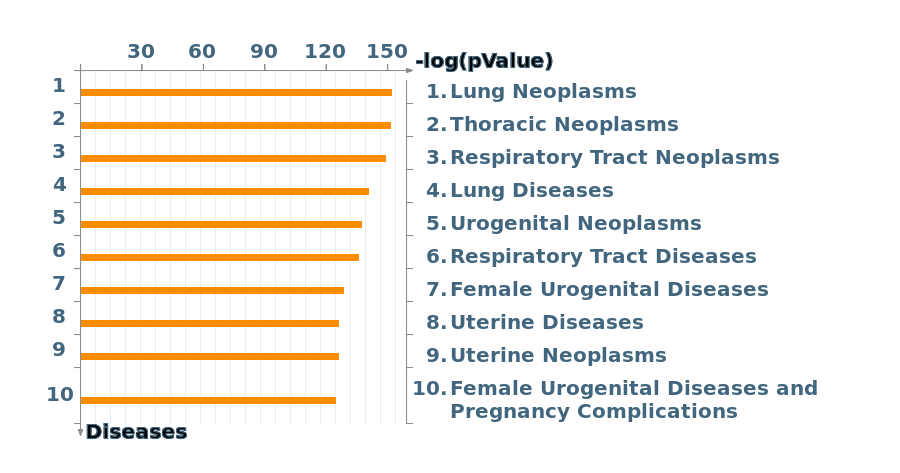


**Figure 9.** Diseases (by Biomarkers). Sorting is done for the 'Statistically significant Diseases'.

### GO Processes ([TOC](#TOC_table))

These are Gene Ontology (GO) cellular processes. As most GO processes have no gene/protein content, the "empty terms" are excluded from p‑value calculations.


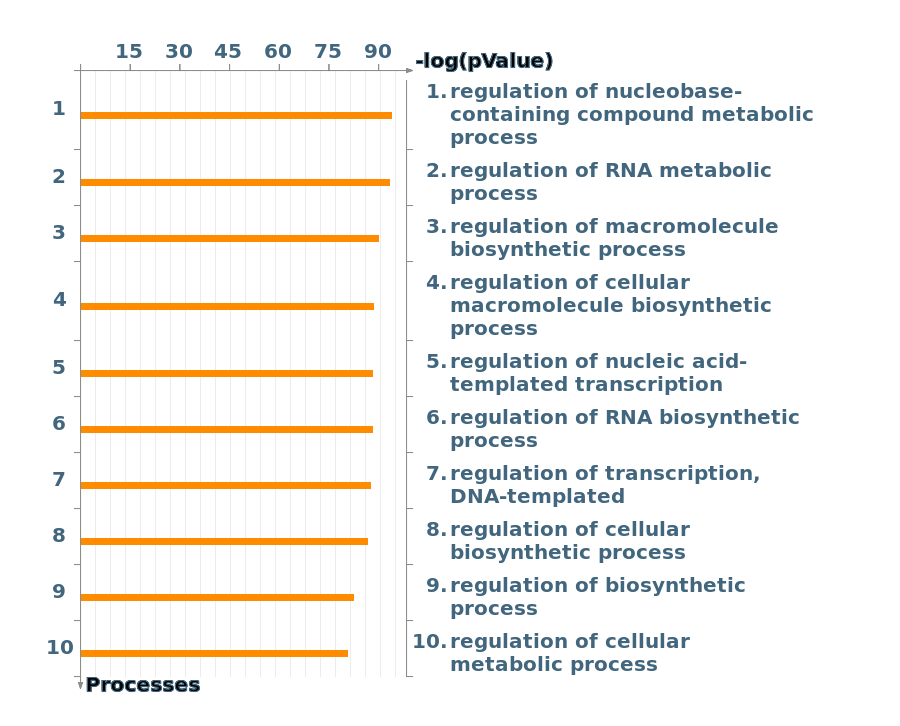


**Figure 10.** GO Processes. Sorting is done for the 'Statistically significant Processes'.

Darkolivegreen

**Enrichment Analysis Workflow 1.0 Data Analysis Report**

*Server: portal.genego.com*

*Date: 2021‑12‑01*

*Name: NORWEGIAN UNIV OF SCI and TECH | Arnar Flatberg | arnar.flatberg@ntnu.no*

*Login: ntnu3*

Experiments

| 1. |  | Darkolivegreen |
| --- | --- | --- |

The experiments uploaded for comparative analysis

## Table of content:

**[•](#Bookmark_1)** [Enrichment analysis](#Bookmark_1)

**[•](#Bookmark_2)** [Pathway Maps](#Bookmark_2)

**[•](#Bookmark_3)** [Top maps (sorted by Statistically significant Maps)](#Bookmark_3)

**[•](#Bookmark_4)** [1. Map : Cell cycle_The metaphase checkpoint](#Bookmark_4)

**[•](#Bookmark_5)** [2. Map : DNA damage_Intra S‑phase checkpoint](#Bookmark_5)

**[•](#Bookmark_6)** [3. Map : Cell cycle_Role of APC in cell cycle regulation](#Bookmark_6)

**[•](#Bookmark_7)** [4. Map : Cell cycle_Start of DNA replication in early S phase](#Bookmark_7)

**[•](#Bookmark_8)** [5. Map : DNA damage_Double‑strand break repair via homologous recombination](#Bookmark_8)

**[•](#Bookmark_9)** [Process Networks](#Bookmark_9)

**[•](#Bookmark_10)** [Diseases (by Biomarkers)](#Bookmark_10)

**[•](#Bookmark_11)** [GO Processes](#Bookmark_11)

## Enrichment analysis ([TOC](#TOC_table))

Enrichment analysis consists of matching gene IDs of possible targets for the "common", "similar" and "unique" sets with gene IDs in functional ontologies in MetaCore. The probability of a random intersection between a set of IDs the size of target list with ontology entities is estimated in p‑value of hypergeometric intersection. The lower p‑value means higher relevance of the entity to the dataset, which shows in higher rating for the entity.

Ontologies available for EA in Enrichment Analysis Workflow:

### Pathway Maps ([TOC](#TOC_table))

Canonical pathway maps represent a set of signaling and metabolic maps covering human in a comprehensive way. All maps are created by Clarivate scientists by a high‑quality manual curation process based on published peer‑reviewed literature. Experimental data is visualized on the maps as blue (for downregulation) and red (upregulation) histograms. The height of the histogram corresponds to the relative expression value for a particular gene/protein.


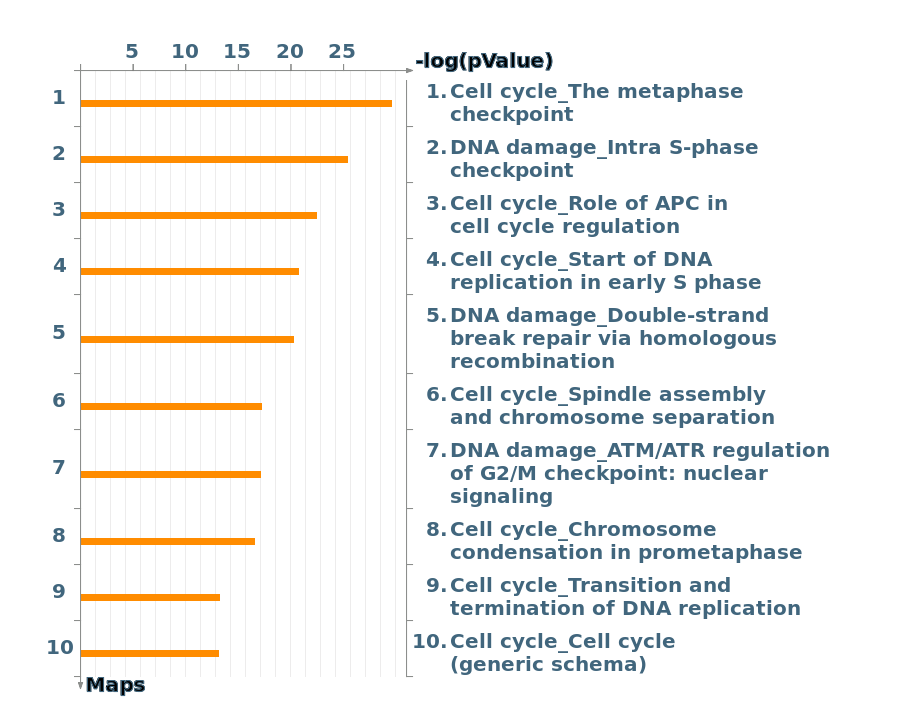


**Figure 2.** Pathway Maps. Sorting is done for the 'Statistically significant Maps'.

### Top maps (sorted by Statistically significant Maps)

**1. Map :** [Cell cycle_The metaphase checkpoint](https://portal.genego.com/cgi/imagemap.cgi?id=711)
([TOC](#TOC_table))


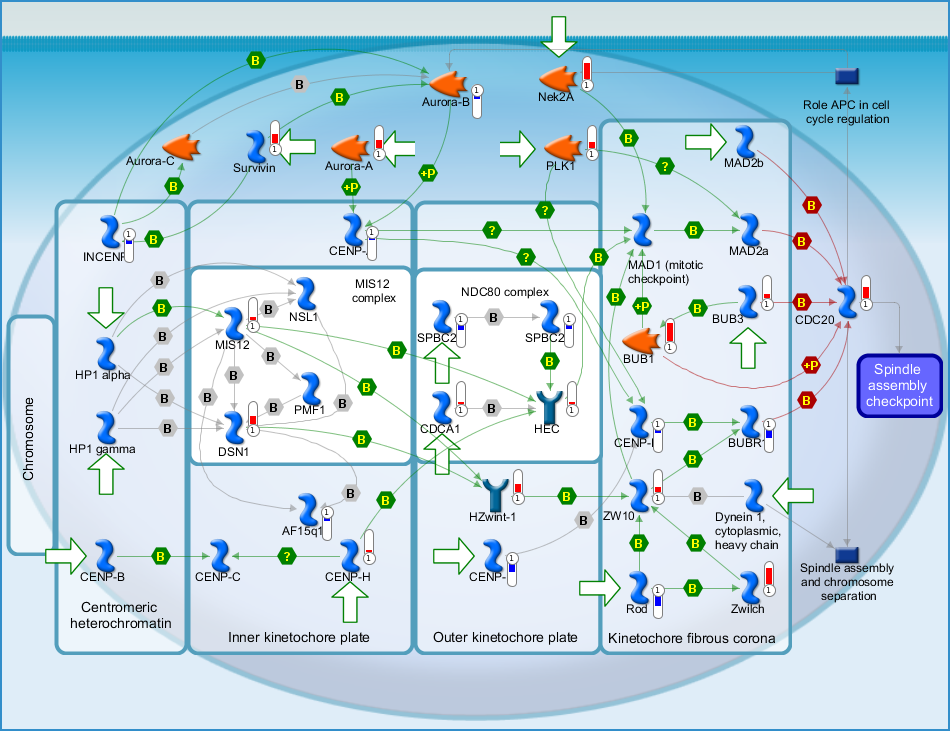


**Figure 3.** The top scored map (map with the the lowest p‑value) based on the enrichment distribution sorted by 'Statistically significant Maps' set. Experimental data from all files is linked to and visualized on the maps as thermometer‑like figures. Up‑ward thermometers have red color and indicate up‑regulated signals and down‑ward (blue) ones indicate down‑regulated expression levels of the genes.

**2. Map :** [DNA damage_Intra S‑phase checkpoint](https://portal.genego.com/cgi/imagemap.cgi?id=2983)
([TOC](#TOC_table))


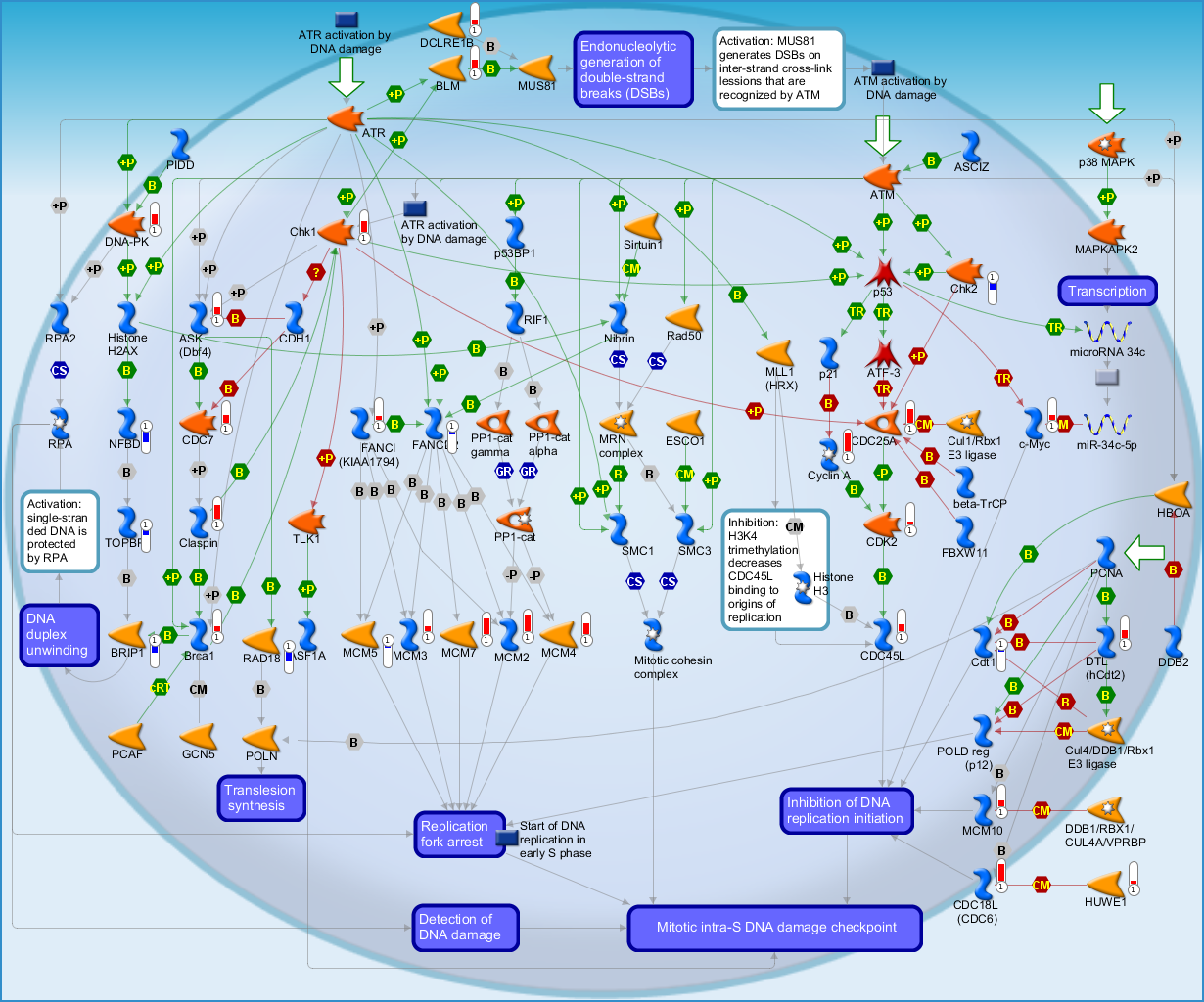


**Figure 4.** The second scored map (map with the second lowest p‑value) based on the enrichment distribution sorted by 'Statistically significant Maps' set. Experimental data from all files is linked to and visualized on the maps as thermometer‑like figures. Up‑ward thermometers have red color and indicate up‑regulated signals and down‑ward (blue) ones indicate down‑regulated expression levels of the genes.

**3. Map :** [Cell cycle_Role of APC in cell cycle regulation](https://portal.genego.com/cgi/imagemap.cgi?id=472)
([TOC](#TOC_table))


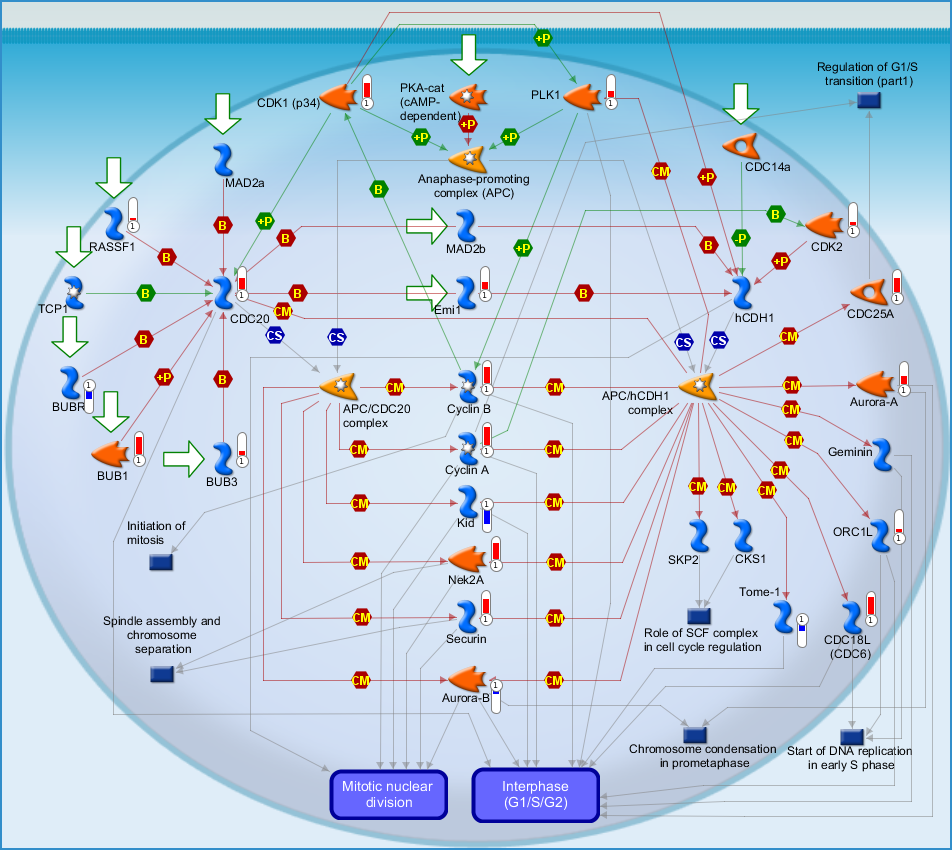


**Figure 5.** The third scored map (map with the third lowest p‑value) based on the enrichment distribution sorted by 'Statistically significant Maps' set. Experimental data from all files is linked to and visualized on the maps as thermometer‑like figures. Up‑ward thermometers have red color and indicate up‑regulated signals and down‑ward (blue) ones indicate down‑regulated expression levels of the genes.

**4. Map :** [Cell cycle_Start of DNA replication in early S phase](https://portal.genego.com/cgi/imagemap.cgi?id=705)
([TOC](#TOC_table))


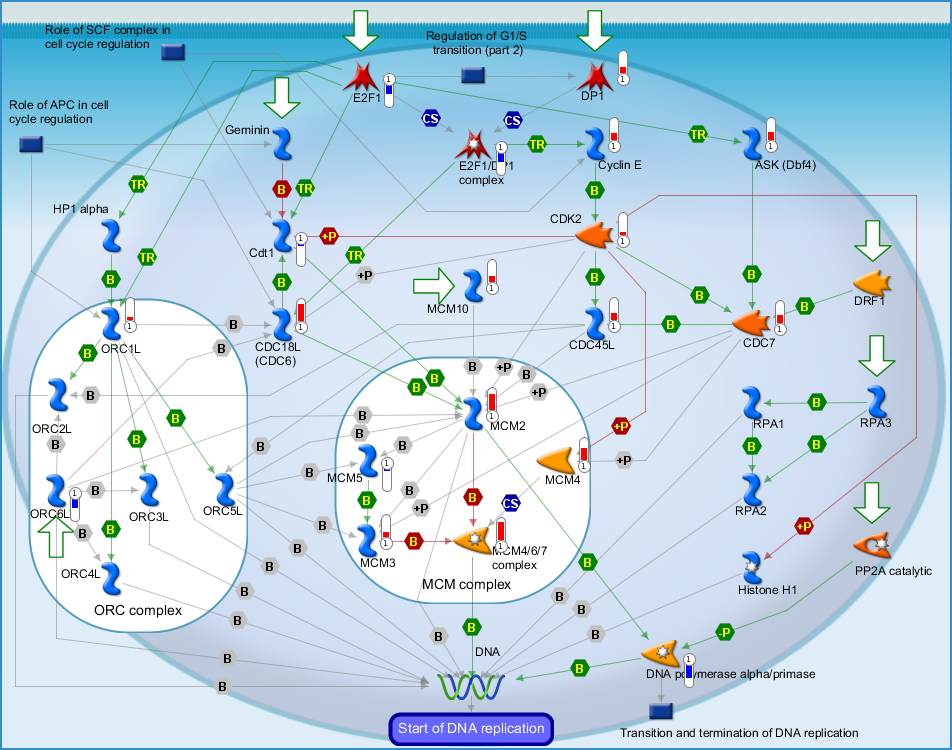


**Figure 6.** The fourth scored map (map with the fourth lowest p‑value) based on the enrichment distribution sorted by 'Statistically significant Maps' set. Experimental data from all files is linked to and visualized on the maps as thermometer‑like figures. Up‑ward thermometers have red color and indicate up‑regulated signals and down‑ward (blue) ones indicate down‑regulated expression levels of the genes.

**5. Map :** [DNA damage_Double‑strand break repair via homologous recombination](https://portal.genego.com/cgi/imagemap.cgi?id=427)
([TOC](#TOC_table))


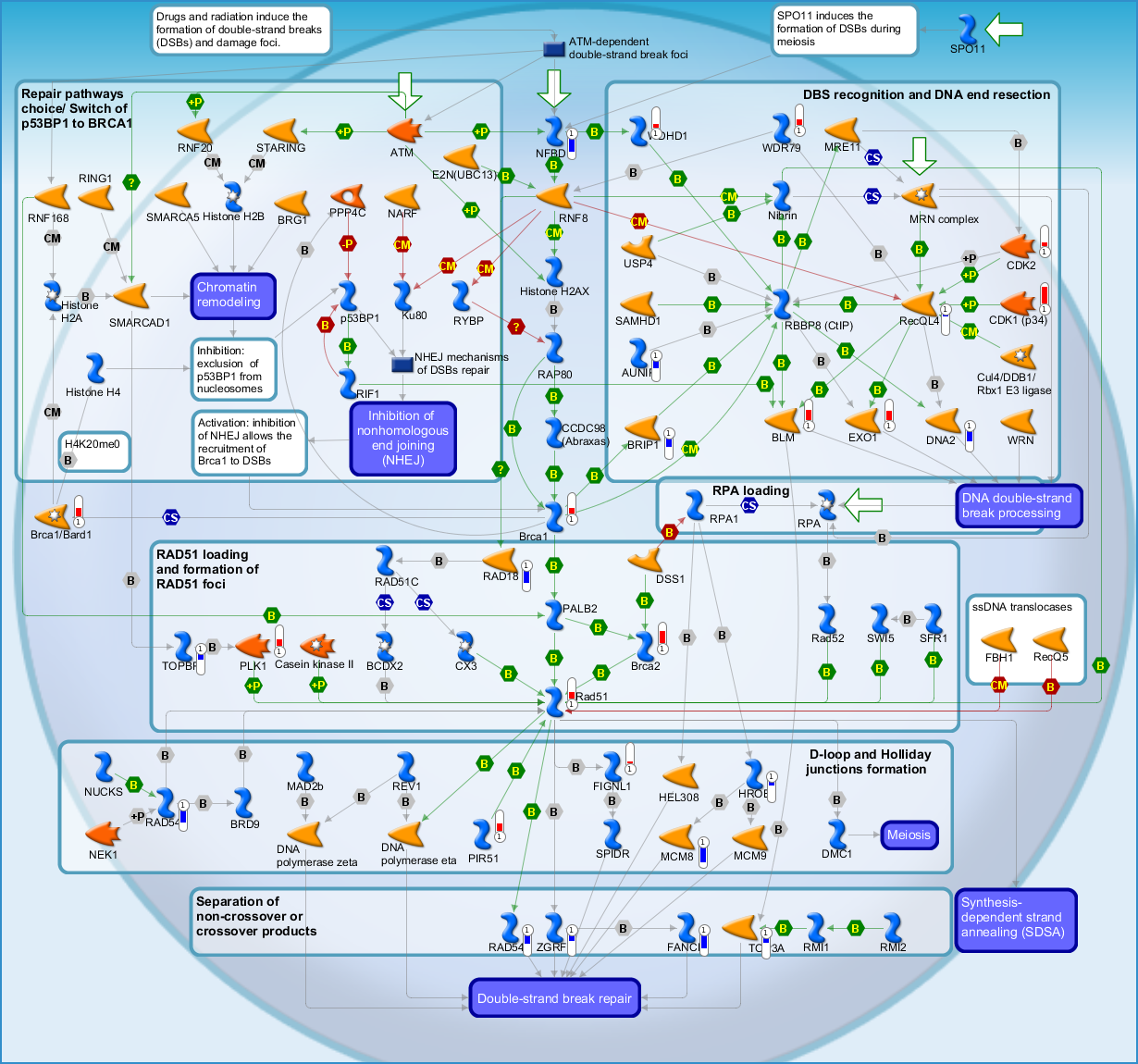


**Figure 7.** The fifth scored map (map with the fifth lowest p‑value) based on the enrichment distribution sorted by 'Statistically significant Maps' set. Experimental data from all files is linked to and visualized on the maps as thermometer‑like figures. Up‑ward thermometers have red color and indicate up‑regulated signals and down‑ward (blue) ones indicate down‑regulated expression levels of the genes.

### Process Networks ([TOC](#TOC_table))

The content of these cellular and molecular processes is defined and annotated by Clarivate scientists. Each process represents a pre‑set network of protein interactions characteristic for the process.


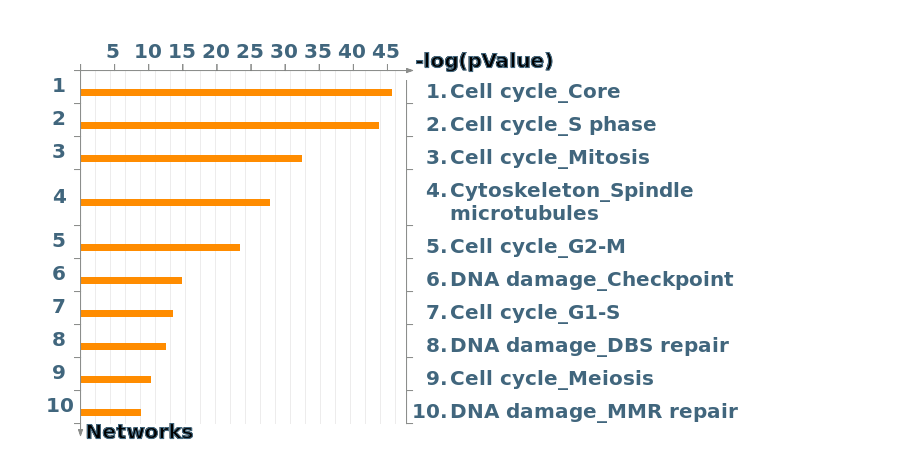


**Figure 8.** Process Networks. Sorting is done for the 'Statistically significant Networks'.

### Diseases (by Biomarkers) ([TOC](#TOC_table))

Disease folders are organized into a hierarchical tree. Gene content may very greatly between such complex diseases as cancers and some Mendelian diseases. Also, coverage of different diseases in literature is skewed. These two factors may affect p‑value prioritization for diseases.


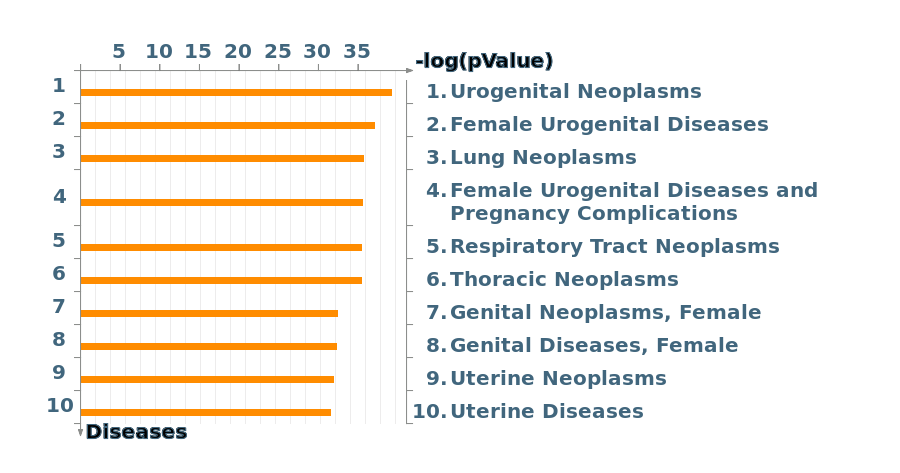


**Figure 9.** Diseases (by Biomarkers). Sorting is done for the 'Statistically significant Diseases'.

### GO Processes ([TOC](#TOC_table))

These are Gene Ontology (GO) cellular processes. As most GO processes have no gene/protein content, the "empty terms" are excluded from p‑value calculations.


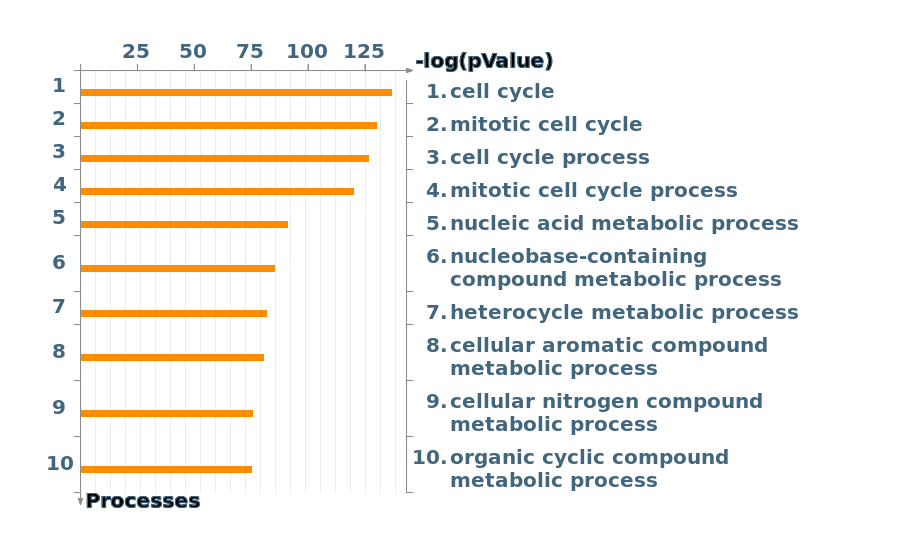


**Figure 10.** GO Processes. Sorting is done for the 'Statistically significant Processes'.

Floralwhit**e**

**Enrichment Analysis Workflow 1.0 Data Analysis Report**

*Server: portal.genego.com*

*Date: 2021‑12‑01*

*Name: NORWEGIAN UNIV OF SCI and TECH | Arnar Flatberg | arnar.flatberg@ntnu.no*

*Login: ntnu3*

Experiments

| 1. |  | Floralwhite |
| --- | --- | --- |

The experiments uploaded for comparative analysis

## Table of content:

**[•](#Bookmark_1)** [Enrichment analysis](#Bookmark_1)

**[•](#Bookmark_2)** [Pathway Maps](#Bookmark_2)

**[•](#Bookmark_3)** [Top maps (sorted by Statistically significant Maps)](#Bookmark_3)

**[•](#Bookmark_4)** [1. Map : Immune response_IL‑5 signaling via JAK/STAT](#Bookmark_4)

**[•](#Bookmark_5)** [2. Map : Immune response_Innate immune response to RNA viral infection](#Bookmark_5)

**[•](#Bookmark_6)** [3. Map : Signal transduction_Non‑apoptotic FasR(CD95) signaling](#Bookmark_6)

**[•](#Bookmark_7)** [4. Map : Role of Apo‑2L(TNFSF10) in Prostate Cancer cell apoptosis](#Bookmark_7)

**[•](#Bookmark_8)** [5. Map : Immune response_IL‑3 signaling via JAK/STAT, p38, JNK and NF‑kB](#Bookmark_8)

**[•](#Bookmark_9)** [Process Networks](#Bookmark_9)

**[•](#Bookmark_10)** [Diseases (by Biomarkers)](#Bookmark_10)

**[•](#Bookmark_11)** [GO Processes](#Bookmark_11)

## Enrichment analysis ([TOC](#TOC_table))

Enrichment analysis consists of matching gene IDs of possible targets for the "common", "similar" and "unique" sets with gene IDs in functional ontologies in MetaCore. The probability of a random intersection between a set of IDs the size of target list with ontology entities is estimated in p‑value of hypergeometric intersection. The lower p‑value means higher relevance of the entity to the dataset, which shows in higher rating for the entity.

Ontologies available for EA in Enrichment Analysis Workflow:

### Pathway Maps ([TOC](#TOC_table))

Canonical pathway maps represent a set of signaling and metabolic maps covering human in a comprehensive way. All maps are created by Clarivate scientists by a high‑quality manual curation process based on published peer‑reviewed literature. Experimental data is visualized on the maps as blue (for downregulation) and red (upregulation) histograms. The height of the histogram corresponds to the relative expression value for a particular gene/protein.


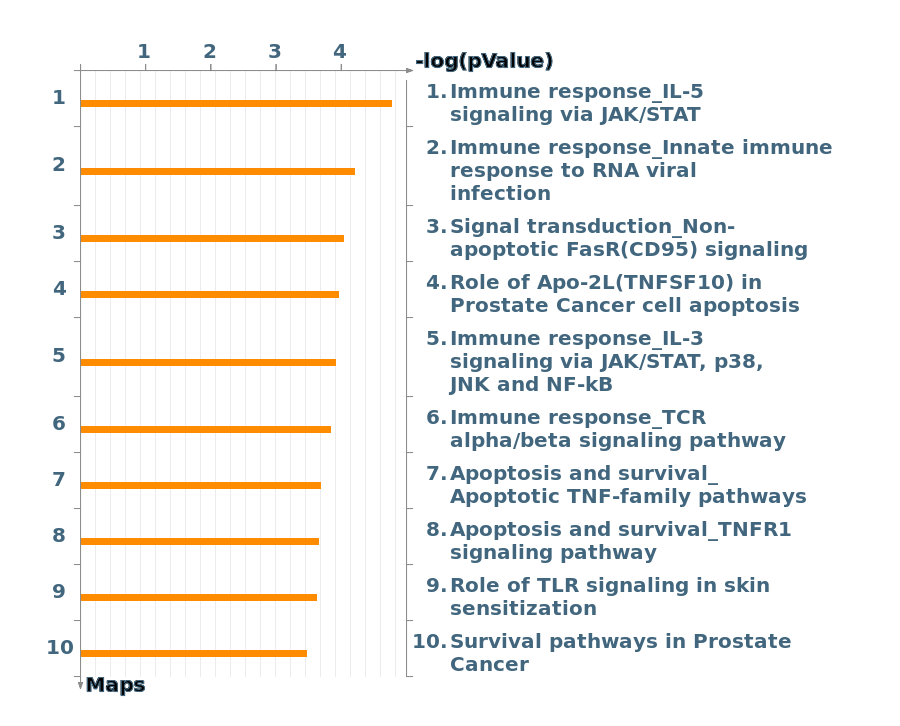


**Figure 2.** Pathway Maps. Sorting is done for the 'Statistically significant Maps'.

### Top maps (sorted by Statistically significant Maps)

**1. Map :** [Immune response_IL‑5 signaling via JAK/STAT](https://portal.genego.com/cgi/imagemap.cgi?id=3262)
([TOC](#TOC_table))


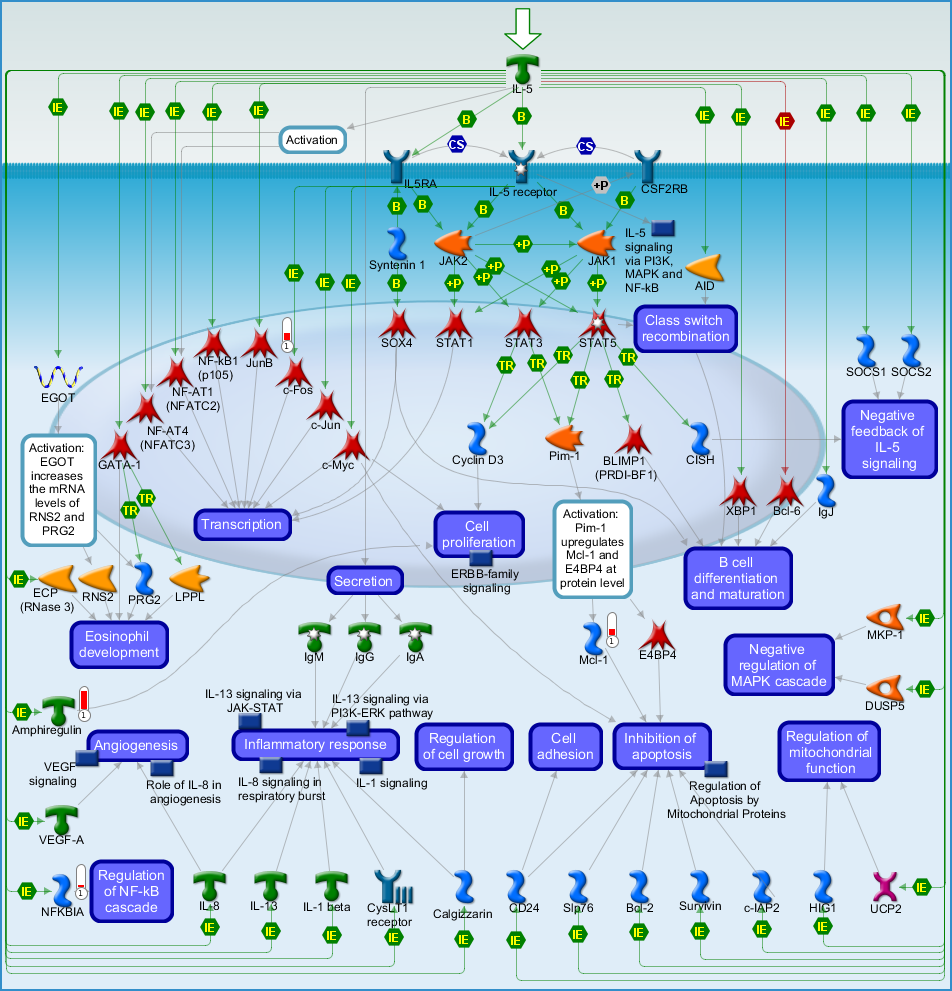


**Figure 3.** The top scored map (map with the the lowest p‑value) based on the enrichment distribution sorted by 'Statistically significant Maps' set. Experimental data from all files is linked to and visualized on the maps as thermometer‑like figures. Up‑ward thermometers have red color and indicate up‑regulated signals and down‑ward (blue) ones indicate down‑regulated expression levels of the genes.

**2. Map :** [Immune response_Innate immune response to RNA viral infection](https://portal.genego.com/cgi/imagemap.cgi?id=3076)
([TOC](#TOC_table))


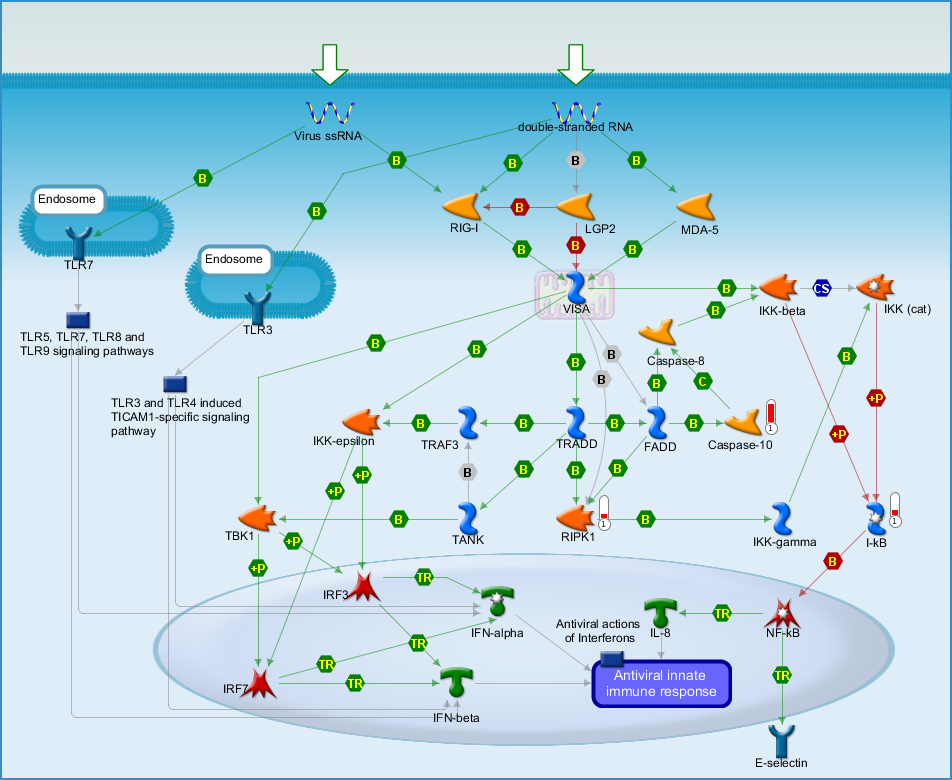


**Figure 4.** The second scored map (map with the second lowest p‑value) based on the enrichment distribution sorted by 'Statistically significant Maps' set. Experimental data from all files is linked to and visualized on the maps as thermometer‑like figures. Up‑ward thermometers have red color and indicate up‑regulated signals and down‑ward (blue) ones indicate down‑regulated expression levels of the genes.

**3. Map :** [Signal transduction_Non‑apoptotic FasR(CD95) signaling](https://portal.genego.com/cgi/imagemap.cgi?id=418)
([TOC](#TOC_table))


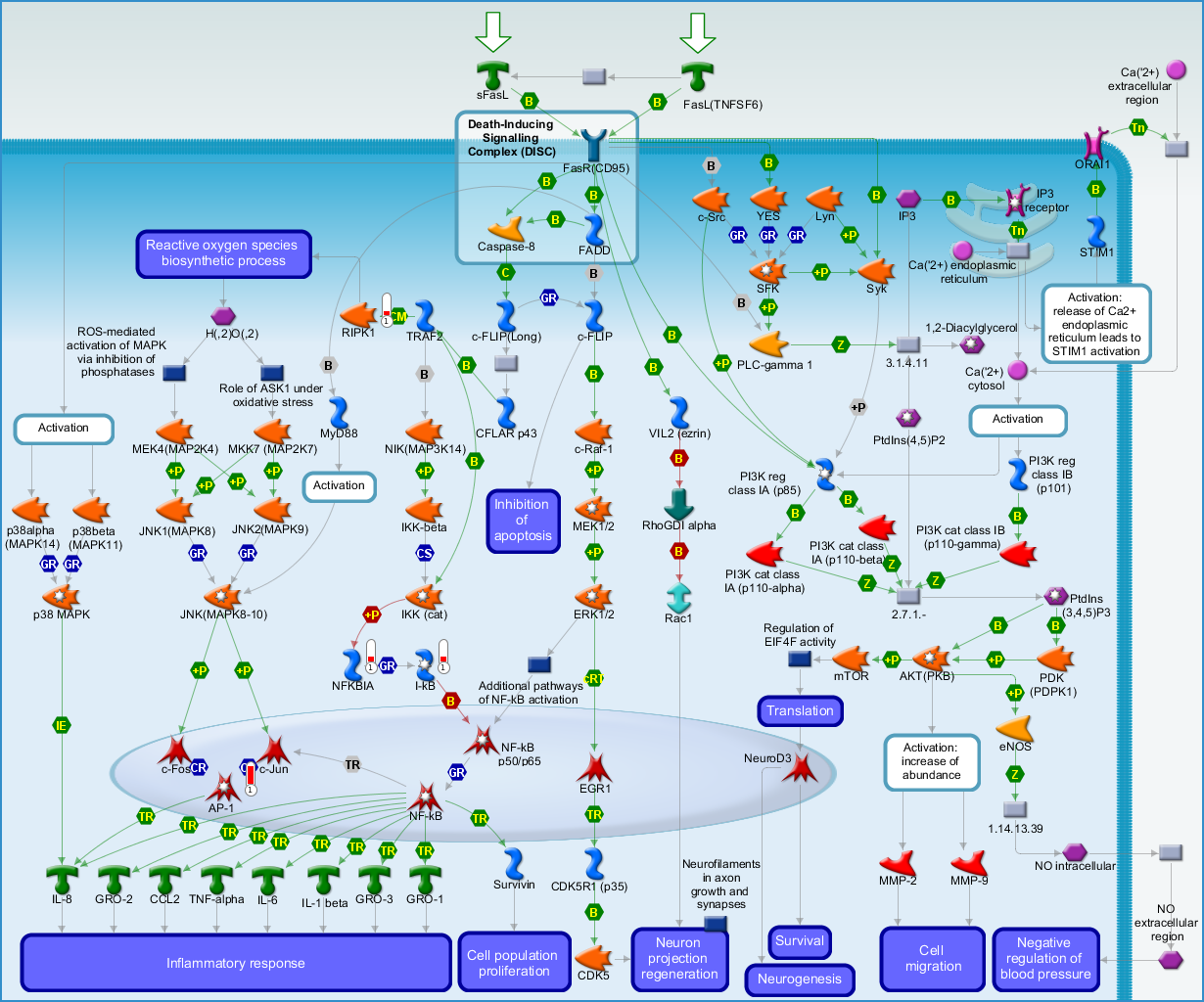


**Figure 5.** The third scored map (map with the third lowest p‑value) based on the enrichment distribution sorted by 'Statistically significant Maps' set. Experimental data from all files is linked to and visualized on the maps as thermometer‑like figures. Up‑ward thermometers have red color and indicate up‑regulated signals and down‑ward (blue) ones indicate down‑regulated expression levels of the genes.

**4. Map :** [Role of Apo‑2L(TNFSF10) in Prostate Cancer cell apoptosis](https://portal.genego.com/cgi/imagemap.cgi?id=3107)
([TOC](#TOC_table))


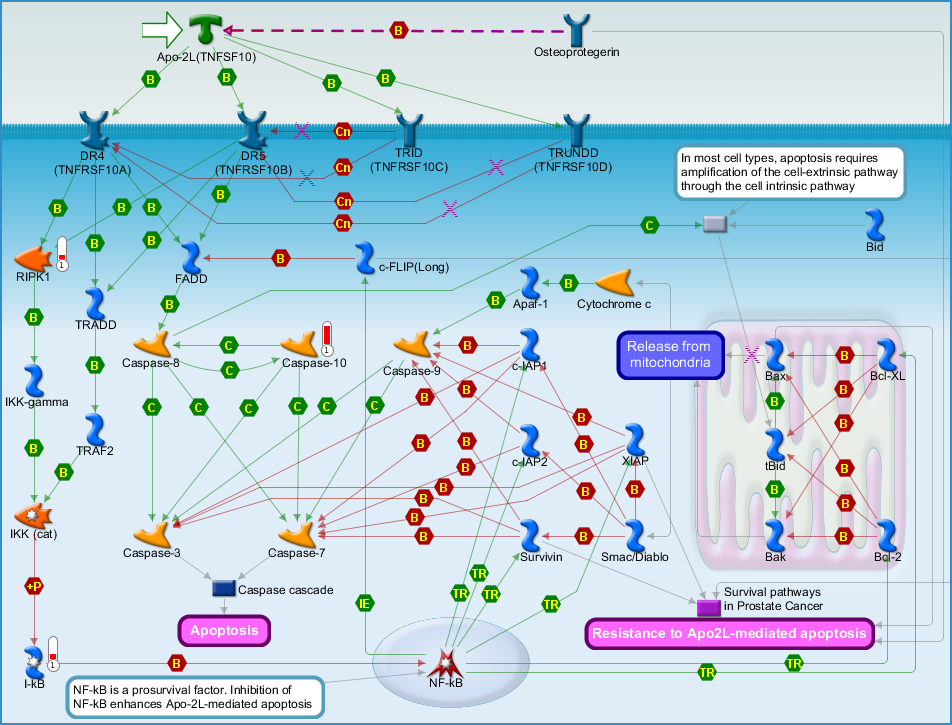


**Figure 6.** The fourth scored map (map with the fourth lowest p‑value) based on the enrichment distribution sorted by 'Statistically significant Maps' set. Experimental data from all files is linked to and visualized on the maps as thermometer‑like figures. Up‑ward thermometers have red color and indicate up‑regulated signals and down‑ward (blue) ones indicate down‑regulated expression levels of the genes.

**5. Map :** [Immune response_IL‑3 signaling via JAK/STAT, p38, JNK and NF‑kB](https://portal.genego.com/cgi/imagemap.cgi?id=657)
([TOC](#TOC_table))


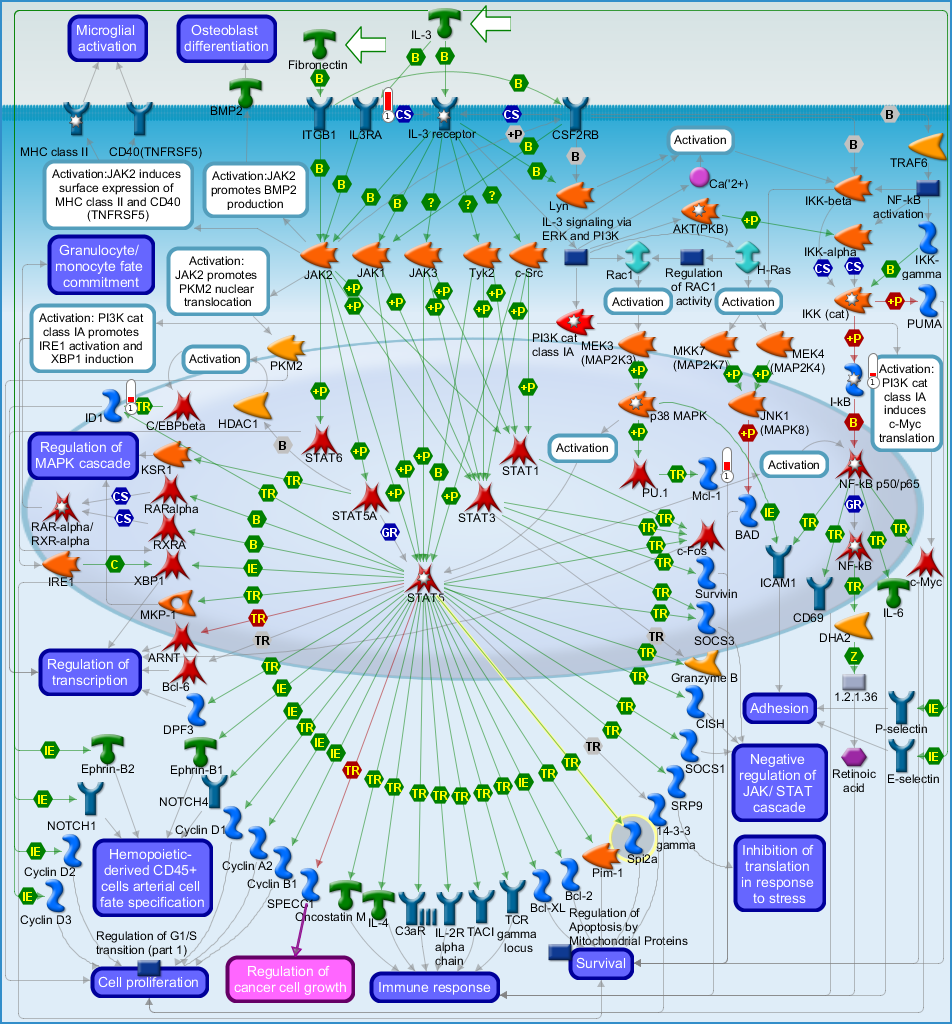


**Figure 7.** The fifth scored map (map with the fifth lowest p‑value) based on the enrichment distribution sorted by 'Statistically significant Maps' set. Experimental data from all files is linked to and visualized on the maps as thermometer‑like figures. Up‑ward thermometers have red color and indicate up‑regulated signals and down‑ward (blue) ones indicate down‑regulated expression levels of the genes.

### Process Networks ([TOC](#TOC_table))

The content of these cellular and molecular processes is defined and annotated by Clarivate scientists. Each process represents a pre‑set network of protein interactions characteristic for the process.


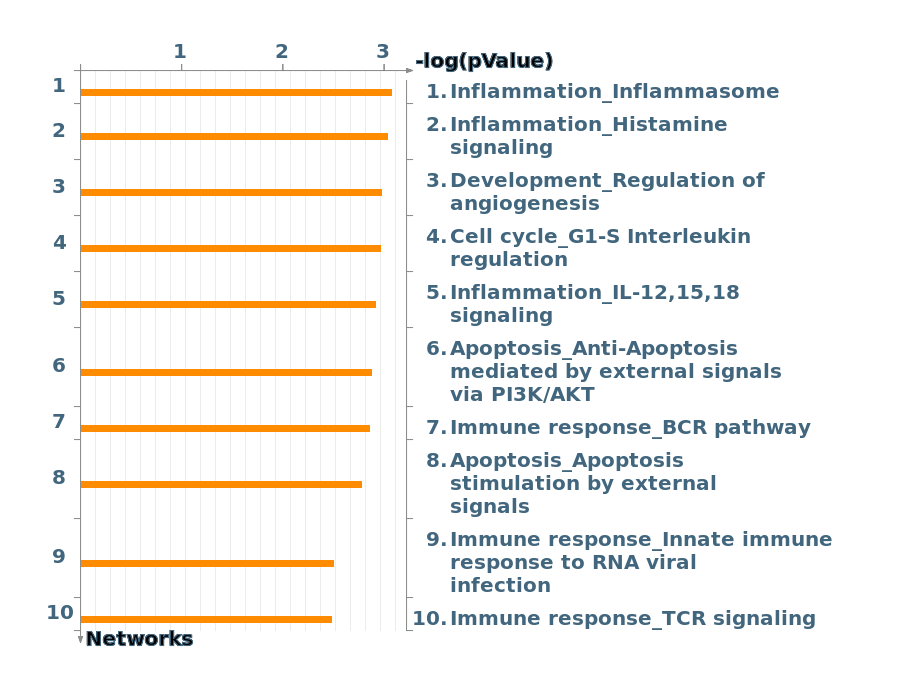


**Figure 8.** Process Networks. Sorting is done for the 'Statistically significant Networks'.

### Diseases (by Biomarkers) ([TOC](#TOC_table))

Disease folders are organized into a hierarchical tree. Gene content may very greatly between such complex diseases as cancers and some Mendelian diseases. Also, coverage of different diseases in literature is skewed. These two factors may affect p‑value prioritization for diseases.


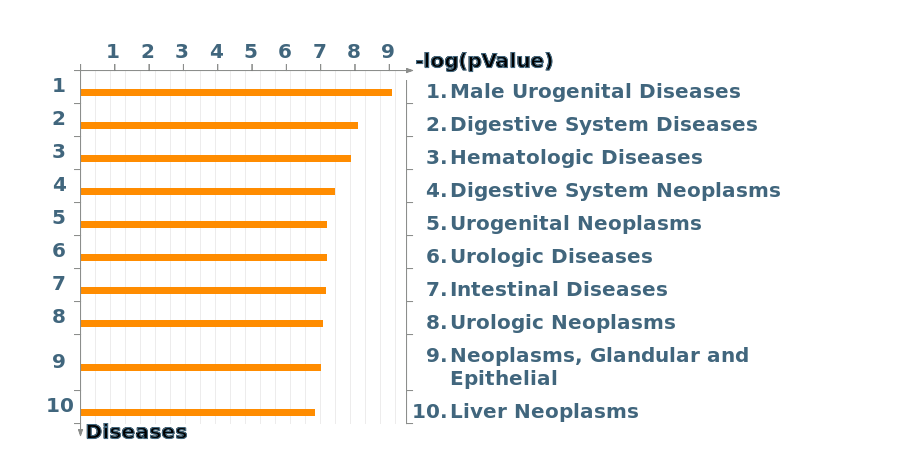


**Figure 9.** Diseases (by Biomarkers). Sorting is done for the 'Statistically significant Diseases'.

### GO Processes ([TOC](#TOC_table))

These are Gene Ontology (GO) cellular processes. As most GO processes have no gene/protein content, the "empty terms" are excluded from p‑value calculations.


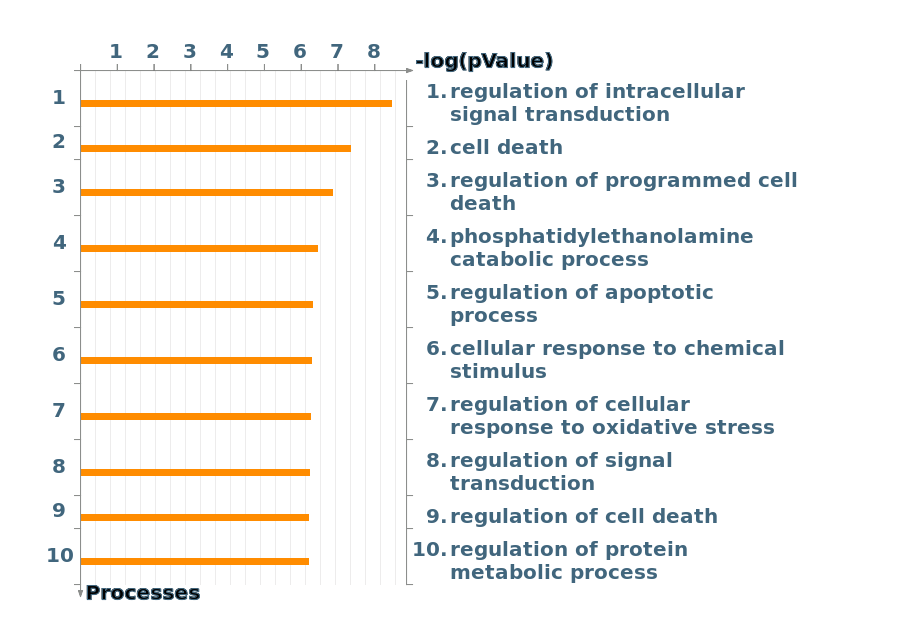


**Figure 10.** GO Processes. Sorting is done for the 'Statistically significant Processes'.

Lightsteelblue

**Enrichment Analysis Workflow 1.0 Data Analysis Report**

*Server: portal.genego.com*

*Date: 2021‑12‑01*

*Name: NORWEGIAN UNIV OF SCI and TECH | Arnar Flatberg | arnar.flatberg@ntnu.no*

*Login: ntnu3*

Experiments

| 1. |  | Lightsteelblue |
| --- | --- | --- |

The experiments uploaded for comparative analysis

## Table of content:

**[•](#Bookmark_1)** [Enrichment analysis](#Bookmark_1)

**[•](#Bookmark_2)** [Pathway Maps](#Bookmark_2)

**[•](#Bookmark_3)** [Top maps (sorted by Statistically significant Maps)](#Bookmark_3)

**[•](#Bookmark_4)** [1. Map : Regulation of lipid metabolism_RXR‑dependent regulation of lipid metabolism via PPAR, RAR and VDR](#Bookmark_4)

**[•](#Bookmark_5)** [2. Map : Protein folding and maturation_Bradykinin / Kallidin maturation](#Bookmark_5)

**[•](#Bookmark_6)** [3. Map : Transcription_Sirtuin6 regulation and functions](#Bookmark_6)

**[•](#Bookmark_7)** [4. Map : Role of neuropeptides in pathogenesis of SCLC](#Bookmark_7)

**[•](#Bookmark_8)** [5. Map : CAR signaling via cross‑talk / Human Version](#Bookmark_8)

**[•](#Bookmark_9)** [Process Networks](#Bookmark_9)

**[•](#Bookmark_10)** [Diseases (by Biomarkers)](#Bookmark_10)

**[•](#Bookmark_11)** [GO Processes](#Bookmark_11)

## Enrichment analysis ([TOC](#TOC_table))

Enrichment analysis consists of matching gene IDs of possible targets for the "common", "similar" and "unique" sets with gene IDs in functional ontologies in MetaCore. The probability of a random intersection between a set of IDs the size of target list with ontology entities is estimated in p‑value of hypergeometric intersection. The lower p‑value means higher relevance of the entity to the dataset, which shows in higher rating for the entity.

Ontologies available for EA in Enrichment Analysis Workflow:

### Pathway Maps ([TOC](#TOC_table))

Canonical pathway maps represent a set of signaling and metabolic maps covering human in a comprehensive way. All maps are created by Clarivate scientists by a high‑quality manual curation process based on published peer‑reviewed literature. Experimental data is visualized on the maps as blue (for downregulation) and red (upregulation) histograms. The height of the histogram corresponds to the relative expression value for a particular gene/protein.


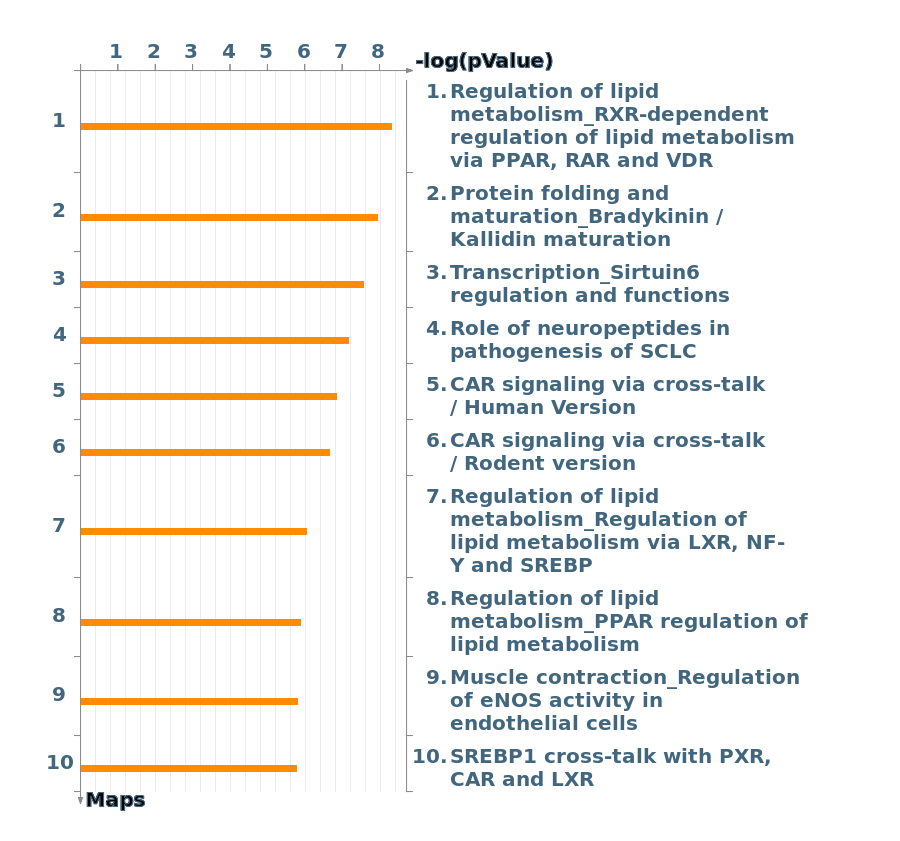


**Figure 2.** Pathway Maps. Sorting is done for the 'Statistically significant Maps'.

### Top maps (sorted by Statistically significant Maps)

**1. Map :** [Regulation of lipid metabolism_RXR‑dependent regulation of lipid metabolism via PPAR, RAR and VDR](https://portal.genego.com/cgi/imagemap.cgi?id=413)
([TOC](#TOC_table))


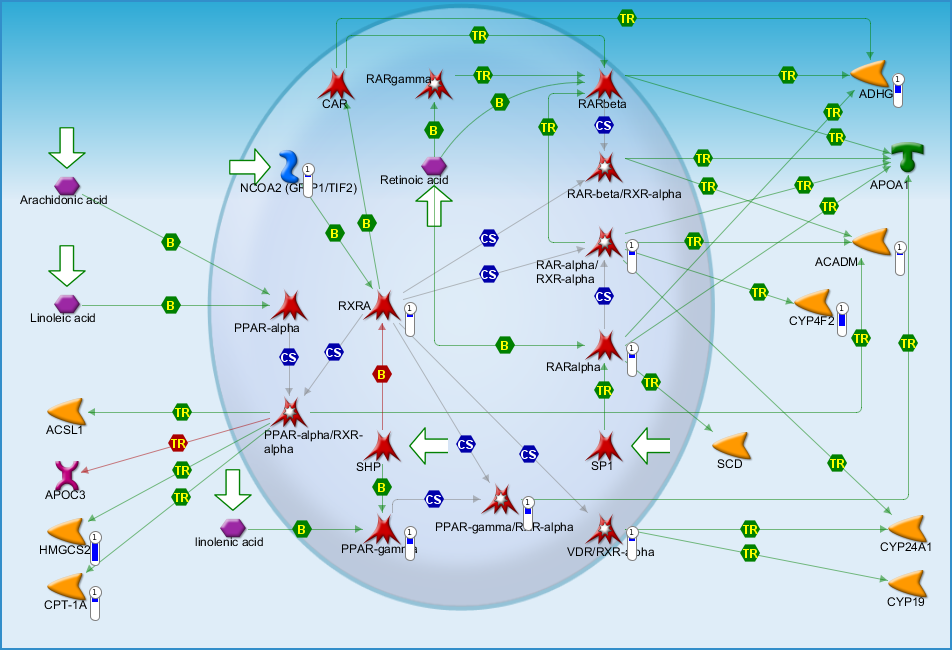


**Figure 3.** The top scored map (map with the the lowest p‑value) based on the enrichment distribution sorted by 'Statistically significant Maps' set. Experimental data from all files is linked to and visualized on the maps as thermometer‑like figures. Up‑ward thermometers have red color and indicate up‑regulated signals and down‑ward (blue) ones indicate down‑regulated expression levels of the genes.

**2. Map :** [Protein folding and maturation_Bradykinin / Kallidin maturation](https://portal.genego.com/cgi/imagemap.cgi?id=2659)
([TOC](#TOC_table))


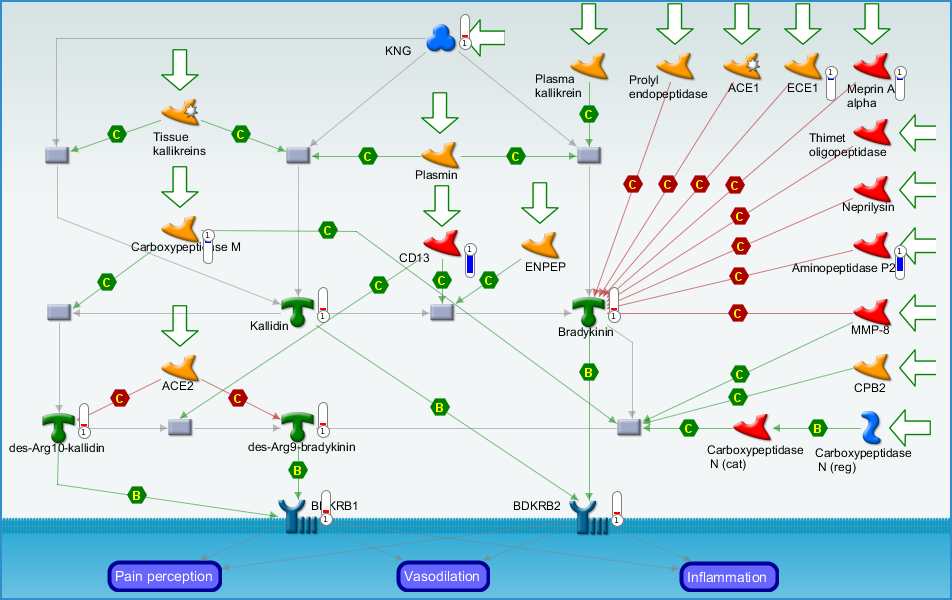


**Figure 4.** The second scored map (map with the second lowest p‑value) based on the enrichment distribution sorted by 'Statistically significant Maps' set. Experimental data from all files is linked to and visualized on the maps as thermometer‑like figures. Up‑ward thermometers have red color and indicate up‑regulated signals and down‑ward (blue) ones indicate down‑regulated expression levels of the genes.

**3. Map :** [Transcription_Sirtuin6 regulation and functions](https://portal.genego.com/cgi/imagemap.cgi?id=6935)
([TOC](#TOC_table))


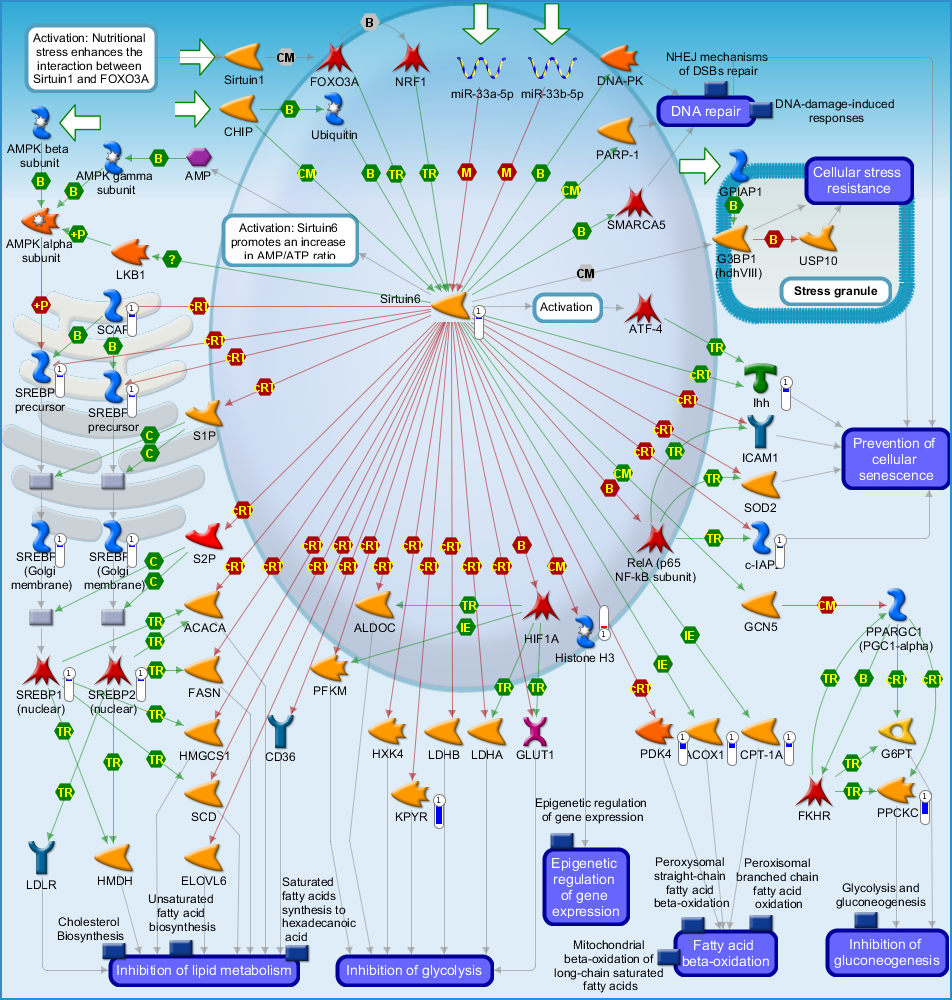


**Figure 5.** The third scored map (map with the third lowest p‑value) based on the enrichment distribution sorted by 'Statistically significant Maps' set. Experimental data from all files is linked to and visualized on the maps as thermometer‑like figures. Up‑ward thermometers have red color and indicate up‑regulated signals and down‑ward (blue) ones indicate down‑regulated expression levels of the genes.

**4. Map :** [Role of neuropeptides in pathogenesis of SCLC](https://portal.genego.com/cgi/imagemap.cgi?id=6765)
([TOC](#TOC_table))


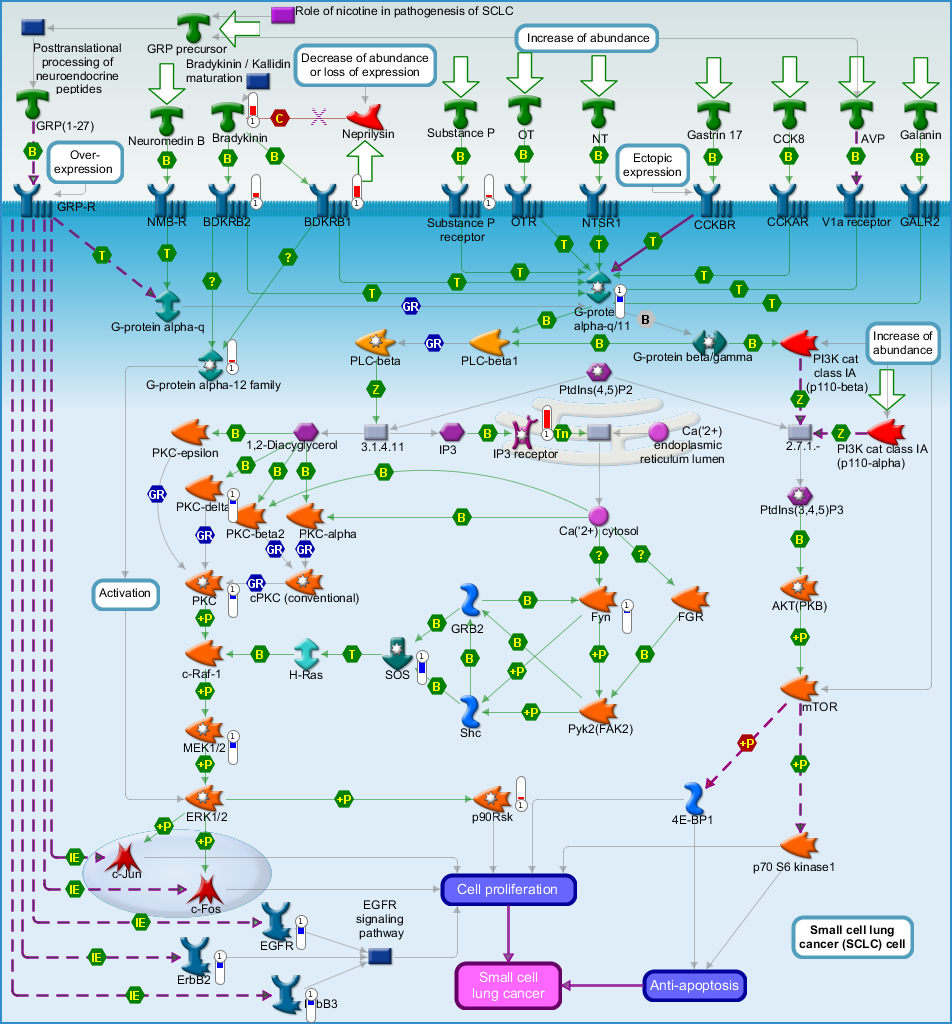


**Figure 6.** The fourth scored map (map with the fourth lowest p‑value) based on the enrichment distribution sorted by 'Statistically significant Maps' set. Experimental data from all files is linked to and visualized on the maps as thermometer‑like figures. Up‑ward thermometers have red color and indicate up‑regulated signals and down‑ward (blue) ones indicate down‑regulated expression levels of the genes.

**5. Map :** [CAR signaling via cross‑talk / Human Version](https://portal.genego.com/cgi/imagemap.cgi?id=2799)
([TOC](#TOC_table))


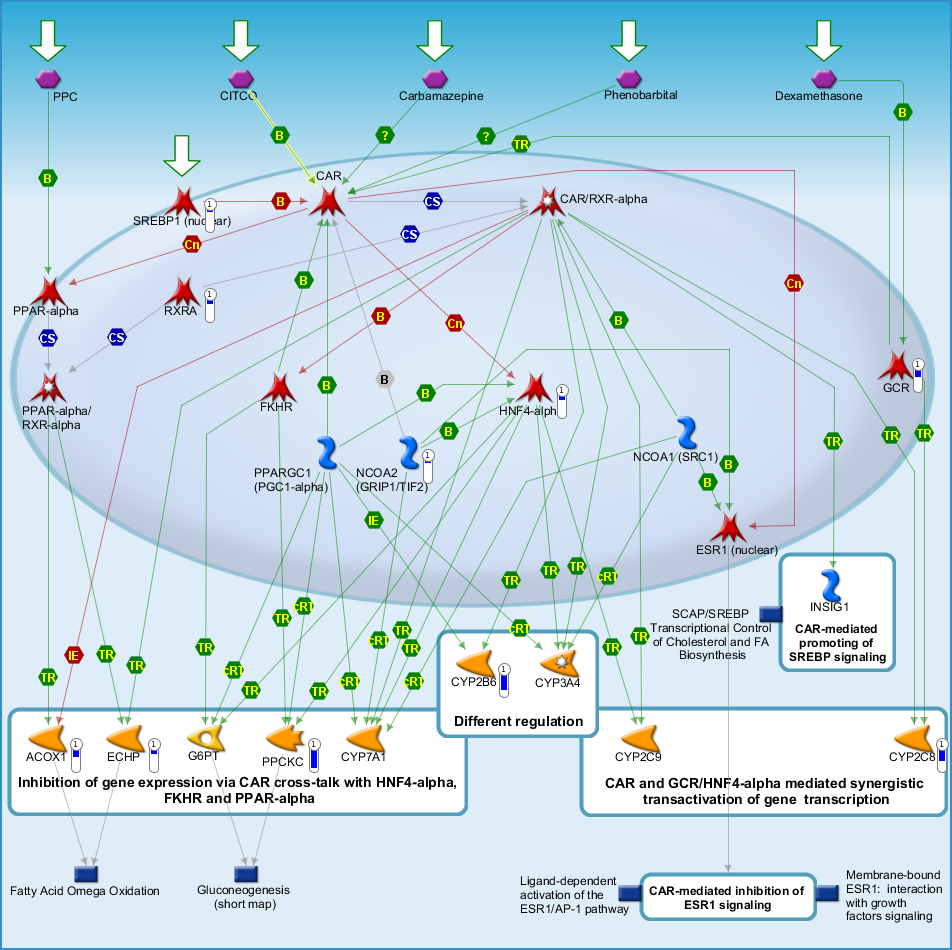


**Figure 7.** The fifth scored map (map with the fifth lowest p‑value) based on the enrichment distribution sorted by 'Statistically significant Maps' set. Experimental data from all files is linked to and visualized on the maps as thermometer‑like figures. Up‑ward thermometers have red color and indicate up‑regulated signals and down‑ward (blue) ones indicate down‑regulated expression levels of the genes.

### Process Networks ([TOC](#TOC_table))

The content of these cellular and molecular processes is defined and annotated by Clarivate scientists. Each process represents a pre‑set network of protein interactions characteristic for the process.


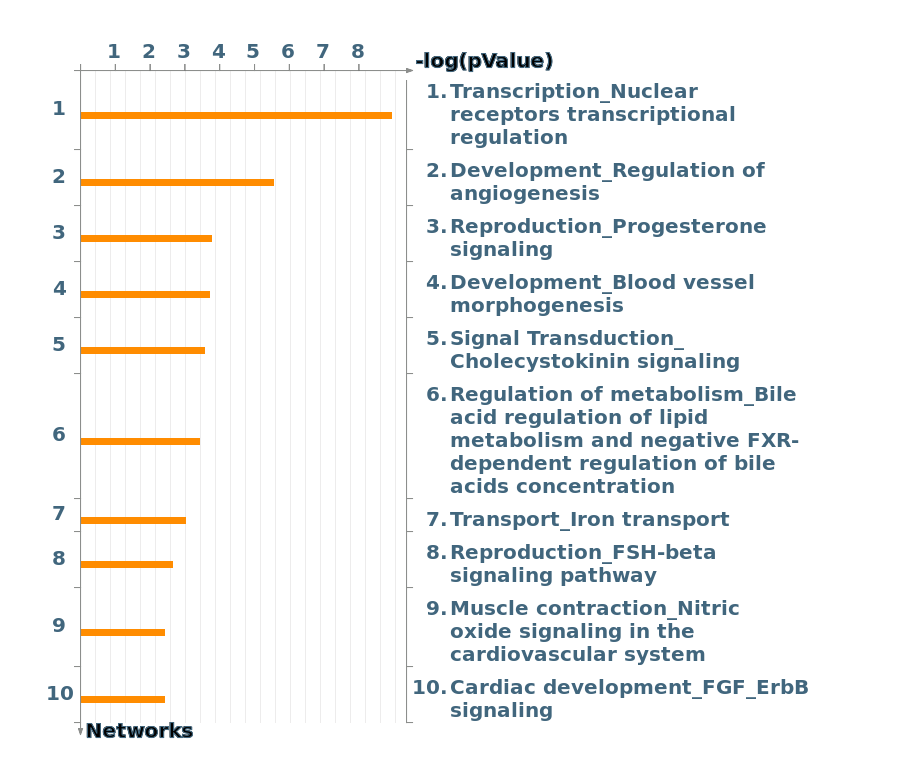


**Figure 8.** Process Networks. Sorting is done for the 'Statistically significant Networks'.

### Diseases (by Biomarkers) ([TOC](#TOC_table))

Disease folders are organized into a hierarchical tree. Gene content may very greatly between such complex diseases as cancers and some Mendelian diseases. Also, coverage of different diseases in literature is skewed. These two factors may affect p‑value prioritization for diseases.


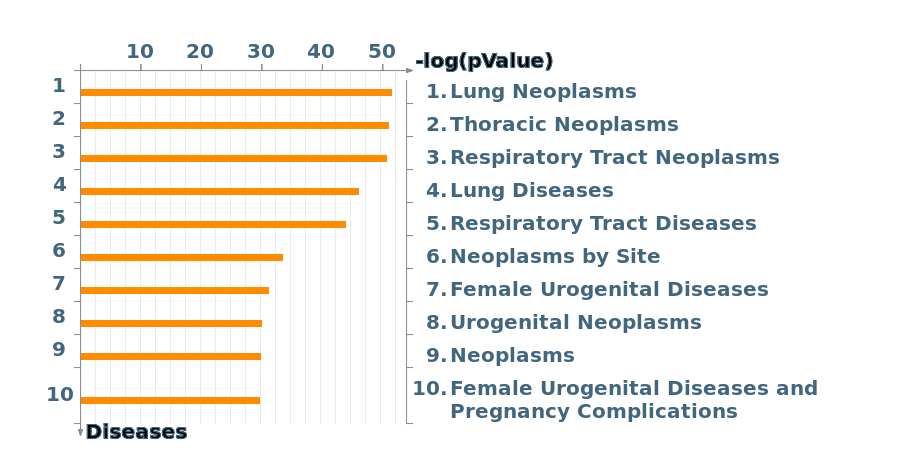


**Figure 9.** Diseases (by Biomarkers). Sorting is done for the 'Statistically significant Diseases'.

### GO Processes ([TOC](#TOC_table))

These are Gene Ontology (GO) cellular processes. As most GO processes have no gene/protein content, the "empty terms" are excluded from p‑value calculations.


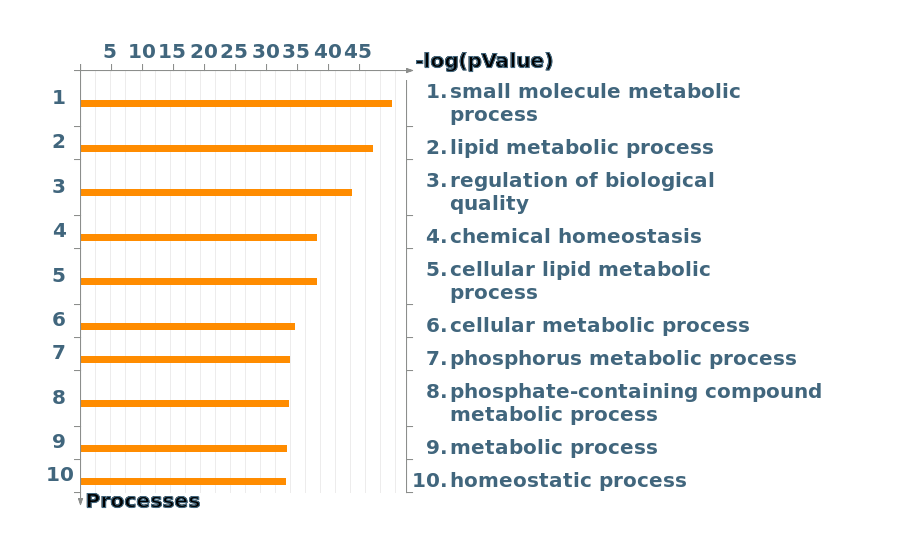


**Figure 10.** GO Processes. Sorting is done for the 'Statistically significant Processes'.

Orangered

**Enrichment Analysis Workflow 1.0 Data Analysis Report**

*Server: portal.genego.com*

*Date: 2021‑12‑01*

*Name: NORWEGIAN UNIV OF SCI and TECH | Arnar Flatberg | arnar.flatberg@ntnu.no*

*Login: ntnu3*

Experiments

| 1. |  | Orangered |
| --- | --- | --- |

The experiments uploaded for comparative analysis

## Table of content:

**[•](#Bookmark_1)** [Enrichment analysis](#Bookmark_1)

**[•](#Bookmark_2)** [Pathway Maps](#Bookmark_2)

**[•](#Bookmark_3)** [Top maps (sorted by Statistically significant Maps)](#Bookmark_3)

**[•](#Bookmark_4)** [1. Map : Cell adhesion_Integrin inside‑out signaling in neutrophils](#Bookmark_4)

**[•](#Bookmark_5)** [2. Map : Protein folding_Membrane trafficking and signal transduction of G‑alpha (i) heterotrimeric G‑protein](#Bookmark_5)

**[•](#Bookmark_6)** [3. Map : Chemotaxis_SDF‑1/ CXCR4‑induced chemotaxis of immune cells](#Bookmark_6)

**[•](#Bookmark_7)** [4. Map : G‑protein signaling_G‑Protein beta/gamma signaling cascades](#Bookmark_7)

**[•](#Bookmark_8)** [5. Map : Stem cells_Pancreatic cancer stem cells in tumor metastasis](#Bookmark_8)

**[•](#Bookmark_9)** [Process Networks](#Bookmark_9)

**[•](#Bookmark_10)** [Diseases (by Biomarkers)](#Bookmark_10)

**[•](#Bookmark_11)** [GO Processes](#Bookmark_11)

## Enrichment analysis ([TOC](#TOC_table))

Enrichment analysis consists of matching gene IDs of possible targets for the "common", "similar" and "unique" sets with gene IDs in functional ontologies in MetaCore. The probability of a random intersection between a set of IDs the size of target list with ontology entities is estimated in p‑value of hypergeometric intersection. The lower p‑value means higher relevance of the entity to the dataset, which shows in higher rating for the entity.

Ontologies available for EA in Enrichment Analysis Workflow:

### Pathway Maps ([TOC](#TOC_table))

Canonical pathway maps represent a set of signaling and metabolic maps covering human in a comprehensive way. All maps are created by Clarivate scientists by a high‑quality manual curation process based on published peer‑reviewed literature. Experimental data is visualized on the maps as blue (for downregulation) and red (upregulation) histograms. The height of the histogram corresponds to the relative expression value for a particular gene/protein.


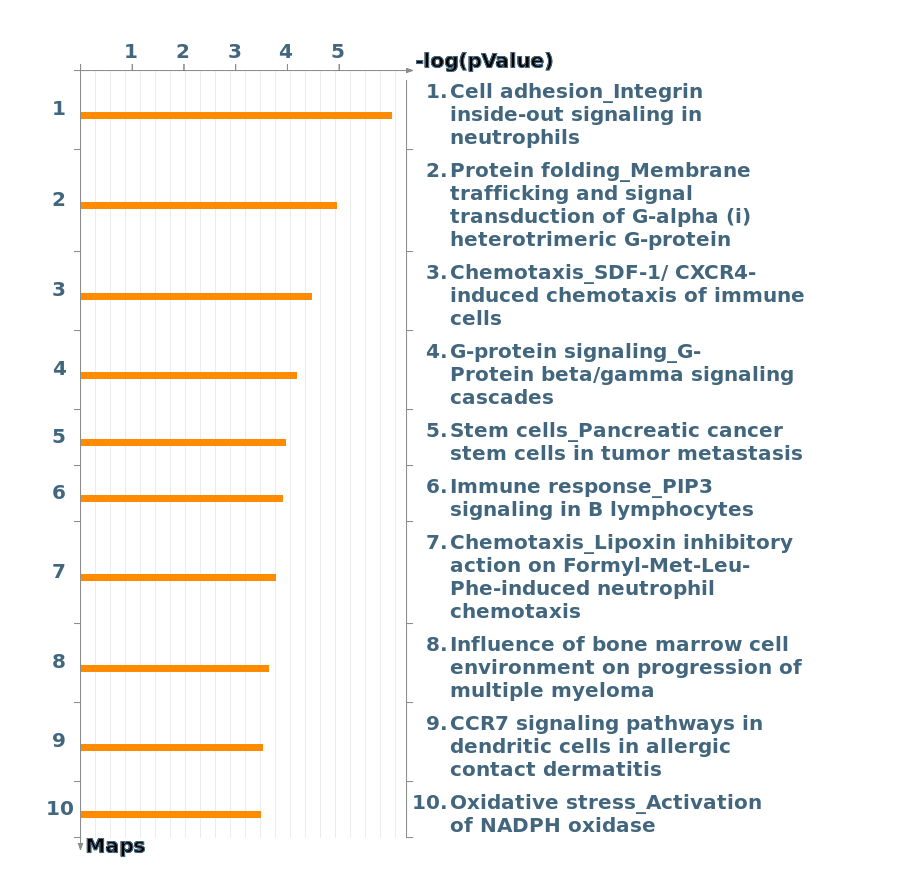


**Figure 2.** Pathway Maps. Sorting is done for the 'Statistically significant Maps'.

### Top maps (sorted by Statistically significant Maps)

**1. Map :** [Cell adhesion_Integrin inside‑out signaling in neutrophils](https://portal.genego.com/cgi/imagemap.cgi?id=7063)
([TOC](#TOC_table))


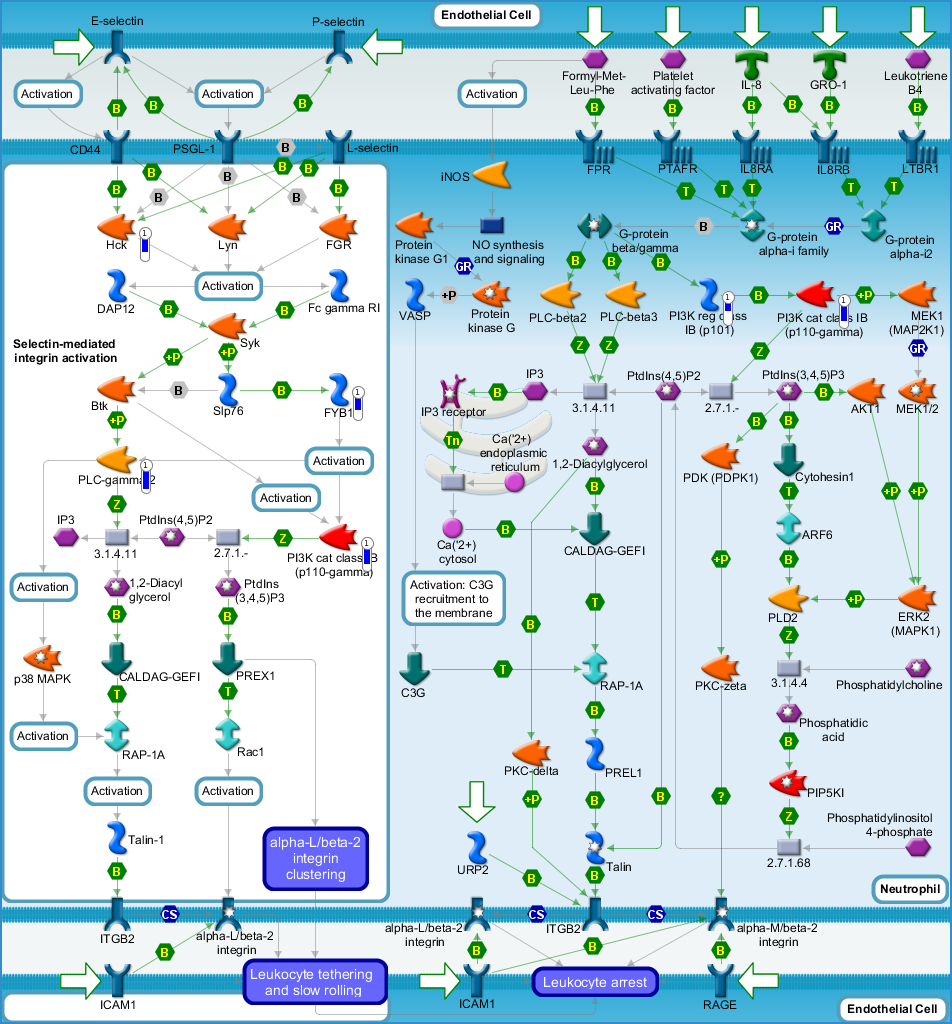


**Figure 3.** The top scored map (map with the the lowest p‑value) based on the enrichment distribution sorted by 'Statistically significant Maps' set. Experimental data from all files is linked to and visualized on the maps as thermometer‑like figures. Up‑ward thermometers have red color and indicate up‑regulated signals and down‑ward (blue) ones indicate down‑regulated expression levels of the genes.

**2. Map :** [Protein folding_Membrane trafficking and signal transduction of G‑alpha (i) heterotrimeric G‑protein](https://portal.genego.com/cgi/imagemap.cgi?id=457)
([TOC](#TOC_table))


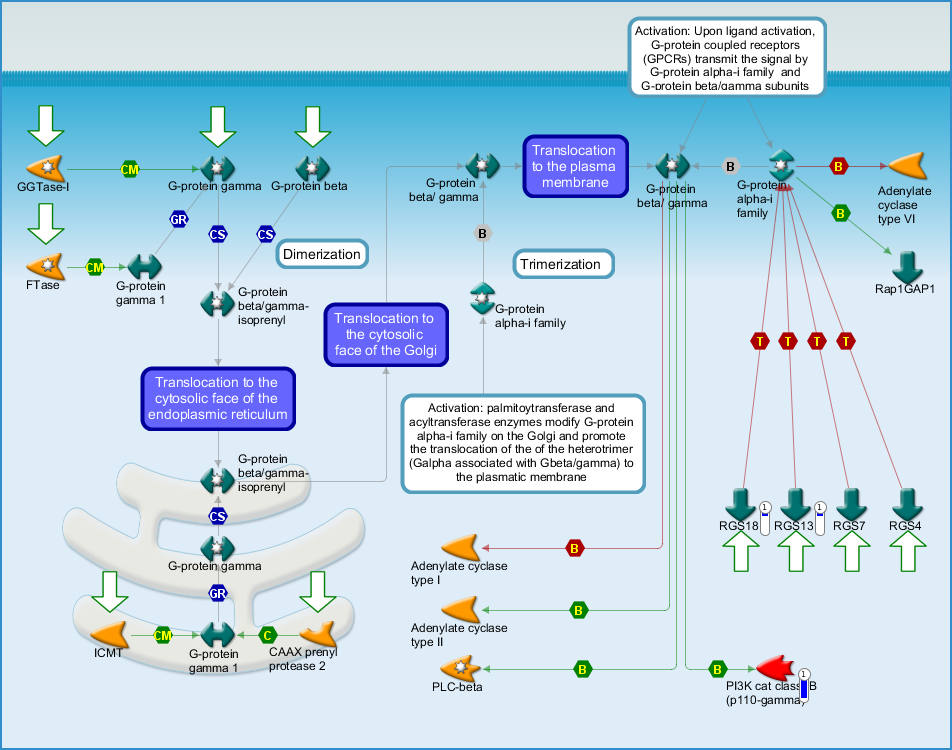


**Figure 4.** The second scored map (map with the second lowest p‑value) based on the enrichment distribution sorted by 'Statistically significant Maps' set. Experimental data from all files is linked to and visualized on the maps as thermometer‑like figures. Up‑ward thermometers have red color and indicate up‑regulated signals and down‑ward (blue) ones indicate down‑regulated expression levels of the genes.

**3. Map :** [Chemotaxis_SDF‑1/ CXCR4‑induced chemotaxis of immune cells](https://portal.genego.com/cgi/imagemap.cgi?id=7295)
([TOC](#TOC_table))


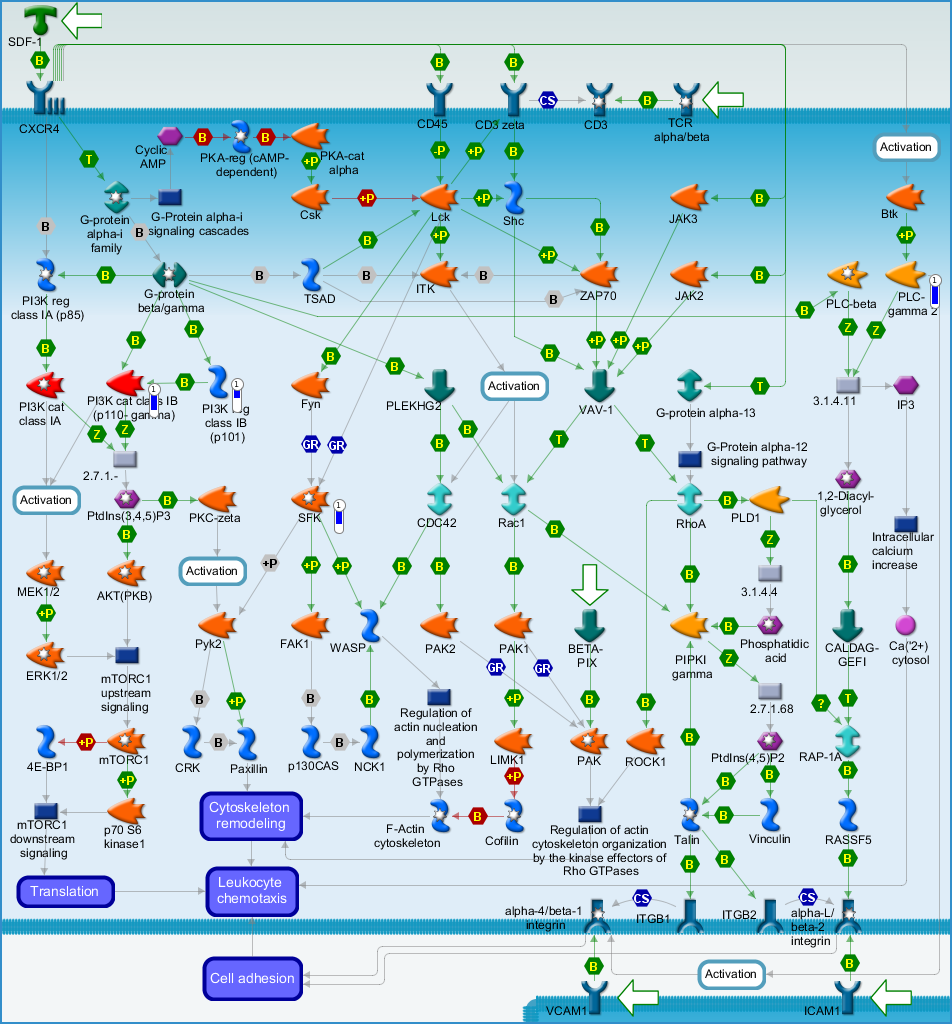


**Figure 5.** The third scored map (map with the third lowest p‑value) based on the enrichment distribution sorted by 'Statistically significant Maps' set. Experimental data from all files is linked to and visualized on the maps as thermometer‑like figures. Up‑ward thermometers have red color and indicate up‑regulated signals and down‑ward (blue) ones indicate down‑regulated expression levels of the genes.

**4. Map :** [G‑protein signaling_G‑Protein beta/gamma signaling cascades](https://portal.genego.com/cgi/imagemap.cgi?id=641)
([TOC](#TOC_table))


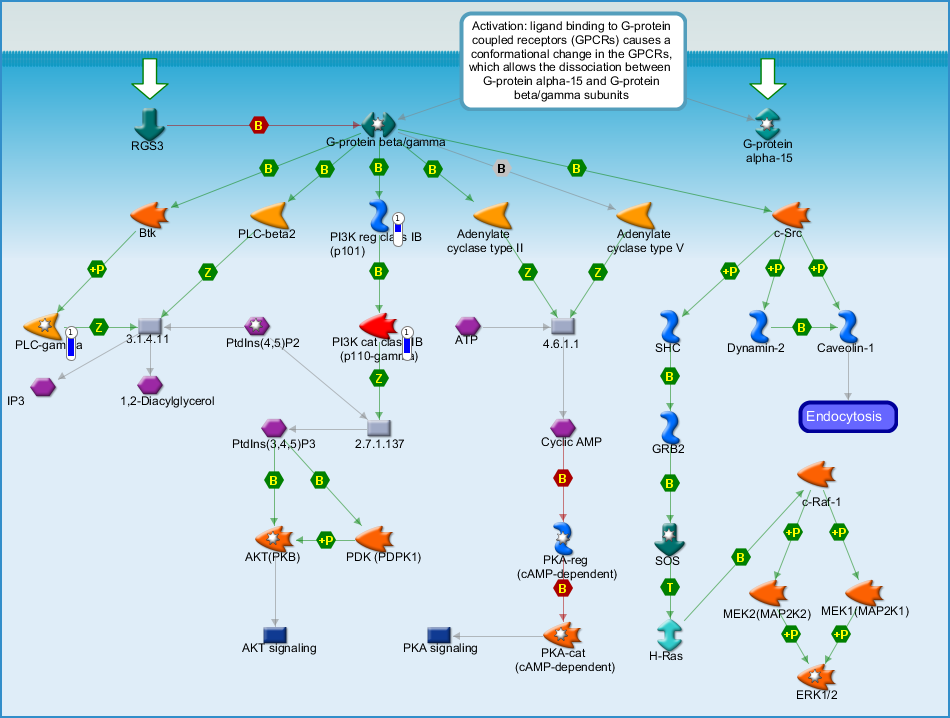


**Figure 6.** The fourth scored map (map with the fourth lowest p‑value) based on the enrichment distribution sorted by 'Statistically significant Maps' set. Experimental data from all files is linked to and visualized on the maps as thermometer‑like figures. Up‑ward thermometers have red color and indicate up‑regulated signals and down‑ward (blue) ones indicate down‑regulated expression levels of the genes.

**5. Map :** [Stem cells_Pancreatic cancer stem cells in tumor metastasis](https://portal.genego.com/cgi/imagemap.cgi?id=3161)
([TOC](#TOC_table))


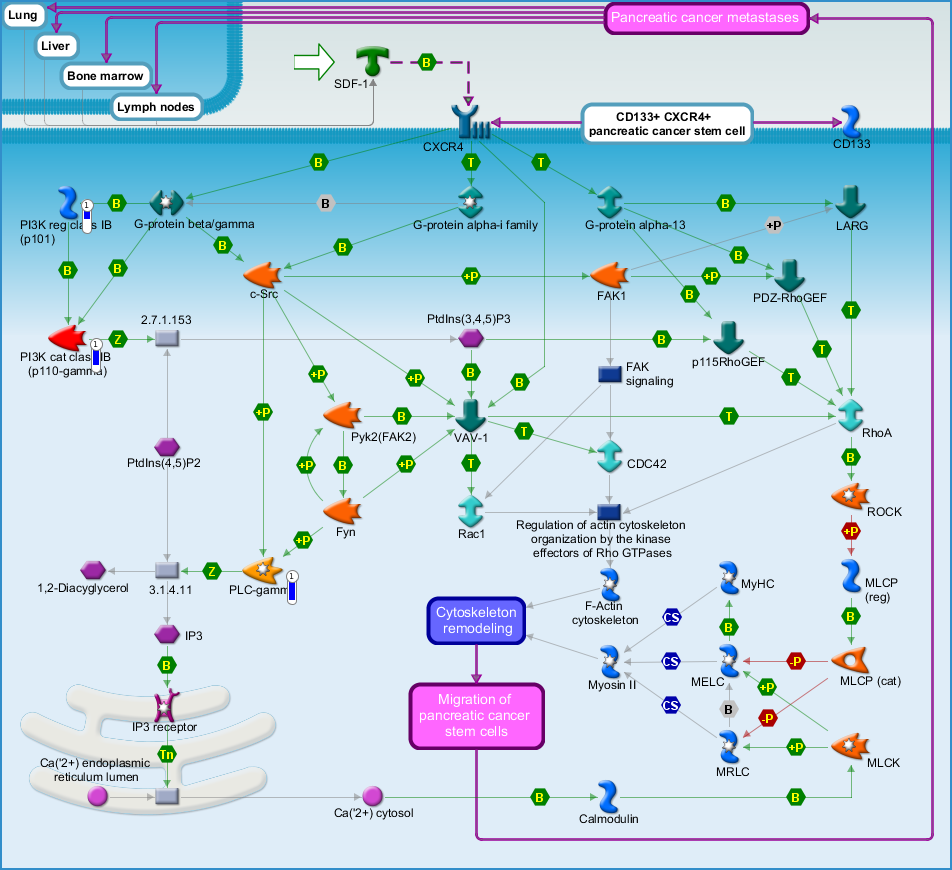


**Figure 7.** The fifth scored map (map with the fifth lowest p‑value) based on the enrichment distribution sorted by 'Statistically significant Maps' set. Experimental data from all files is linked to and visualized on the maps as thermometer‑like figures. Up‑ward thermometers have red color and indicate up‑regulated signals and down‑ward (blue) ones indicate down‑regulated expression levels of the genes.

### Process Networks ([TOC](#TOC_table))

The content of these cellular and molecular processes is defined and annotated by Clarivate scientists. Each process represents a pre‑set network of protein interactions characteristic for the process.


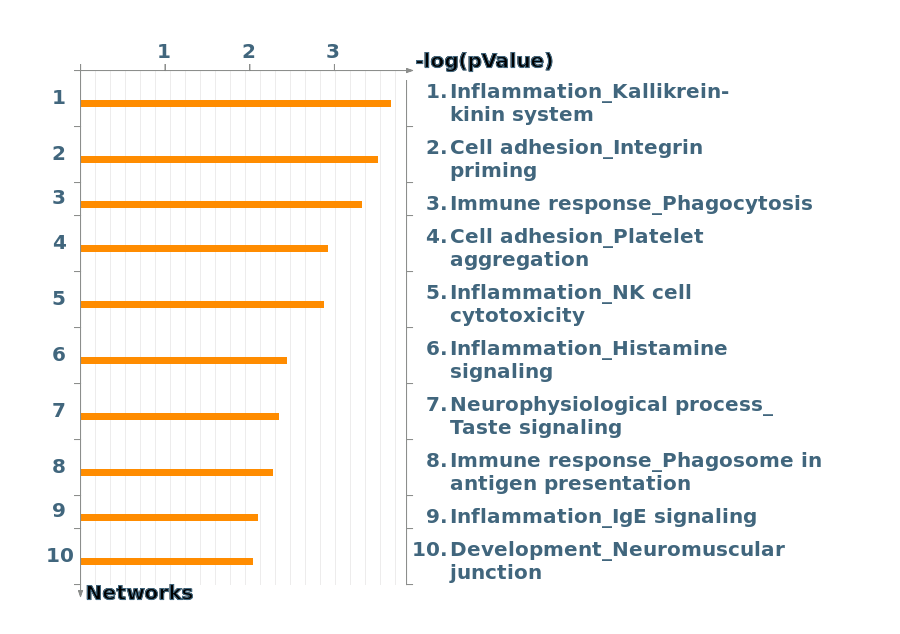


**Figure 8.** Process Networks. Sorting is done for the 'Statistically significant Networks'.

### Diseases (by Biomarkers) ([TOC](#TOC_table))

Disease folders are organized into a hierarchical tree. Gene content may very greatly between such complex diseases as cancers and some Mendelian diseases. Also, coverage of different diseases in literature is skewed. These two factors may affect p‑value prioritization for diseases.


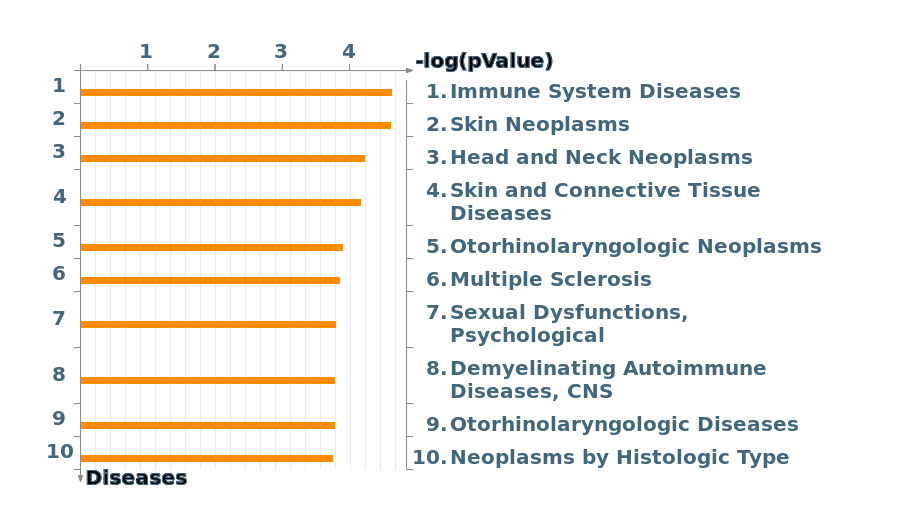


**Figure 9.** Diseases (by Biomarkers). Sorting is done for the 'Statistically significant Diseases'.

### GO Processes ([TOC](#TOC_table))

These are Gene Ontology (GO) cellular processes. As most GO processes have no gene/protein content, the "empty terms" are excluded from p‑value calculations.


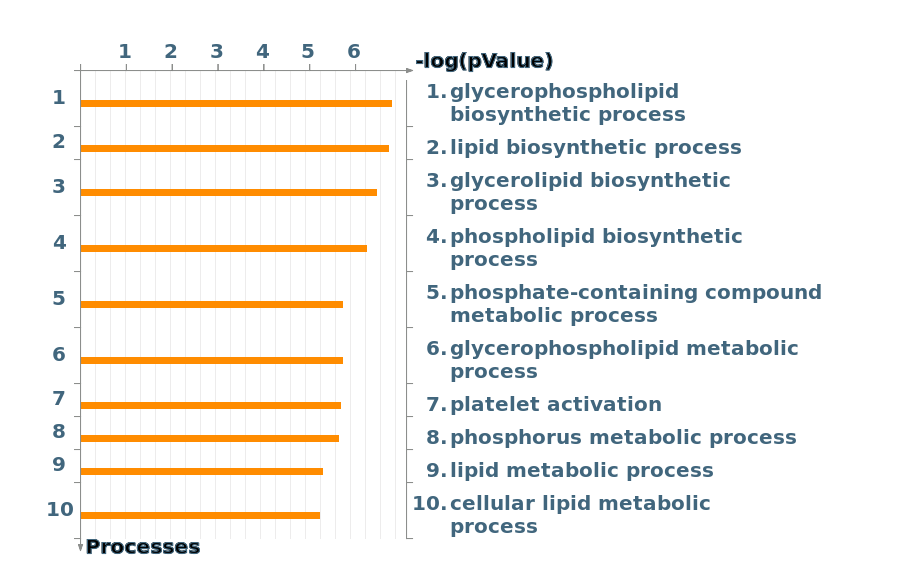


**Figure 10.** GO Processes. Sorting is done for the 'Statistically significant Processes'.
